# Using physiological biomarkers in forensic psychiatry: a scoping review

**DOI:** 10.3389/fpsyt.2025.1580615

**Published:** 2025-04-29

**Authors:** Jenthe Mens, Erik Masthoff, Stefan Bogaerts, Pauline Heus

**Affiliations:** ^1^ Department of Developmental Psychology, Tilburg University, Tilburg, Netherlands; ^2^ Fivoor Science and Treatment Innovation, Fivoor, Rotterdam, Netherlands; ^3^ Cochrane Netherlands, University Medical Centre Utrecht, Utrecht University, Utrecht, Netherlands; ^4^ Julius Center for Health Sciences and Primary Care, University Medical Center Utrecht, Utrecht University, Utrecht, Netherlands

**Keywords:** forensic psychiatry, physiological biomarkers, scoping review, biomarker function, psychopathology

## Abstract

Forensic psychiatry aims to reduce criminogenic risks and enhance societal safety. While effective at a macro level, crime recidivism rates among forensic patients remain substantial. This underlines the need for innovation, with growing interest in the use of physiological biomarkers. To identify the extent (size), range, (variety), and nature (characteristics) of evidence on the use of physiological biomarkers in forensic psychiatry, a systematic scoping review was conducted following JBI methodology and PRISMA-ScR guidance. Data on study characteristics and results were extracted by two independent reviewers from 431 primary research studies published in scientific journals and dissertations. Most studies were conducted in North America (53.4%) and Europe (41.3%). The majority employed an observational design (95.6%) and were cross-sectional (87.7%). Studies predominantly focused on males (84.9%) and adults (85.9%). The most common diagnoses were psychopathy/antisocial personality disorder (51.7%) and sexual disorders (21.8%). Brain activity served as a biomarker outcome in 51.3% of studies, followed by peripheral sympathetic arousal (29.2%) and peripheral sexual arousal (13.8%). Biomarker assessment methods reflected these findings. Etiologic biomarker functions were most common (77.2%), followed by diagnostic functions (12.7%). Findings reveal several gaps in the existing scientific literature. Specifically, more experimental and longitudinal research is needed to integrate physiological biomarkers into e.g., interventions, effect monitoring, and (risk) assessment. Also, a greater focus on juveniles, patients with psychotic and substance use disorders, and the use of newer biomarker assessment methods measuring peripheral arousal is essential to advance the field.

**Systematic review registration:**

https://osf.io/, 10.17605/OSF.IO/46QBU.

## 1 Introduction

Certain types of psychopathology, such as schizophrenia, substance use disorder (SUD), and antisocial personality disorder, are significant risk factors for (recurrent) delinquent behavior (1–3). Forensic psychiatry aims to reduce criminogenic risks, thereby enhancing societal safety. Although this works at a macro level (4), the current selection of evidence-based interventions remains limited (5) and recidivism rates among forensic patients are still substantial. This underlines the need for innovation in developing new treatment (evaluation) tools, assessment strategies, and risk prediction methods. Much of this potential lies in biomedical science and technology. Over the past decades, advancements in these areas have significantly improved the understanding of disease mechanisms, treatment options, and outcomes in most medical disciplines. However, psychiatry, and particularly forensic psychiatry, has been slower to adopt these innovations. Traditionally, it has relied on subjective symptom presentations and interpretations of observed behavior for assessment, diagnosis, therapy, and prognostication (6). Forensic patients, however, are prone to socially desirable responses (7), raising concerns about the validity of current assessment and evaluation methods. Because of this, there is increasing attention to neurobiological factors associated with criminal behavior. The merged role of biomarkers in forensic psychiatry provides a more objective and potentially reliable assessment method (8, 9).

A biomarker is an indicator of normal or pathological biological processes or responses to exposure or (therapeutic) interventions (10). A common way to categorize biomarkers is by their clinical application (11, 12). The *Biomarkers, EndpointS, and other Tools* (BEST) glossary defines seven biomarker categories: susceptibility/risk, diagnostic, monitoring, prognostic, predictive, pharmacodynamic/response, and safety (10). Additionally, biomarkers can be classified based on their characteristics. For instance, the BEST glossary (10) and Califf (12) distinguish molecular, histologic, radiographic, and physiologic biomarkers, whereas Bodhagi et al. (11) propose a division into cellular, imaging, and molecular biomarkers, further subdividing the latter into genetic, protein, and chemical biomarkers.

To our knowledge, no scoping review has systematically examined the use of biomarkers in forensic psychiatry. The current evidence mapping regarding this subject remains very limited. While two narrative reviews exist regarding biomarkers for paedophilia and potential neurocognitive markers for recidivism prediction, these were not conducted systematically (8, 13). Additionally, other systematic reviews, such as those on objective predictors of outcome in forensic mental health services and on eye tracking and paedophilia, are outdated or overly narrow in scope (9, 14). Consequently, the available reviews are insufficient for understanding the role of biomarkers in forensic psychiatry.

This scoping review focused on physiological biomarkers (e.g., skin conductance, heart rate variability, and brain activity), as they are particularly relevant for clinical forensic practice. Unlike static biomarkers (e.g., genetic), physiological biomarkers are dynamic and changeable, offering actionable outcomes for prevention, assessment, and treatment. They are also non-invasive, easily measurable, and practical for ongoing assessment and intervention in forensic settings. Thus, this scoping review aimed to identify the extent (size), range (variety), and nature (characteristics) of evidence on the use of physiological biomarkers in forensic psychiatry. Sub-questions were: (1) How has research on physiological biomarkers in forensic psychiatry evolved over time?; (2) What clinical applications are addressed in this research?; (3) How are studies on physiological biomarkers distributed by offender type and diagnosis category? Due to the exploratory nature of the research, no *a priori* hypotheses were formulated.

## 2 Methods

### 2.1 Study descriptor

This study followed the JBI methodology for scoping reviews (15) and adhered to a protocol registered on the Open Science Framework (https://osf.io/46qbu/), with changes recorded in this paper. A descriptive approach is taken; no quality assessment of the included studies was conducted. The study is complied with following the Preferred Reporting Items for Systematic Reviews and Meta-Analyses extension for Scoping Reviews (PRISMA-ScR) checklist (16).

### 2.2 Participants

This scoping review included studies on forensic patients, defined as individuals diagnosed with a psychiatric disorder who were charged with or convicted of a criminal offense, without restrictions on gender, age, or ethnicity. Studies involving offenders with conditions like antisocial personality disorder, psychopathy, or paedophilic disorder were eligible. However, studies on individuals with transgressive behavior without a psychiatric disorder and/or ‘offender’ status were excluded. Research involving forensic psychiatry staff was also not considered.

#### 2.2.1 Context

This review selected studies conducted in forensic psychiatry settings, including forensic in- and outpatient clinics, correctional facilities (including prisons, juvenile correctional facilities, and probation supervision), and other professional environments where forensic patients were reported.

#### 2.2.2 Biomarkers

This review included studies reporting on the use of physiological biomarkers in forensic psychiatry, whether measured with imaging techniques or not. Anatomical/histological and molecular biomarkers (genetic, protein, chemical) were excluded unless studied in conjunction with physiological biomarkers. Physiological biomarkers were defined as measurable, dynamic indicators of the body’s physiological or pathological status. Examples included electrophysiological and hemodynamic brain activity assessed with EEG, MEG, fMRI, or SPECT, but not brain metabolism (which concerns biochemistry) measured with PET. Peripheral sympathetic arousal indicators, such as sexual arousal and eye movements, were also considered physiological biomarkers. For this review, the FDA and NIH (10) biomarker categories were adapted to suit the forensic psychiatric context, resulting in six functions and definitions: (1) etiologic: biomarker is an element in the explanatory model for the association between psychopathology and delinquent behavior; (2) diagnostic: biomarker is used to detect or confirm the presence of (a subtype of) psychopathology or condition status (e.g., arousal); (3) monitoring: biomarker is used to evaluate the course of patient functioning, including intervention effectiveness; (4) intervention: biomarker is part of an intervention (such as a training using biofeedback); (5) prognostic: biomarker is used to estimate the likelihood of crime recidivism; (6) predictive: biomarker is used to predict which patients benefit from certain interventions (responsiveness).

#### 2.2.3 Studies

This scoping review included primary research (original studies) published in scientific journals and dissertations. Systematic reviews and meta-analyses were excluded but served as sources for identifying primary research (see below). Narrative reviews, conference papers, book chapters, and grey literature were excluded for feasibility because they were not deemed essential for the review’s purpose. Quantitative, qualitative, and mixed-method studies were considered, including observational (descriptive, exploratory, analytical designs), experimental (randomized controlled trials, non-randomized controlled trials, quasi-experimental studies), cross-sectional, and longitudinal studies. Searches covered all dates up to September 1, 2023, with no language restrictions. Any translation issues encountered were manageable.

#### 2.2.4 Search strategy

The search strategy aimed to find published primary research studies using a three-step search strategy. First, a limited search of MEDLINE (PubMed) and PsycINFO was conducted to retrieve relevant publications. Titles, abstracts, and index terms from these articles were analyzed to develop (various iterations) a full search strategy for MEDLINE (see **Supplementary Material Appendix A**). Second, the final search strategy, using all identified keywords and index terms, was applied across all targeted databases (see below). For Google Scholar, a modified query with fewer characters and no truncation was used (see **Supplementary Material Appendix B**). Third, the reference lists of systematic reviews and meta-analyses, excluded during full-text screening, were searched for additional studies, deviating from the original protocol. However, the reference lists of included studies and excluded narrative reviews were not searched due to the substantial number of included studies and evidence saturation achieved after the initial steps.

#### 2.2.5 Information sources

A comprehensive search was conducted through the following scientific databases: MEDLINE (PubMed), PsycINFO, Embase, Social Science Citation Index (Web of Science), Scopus (for citation tracking), Cochrane Library, and Google Scholar.

#### 2.2.6 Study selection

All identified records (for Google Scholar the top 980) were uploaded and processed in EndNote 20 with duplicates removed. The records were then exported to the systematic review platform Rayyan (Qatar Computing Research Institute, Doha, Qatar). Titles and abstracts were screened by two reviewers (authors JM and EM) against the inclusion criteria. To optimize selection integrity, the first 50 publications were assessed jointly to establish consensus on interpreting and applying the inclusion criteria. Subsequently, 500 studies were screened on titles and abstracts independently by both reviewers and the ratings were compared. Strong inter-rater reliability was achieved (Cohen’s kappa = .94) (17), after which the remaining records were divided between the reviewers for independent assessment. Any uncertainties were resolved through cross-checks. The selection of the double-assessed publications was determined during biweekly consensus meetings. Next, both reviewers independently assessed the full text of the first 60 selected citations in detail against the inclusion criteria. Again, inter-rater reliability remained near perfect (Cohen’s kappa = .97) (17). The remaining publications were again divided and assessed by one of the two reviewers. Doubts or disagreements were resolved through cross-checks, followed by joint decision-making during consensus meetings.

#### 2.2.7 Data extraction

Data from studies included in the scoping review were extracted by two independent reviewers (JM and EM) using a data extraction tool developed by the researchers to align with the review’s aim. This tool was designed based on the JBI instrument for extracting study details, characteristics, and results (15). JM and EM each created a draft, which was integrated into a single tool after a consensus discussion, with additional input and critical reflection from a third researcher (PH). The tool was piloted on five relevant papers retrieved during the first search step, leading to minor adjustments. Further refinements were made during the data extraction process following consensus consultations between JM and EM with PH’s input. In particular, several variables were reformulated or made more concrete. Furthermore, the variable ‘stimulus/task’ representing the provocation used during biomarker assessment, was added. The final version of the data extraction tool is available as **Supplementary Material Appendix C**. To ensure accuracy and minimize bias, data extraction was cross-checked by the reviewers in 10% of the studies. Inconclusive and unclear data were excluded from extraction.

## 3 Results

**Figure 1:**
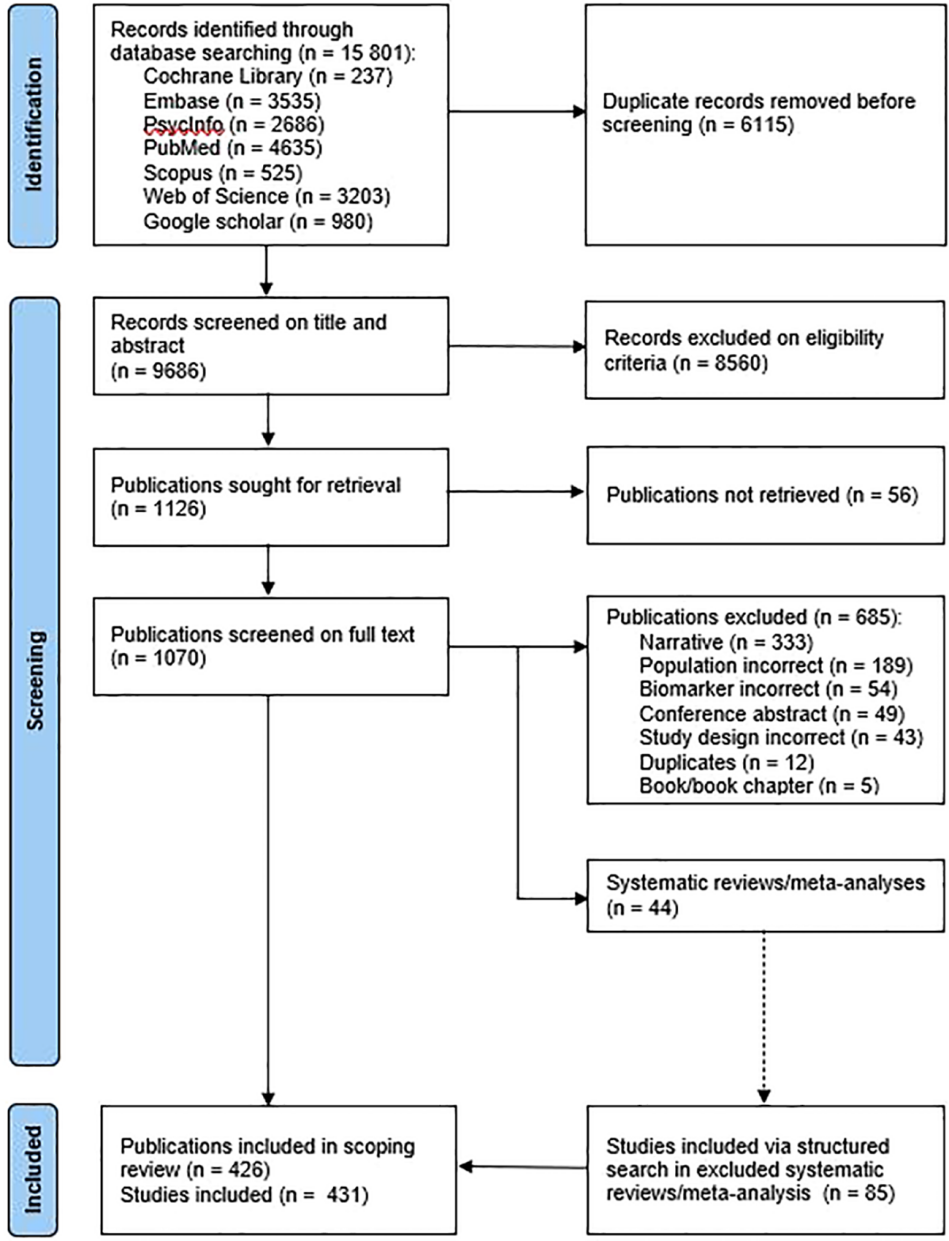
Study selection.

A total of 15,801 records were retrieved from the database searches. After removing duplicates, 9,686 records were screened on titles and abstracts. Subsequently, 1,070 publications were assessed for full-text eligibility. At the end of the selection process, 431 studies from 426 publications (some publications contained more than one qualifying study) were included in the scoping review. From these 431 studies, 85 were added via a structured reference list search of excluded systematic reviews and meta-analyses (**Figure 1**).

**Supplemantay Table 1** shows the data extracted from all included studies, covering contextual, design, and substantive aspects, supplemented with brief summaries of study aims and conclusions (15, 16, 18–445). The latter is provided to clearly and concisely describe the content of the studies without adding any qualitative judgment.

**Figure 2:**
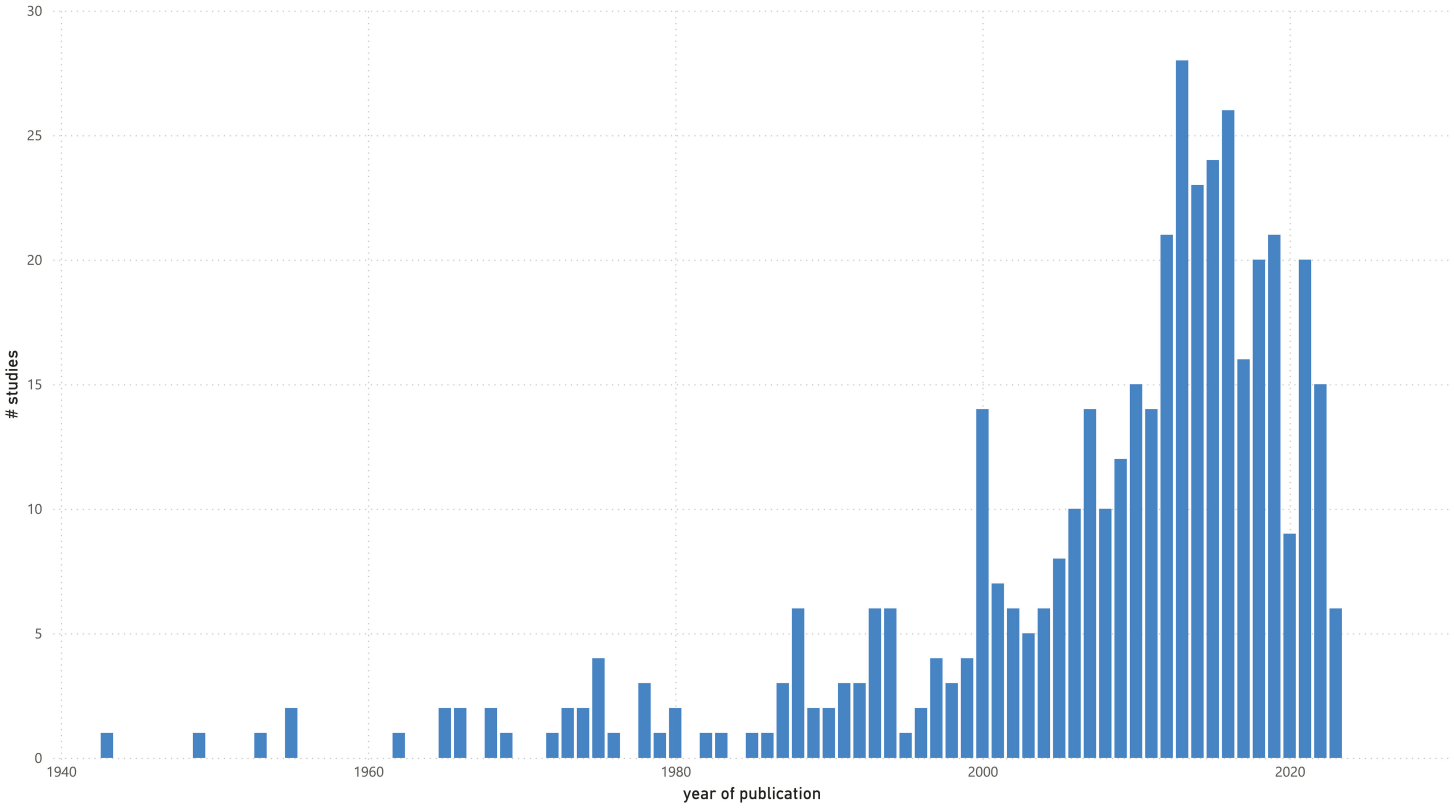
Number of studies by year of publication.

The included studies were conducted between 1943 and September 1, 2023 (date the databases were searched). Until 2000, the number of studies was limited to 78 (18.1%). Thereafter, there was an exponential increase of 92 studies (21.3%) until 2010 and 261 studies (60.6%) until the mentioned search date (**Figure 2**).

**Figure 3:**
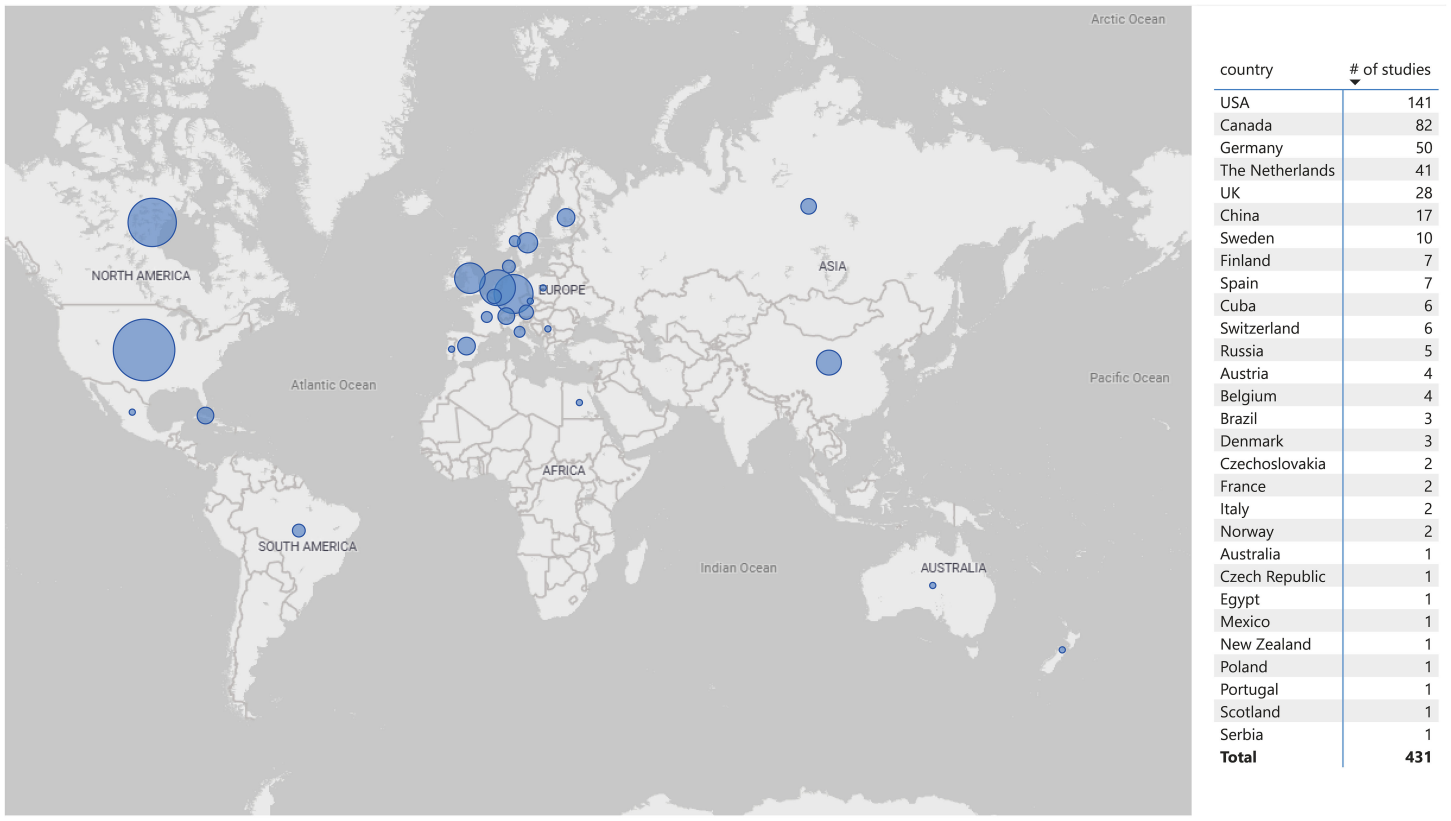
Countries in which the included studies were conducted.

Most studies were conducted in North America (53.4%) and Europe (41.3%). The top six countries (> 10 studies) were the United States, Canada, Germany, the Netherlands, the United Kingdom, and China, accounting for 83.3% of the studies (**Figure 3**).

**Table 1** presents the aggregated characteristics of the included studies, study populations, and control groups where applicable. The vast majority of studies had an observational design (95.6%), with case-control (62.6%) and cross-sectional (24.1%) studies being the most common. In contrast, (quasi) experimental research with physiological biomarkers in forensic psychiatry was scarce. In addition, most studies (87.7%) were not longitudinal. The included studies were mainly conducted in correctional facilities (45.6%) and inpatient clinics (34.2%), with fewer studies conducted in outpatient settings (16.2%). Most studies focused on men (84.9%), followed by both men and women (9.3%). The majority of study populations consisted of adults (85.9%), with 10.9% including both adults and youth, and only 3.0% specifically targeting juveniles. The offender types studied were mainly sex offenders (31.6%) or violent offenders (21.6%), though offender type was unspecified in over a third of the studies. Regarding diagnoses, more than half of the studies (51.7%) investigated individuals with (juvenile) psychopathy (or traits), antisocial personality disorder, conduct disorder, and/or disruptive behavior disorder. Additionally, 21.8% focused on sexual disorders (particularly paedophilia). Disorders such as substance use disorders (3.2%) and psychotic disorders, including schizophrenia (2.1%), were far less represented. In 75.9% of the studies, one or more control groups were included. Over half of these compared the study population to healthy controls or non-offenders, while a third used other offender types. In 14.4%, the control groups consisted of psychiatric patients.

**Table 1:** Characteristics of study, study population, and (if applicable) control group(s).

Study type	431	100%
Observational	412	95.6%
*Case-control*	270	62.6%
*Cross-sectional*	104	24.1%
*Cohort*	23	5.3%
*Case-study, case-series*	14	3.2%
*Cross-sectional + cohort*	1	0.2%
Experimental	11	2.6%
Quasi-experimental	8	1.9%
Follow-up duration		
*Not applicable*	378	87.7%
*< 1 year (range 25 minutes - 1 year)*	35	8.1%
*≥ 1 year (range 1 year - 21 years)*	18	4.2%
Context in which the study was performed	482	100%
*Correctional facility*	220	45.6%
*Inpatient clinic*	165	34.2%
*Outpatient clinic*	78	16.2%
*Other (e.g., community)*	6	1.2%
*Not specified*	13	2.7%
Characteristics of study population		
Gender	431	100%
*Male*	366	84.9%
*Female*	14	3.2%
*Male and female*	40	9.3%
*Not specified*	11	2.6%
Age	431	100%
*Adult(s)*	370	85.8%
Juvenile(s)*	13	3.0%
*Adults and juveniles*	47	10.9%
*Not specified*	1	0.2%
Offender type	431	100%
*Violent offender*	93	21.6%
*Sex offender*	136	31.6%
*Mixed offender group*	38	8.8%
*Other*	5	1.2%
*Not specified*	159	36.9%
Diagnosis	431	100%
*(Juvenile) psychopathy (traits), ASPD, CD, disruptive behavior disorder*	223	51.7%
*Paraphilia, paedophilia, exhibitionism*	94	21.8%
*Schizophrenia, psychotic disorder*	9	2.1%
*Substance Use Disorder*	14	3.2%
*Other (e.g., personality disorder, ADHD, …)*	24	5.6%
*Mixed diagnoses*	40	9.3%
*Not specified*	27	6.3%
Characteristics of control group(s)†	327‡	100%
*Healthy controls/non-offenders*	170	52.0%
*Offenders*	109	33.3%
*Psychiatric patients*	47	14.4%
*Not specified*	1	0.3%

*Study populations are classified as ‘juveniles’ if specifically stated in the study; upper age limits vary up to a maximum of 21 years depending on the country where the study was conducted; † in 139 of the 431 included studies there was no control group; ‡ in a number of studies there was more than one control group; these are only counted separately here if they differ in terms of characterization.

**Table 2** provides an overview of cumulative data on biomarker outcome measures and functions extracted from the included studies. In just over half of the studies, brain activity (central nervous system) was examined as a biomarker outcome. Peripheral sympathetic arousal (e.g., skin conductance, heart rate variability) was assessed in 29,2% of the studies, and peripheral sexual arousal was studied in 13,8%. Relatively few studies examined eye movement, measured with eye trackers, or other biomarker outcomes. Regarding the functions of biomarkers, etiology (examining underlying substrates of forensic-relevant psychopathology and/or delinquent behavior) was clearly the main focus (77.2%). Biomarkers with diagnostic functions followed at an appropriate distance (12.7%), while other biomarker functions (monitoring, intervention, prognostic, and predictive biomarkers) were rarely investigated.

**Table 2:** Biomarker outcome measure and function.

Biomarker outcome measure	544*	100%
Central nervous system: brain activity	279	51.3%
Peripheral sympathetic arousal	159	29.2%
*Skin conductance/resistance (level)*	57	10.5%
*Heart rate (variability)*	46	8.5%
*Startle response*	19	3.5%
*(Facial) electromyographic (muscle) activity*	17	3.1%
*Respiration rate*	9	1.7%
*Temperature*	5	0.9%
*Blood pressure (diastolic and/or systolic)*	4	0.7%
*Pupil dilatation*	2	0.4%
Peripheral sexual arousal†	75	13.8%
*Change in penis circumference*	74	13.6%
*Vaginal blood volume, vaginal pulse rate and amplitude, response duration*	1	0.2%
Eye movement (eye tracker)	13	2.4%
Other	19	3.5%
*Eye movement (EOG)*‡	12	2.2%
*Movement*	3	0.6%
*Physiological indicators not specified*§	3	0.6%
*Speech reactions*	1	0.2%
Biomarker function¶	434	100%
*Etiologic *	335	77.2%
*Diagnostic*	55	12.7%
*Monitoring*	19	4.4%
*Intervention*	14	3.2%
*Prognostic‖*	8	1.8%
Predictive**	3	0.7%

*The total number of biomarker outcome measures exceeds the amount of studies (n = 431) because in several studies more than one biomarker outcome measure was used; † although ‘peripheral sexual arousal’ is also a sympathetic nervous system activity, this biomarker is categorized separately here because of its specific association with sex offenders; ‡ eye movement as measured with EOG, other than eye movement as measured with an eye tracker, is shown here in the ‘other’ category because it only serves the purpose of correcting for eyeblinks or detection of artifacts when interpreting an EEG; § the studies in question do not specify which physiological biomarkers, usually measured with a polygraph, were involved; ¶ the total number of biomarker functions exceeds the amount of studies because in three studies there were two biomarker functions; ‖ in this study, ‘prognostic’ function represents biomarkers that are used to estimate the likelihood of crime recidivism; ** the ‘predictive’ function here refers to biomarkers that are used to predict which patients benefit from certain interventions.

**Table 3** lists the biomarker assessment methods used in the included studies. fMRI (31.3%) and EEG (25.5%) were the most common methods, both of which measure brain activity. PPG (penile plethysmography), used to measure peripheral sexual arousal, came in third place (15.6%). Not included in **Table 3** but noteworthy is that of the 75 included studies using PPG as an assessment method, 45 (60%) were conducted in Canada, 23 (30.7%) in the US, three (4.0%) in Western Europe, three (4%) in Eastern Europe and one (1.3%) in New Zealand. For non-sexual peripheral sympathetic arousal, a variety of methods and devices were used, including polygraph, skin conductance devices, EMG, ECG, and others. The use of wearables was minimal (1.9%). Finally, eye trackers or active electrodes for measuring oculomotor responses were rarely used, consistent with earlier findings regarding biomarker outcomes. **Table 3** also shows the stimuli or tasks used to elicit participant responses, applicable in 357 studies (82.8%). These stimuli and tasks were diverse (outlined in **Supplementary Table 1**) and **Table 3** provides an outline classification for this purpose. Emotion tasks, sexual stimuli, and cognitive tasks were used approximately equally, together accounting for 79.8% of the total.

**Table 3:** Biomarker assessment method and stimulus.

Biomarker assessment method	482*	100%
Central nervous system (brain) activity assessment	279	57.9%
*fMRI*	151	31.3%
*EEG (including ERP, QEEG [quantitative EEG])*	123	25.5%
*SPECT*	4	0.8%
*MEG (magnetoencephalography)*	1	0.2%
Peripheral sympathetic arousal assessment	103	21.4%
*Polygraph (divers types)*†	33	6.8%
*SCD (skin conductance device)*	25	5.2%
*EMG*	18	3.7%
*ECG (EKG), photo-plethysmograph, photoelectric cell and tachometer, digital pulse oximeter (for heart rate)*	13	2.7%
*Wearable: VU-AMS, Sense-IT, Empatica E4, Polar WearLink, Actigraph*	9	1.9%
*Servo system (systolic blood pressure), Omron M5-I (systolic and diastolic blood pressure)*	2	0.4%
*Thermometer (tympanic, digit)*	2	0.4%
*Video-recording pupil dilatation*	1	0.2%
Peripheral sexual arousal assessment‡	75	15.6%
*PPG (penis plethysmography)*	74	15.4%
*Vaginal photoplethysmography*	1	0.2%
Oculo-motor reaction assessment: eye-tracker, active electrodes	13	2.7%
Other	12	2.5%
*EOG*§	12	2.5%
Stimulus¶	357^‖^	100%
*Emotion task (e.g., emotion recognition, emotion processing, fear conditioning) *	99	27.7%
*Sexual stimuli (audio and/or video, pictures)*	98	27.5%
*Cognitive task (e.g., selective attention, response inhibition, cognitive flexibility)*	88	24.6%
*Moral/empathy task*	19	5.3%
*Perceptual-motor task (e.g., playing a video game)*	6	1.7%
*Neurofeedback*	5	1.4%
*Drug cue exposure task*	4	1.1%
*Drug administered*	2	0.6%
*Transcranial magnetic stimulation*	1	0.3%
*Other*	33	9.2%

*The total number of biomarker outcome assessment methods exceeds the amount of studies because in several studies more than one biomarker assessment method was used; † not restricted to ‘lie detection’; ‡ although ‘peripheral sexual arousal assessment’ is also a peripheral sympathetic arousal assessment, this assessment method is categorized separately here because of its specific association with sex offenders; § EOG is shown here in the ‘other’ category because it only serves the purpose of correcting for eyeblinks or detection of artifacts when interpreting an EEG; ¶ in 79 of the 431 included studies no stimulus was used (e.g., rest-state fMRI/EEG); ‖ in a number of studies more than one stimulus was used; these are only counted separately here if they differ in terms of characterization.

**Figure 4:**
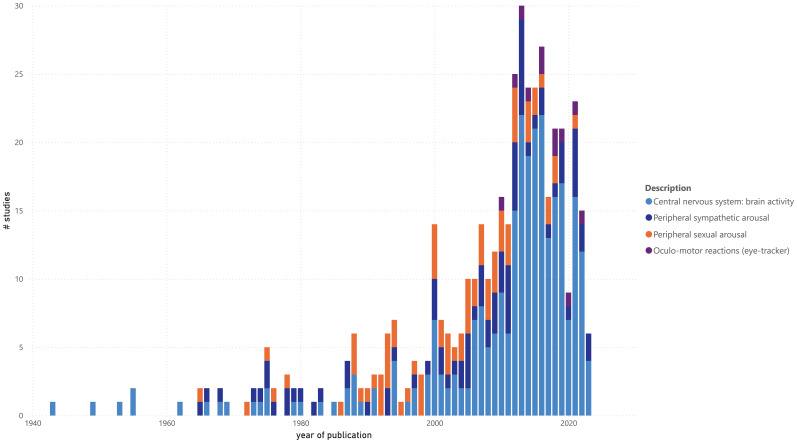
Number of studies by year of publication, differentiated by biomarker outcome.

**Figure 5:**
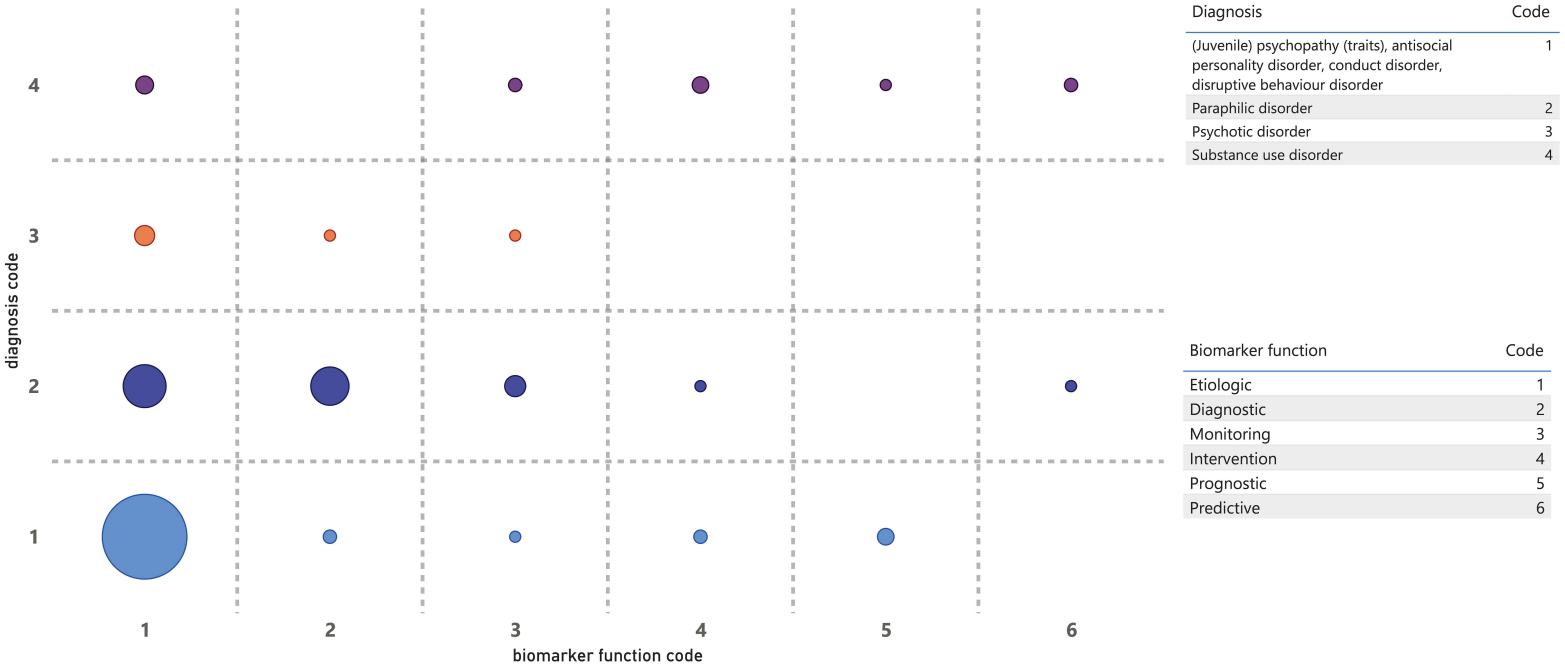
Diagnosis by biomarker function.

**Figure 6:**
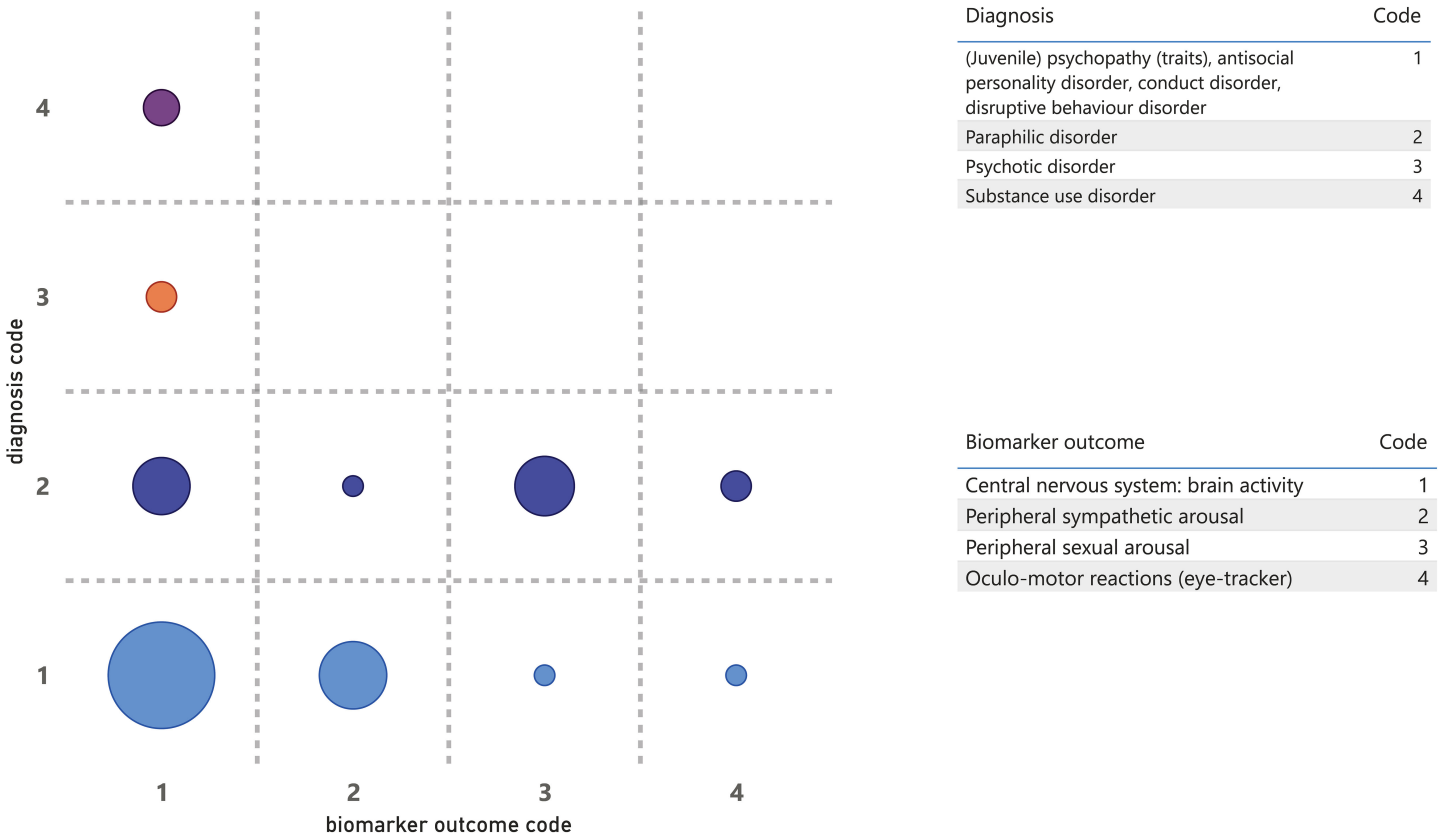
Diagnosis by biomarker outcome.

Finally, in the context of this scoping review, the association between some of the previously presented variables is visualized in **Figures 4**–**6**. **Figure 4** shows the number of studies per year of publication, similar to **Figure 2**, but now differentiated by biomarker outcome. This again shows the predominance and increasing trend of research into brain activity and/or activation. However, it also indicates that PPG as an assessment method may be declining in recent years and that assessment methods for non-sexual peripheral arousal and oculomotor responses, albeit still limited, seem to be emerging.

**Figure 5** visualizes the diagnosis by biomarker function. This gap map shows that most etiological research with physiological biomarkers has been conducted in forensic patients with psychopathy, antisocial personality disorder, and paraphilias. For paraphilias, a reasonable portion of the research also addressed diagnostic biomarkers and, to a lesser extent, monitoring. The gaps, both partial and complete, were identified, particularly in patients with psychotic and substance use disorders. Additionally, significant gaps persist across all patient categories regarding biomarker functions monitoring, intervention, prognostic, and predictive purposes.

Last, **Figure 6** visualizes diagnosis by biomarker outcome. This gap map shows that the scarce research in forensic patients with psychotic or substance use disorders primarily focused on the biomarker outcome of brain activity. Furthermore, it emerges that relatively little research is done in individuals with sexual disorders using biomarker outcomes that monitor non-sexual peripheral sympathetic activity, and that research with oculomotor outcomes is limited or absent across all diagnosis groups.

## 4 Discussion

The objective of this scoping review was to investigate the extent (size), range (variety), and nature (characteristics) of the evidence on the use of physiological biomarkers in forensic psychiatry. The review showed that the number of studies meeting the inclusion criteria has increased exponentially over recent decades, consistent with general trends in modern scientific research (446). However, a slight decline in the focus on physiological biomarkers in forensic psychiatry was observed in recent years (**Figure 2**). Whether this decline represents a sustained trend or merely a temporary post-COVID-19 publication dip remains to be seen. The majority of the 431 studies included were conducted in North America. This is not surprising as the US has long been a leading contributor to scientific output (447), including studies in psychiatry (439). Other countries in the top six, such as Canada, were also expected, given Canada’s, pioneering developments in forensic psychiatry, including the Risk-Need-Responsivity (RNR) model (448), the Psychopathy Checklist (449), and several risk assessment instruments (450).

A significant finding is that over 95% of the included studies had an observational design, highlighting a gap in research on the use of physiological biomarkers in forensic psychiatry. This becomes even more clearer when considering the variable ‘biomarker function’. Most research focused on the etiological function, examining underlying substrates of associations between psychopathology and delinquent behavior. Although this knowledge contributes to the scientific foundation, much of the research appears repetitive. Also, the clinical relevance of such studies is questionable. Forensic care primarily aims to prevent crime recidivism, which requires robust assessment tools, effective interventions, reliable response predictions, robust effect monitoring, and improved recidivism risk estimation. Thus, these findings indicate a big discrepancy between the current state of the literature and the practical needs of the clinical field. To meet these needs, more (experimental) intervention studies should be conducted, along with (observational) studies involving monitoring, predictive, and prognostic physiological biomarkers. These studies should preferably adopt longitudinal designs with multiple or continuous measurements. However, as shown in this scoping review, such studies are only marginally represented. The preference for observational, non-longitudinal designs likely stems from their fewer methodological and logistical challenges compared to longitudinal experimental research. Nonetheless, it is now clear that longitudinal and intervention-focused research requires far more attention moving forward.

Most included studies were conducted in correctional facilities, often in prison settings, or forensic inpatient clinics accounting for 79.8% of the total. This dominance may be related to the logistical and substantive advantages of these settings, such as the availability of participants, better control over external factors, and/or extrinsic motivators for participation. The dominance of inpatient studies also aligns with the RNR model, emphasizing efforts on forensic patients who commit serious crimes and who pose the highest security risk to society. These patients are often treated in inpatient settings (451).

Another notable gap arises when examining research populations. The sex ratio in the studies reflects the male-dominated forensic populations, with 82-95% males in European forensic settings (452) and similar figures for violence offenders globally (453). However, only a small number of studies include minors. Forensic child and adolescent psychiatry is a relatively young and niche field in most countries (454). Nevertheless, early interventions in juvenile delinquency are crucial, as such behavior often precedes adult criminal behavior (455). Thus, the lack of studies focusing on minors represents a significant gap that needs to be addressed. Next, there is limited research on certain psychiatric diagnoses among participants. Almost three-quarters of the studies focused on individuals with (juvenile) psychopathy, antisocial personality disorder, and paraphilias, while participants with psychotic or substance use disorders comprised only 5.3%. This is striking, given the overrepresentation of these disorders in forensic populations and their strong association with crime recidivism (456–458).

Regarding biomarker outcomes and assessment methods, brain activity and activation, along with their associated measurement tools, have dominated the field for years. Researchers often prioritize studying the brain, the ‘command center of body and mind’, to explain forensic abnormalities, despite the limitations and challenges of brain research in clinical practice. The focus may be linked to an overreliance on observational, mainly non-longitudinal research. Recently, the measurement of peripheral sympathetic arousal has gained traction due to its practicality, suitability for multiple or continuous measurements, and potential use in patient self-monitoring for impulse control. However, newer assessment methods, such as wearables, eye-tracking systems, and voice stress analysis tools, remain underutilized. Their cautious yet increasing adoption suggests a promising trend toward addressing this gap in forensic research. Finally, the near-exclusive use of PPG in North America, and its apparent decline, reflects ethical and legal obstacles, particularly regarding the sexual stimuli used. These constraints are notably stricter in Europe.

This scoping review has several strengths, including being the first of its kind and being conducted systematically using the JBI methodology for scoping reviews. Additionally, the research question and methodology were predefined in a protocol registered on the Open Science Framework (https://osf.io/46qbu/). Any changes to the original protocol are documented in this paper, which facilitates the critical appraisal of the methodology and helps in the identification of potential issues such as reporting bias (459). Furthermore, data extraction was conducted on 431 studies, highlighting another strength of this study: the substantial body of evidence on which the results are based. A possible limitation is the inclusion of only published studies reporting primary (original) research. Despite this, it is unlikely that a broader search would have led to different results due to saturation in the existing literature. Following the guidelines for scoping reviews (15), we aimed to provide a broad overview of the available literature and to identify possible gaps, rather than to conduct an in-depth evaluation, such as a quality assessment of the included studies and the risk of bias, as is typically done in systematic reviews. However, by limiting the scope to studies published in scientific journals and dissertations only, we can assume that these studies have already undergone a review process, which can be considered a certain guarantee of their quality.

In conclusion, this scoping review provides insight into the use of physiological biomarkers in forensic psychiatry and highlights several gaps in the scientific literature. In particular, there is a need for more longitudinal research to investigate the integration of physiological biomarkers into interventions, effect monitoring, crime risk prognostication, and prediction of patient responsiveness. Also, more attention is needed on juveniles, patients with psychotic and substance use disorders, and investigating new biomarker assessment methods, particularly those measuring peripheral arousal.

## Data Availability

The original contributions presented in the study are included in the article/**Supplementary Material**. Further inquiries can be directed to the corresponding author.

## Supplementary material

Supplementary Table 1 Study details and characteristics. NA, Not Applicable; NS, Not Specified; PPG, Penile plethysmography.
