# Supplementary material for: Using physiological biomarkers in forensic psychiatry: a scoping review
Source: Front Psychiatry. 2025 Apr 29;16:1580615. doi: 10.3389/fpsyt.2025.1580615 (PMC12069285; doi:10.3389/fpsyt.2025.1580615)
Supplement: Supplementary Table 1 — Study details and characteristics. NA, Not Applicable; NS, Not Specified; PPG, Penile plethysmography. [file Table1.docx]

|  | Study Objective | General study characteristics | Participants/controls | Biomarker characteristics | Main study conclusion |
| --- | --- | --- | --- | --- | --- |
| Abe et al. (2018) | To test whether psychopathic individuals show reduced activity in the anterior cingulate cortex when confronted with an opportunity for dishonest gain | *Design:* Case-control study  *Country:* USA  *Setting:* Correctional facility  *Follow-up:* NA | *N:* 18  *Gender:* M  *Age category:* Adult  *Diagnosis:* Psychopathy  *Offender type:* NS  *Controls:* M (17): Medium psychopathy group), M (32): Low psychopathy group) | *Outcome measure:* Brain activity  *Assessment method:* fMRI  *Function:* Etiologic  *Stimulus/task:* Coin-flip prediction task | Psychopathic individuals behave dishonestly with relatively low levels of response conflict and the anterior cingulate gyrus may play a critical role in this pattern of behaviour |
| Abel et al. (1998) | To evaluate reliability and validity of visual reaction time and PPG in groups of individuals with sexual interest in children of various ages and genders | *Design:* Case-control study  *Country:* USA  *Setting:* Outpatient clinic  *Follow-up:* NA | *N:* 157  *Gender:* M  *Age category:* Adult and juvenile  *Diagnosis:* NS  *Offender type:* Sex offender: child victim  *Controls:* M (NS): Other offenders | *Outcome measure:* Change in penis circumference  *Assessment method:* PPG  *Function:* Etiologic  *Stimulus/task:* Slides of (non-)sexual stimuli (different ages/genders) | Both visual reaction time and PPG demonstrated good reliability for measuring sexual interest |
| Aghajani et al. (2016) | To examine the intrinsic functional connectivity of basolateral and centromedial amygdala networks to affective, interpersonal, and behavioural traits of psychopathy in CD juveniles with a history of delinquency | *Design:* Cross-sectional study  *Country:* The Netherlands  *Setting:* Correctional facility and inpatient clinic  *Follow-up:* NA | *N:* 50  *Gender:* M  *Age category:* Juvenile  *Diagnosis:* Conduct disorder  *Offender type:* Violent offenders  *Controls:* NA | *Outcome measure:* Brain activity  *Assessment method:* fMRI  *Function:* Etiologic  *Stimulus/task:* NA (resting-state fMRI) | Amygdala connectivity profiles exhibited dissociable relations with different traits of psychopathy |
| Aghajani et al. (2021) | To examine neural processing of recognizing and resonating negative socio-emotional content (angry and fearful faces) among CD offenders with LPE, relative to CD offenders without LPE and healthy controls | *Design:* Case-control study  *Country:* The Netherlands  *Setting:* Correctional facility and inpatient clinic  *Follow-up:* NA | *N:* 50  *Gender:* M  *Age category:* Juvenile  *Diagnosis:* Conduct disorder  *Offender type:* Violent offenders  *Controls:* M (31): Healthy controls | *Outcome measure:* Brain activity  *Assessment method:* fMRI  *Function:* Etiologic  *Stimulus/task:* Emotional processing task *(distressing facial expressions)* | CD/LPE+ youths may exhibit an over-reliance on cortical neurocognitive systems when explicitly processing negative socioemotional information, which could have adverse downstream effects on relevant socioemotional functions |
| Aghajani et al. (2017) | To examine intrinsic functional connectivity of basolateral and centromedial amygdala networks and whether perturbed amygdala subregional connectivity coincides with altered volume and shape of the amygdaloid complex across CD juvenile offenders with and without CU traits and matched healthy controls | *Design:* Case-control study  *Country:* The Netherlands  *Setting:* Correctional facility and inpatient clinic  *Follow-up:* NA | *N:* 50  *Gender:* M  *Age category:* Juvenile  *Diagnosis:* Conduct disorder  *Offender type:* Violent offenders  *Controls:* M (24): Healthy controls | *Outcome measure:* Brain activity  *Assessment method:* fMRI  *Function:* Etiologic  *Stimulus/task:* NA (resting-state fMRI) | The connectivity changes coincided with local hypotrophy of BLA and CMA subregions (without being statistically correlated) and were associated to more severe CU symptoms |
| Allen et al. (2022) | To examine whether error monitoring activity in the dACC, as well as psychopathy scores and age at release, predicted non-violent felony rearrest in women | *Design:* Cohort study  *Country:* USA  *Setting:* Correctional facility  *Follow-up:* 2 to 12 years | *N:* 248  *Gender:* F  *Age category:* Adult  *Diagnosis:* Psychopathy  *Offender type:* NS  *Controls:* NA | *Outcome measure:* Brain activity  *Assessment method:* fMRI  *Function:* Prognostic  *Stimulus/task:* Go-NoGo task | Error monitoring activity in dACC, as well as psychopathy scores and age at release predicted non-violent felony rearrest in women |
| Allen et al. (2022) | To examine whether psychopathic traits were associated with aberrant internetwork connectivity, intranetwork connectivity, and amplitude of fluctuations across limbic and surrounding paralimbic regions among incarcerated women | *Design:* Cross-sectional study  *Country:* USA  *Setting:* Correctional facility  *Follow-up:* NA | *N:* 297  *Gender:* F  *Age category:* Adult  *Diagnosis:* Psychopathy  *Offender type:* NS  *Controls:* NA | *Outcome measure:* Brain activity  *Assessment method:* fMRI  *Function:* Etiologic  *Stimulus/task:* NA (resting-state fMRI) | Psychopathic traits among incarcerated women are associated with aberrant intra-network amplitude fluctuations and connectivity across multiple networks including limbic and surrounding paralimbic regions |
| Allen et al. (2023) | To examine whether psychopathic traits were associated with altered internetwork connectivity, intranetwork connectivity, and amplitude of low-frequency fluctuations across resting-state networks among high-risk incarcerated adolescent girls | *Design:* Cross-sectional study  *Country:* USA  *Setting:* Correctional facility  *Follow-up:* NA | *N:* 40  *Gender:* F  *Age category:* Juvenile  *Diagnosis:* Psychopathy  *Offender type:* NS  *Controls:* NA | *Outcome measure:* Brain activity  *Assessment method:* fMRI  *Function:* Etiologic  *Stimulus/task:* NA (resting-state fMRI) | Psychopathic traits among incarcerated adolescent girls are associated with altered intra-network ALFFs, primarily that of increased low-frequency and decreased high-frequency fluctuations, and connectivity across multiple networks including paralimbic regions |
| Anderson et al. (2018) | To examine neural activity using fMRI during a simple auditory target detection (oddball) task among incarcerated adult males with psychopathic traits | *Design:* Cross-sectional study  *Country:* USA  *Setting:* Correctional facility  *Follow-up:* NA | *N:* 168  *Gender:* M  *Age category:* Adult  *Diagnosis:* Psychopathy  *Offender type:* NS  *Controls:* NA | *Outcome measure:* Brain activity  *Assessment method:* fMRI  *Function:* Etiologic  *Stimulus/task:* Auditory oddball task | Results support models of psychopathy describing impaired integration across functional networks |
| Anderson et al. (2015) | To examine psychopathy-related differences in ERPs during an auditory oddball task in a sample of incarcerated adult males | *Design:* Cross-sectional study  *Country:* USA  *Setting:* Correctional facility  *Follow-up:* NA | *N:* 59  *Gender:* M  *Age category:* Adult  *Diagnosis:* Psychopathy  *Offender type:* NS  *Controls:* NA | *Outcome measure:* Brain activity  *Assessment method:* EEG  *Function:* Etiologic  *Stimulus/task:* Auditory oddball task | Features of psychopathy were associated with increased target N1 amplitude (facets 1, 4), decreased target P3 amplitude (facet 1), and reduced slow wave amplitude for frequent standard stimuli (facets 1, 3, 4) |
| Anderson et al. (2017) | To examine attention–emotion interactions using functional neuroimaging among incarcerated individuals evaluated for psychopathic traits | *Design:* Cross-sectional study  *Country:* USA  *Setting:* Correctional facility  *Follow-up:* NA | *N:* 120  *Gender:* M  *Age category:* Adult  *Diagnosis:* Psychopathy  *Offender type:* NS  *Controls:* NA | *Outcome measure:* Brain activity  *Assessment method:* fMRI  *Function:* Etiologic  *Stimulus/task:* Emotion/attention task (emotionally evocative or non-emotional stimuli) | The affective-interpersonal features of psychopathy were associated with relatively lower emotion-dependent augmentation of activity in visual processing areas during implicit emotional processing, while antisocial-lifestyle features were associated with elevated activity in the amygdala and related salience network regions. During explicit emotional processing, psychopathic traits were associated with upregulation in the medial prefrontal cortex, insula, and superior frontal regions |
| Anton et al. (2012) | To characterize and differentiate between dysfunctional cognitive and affective processes associated with attentional focus and cognitive load using a fear-conditioning paradigm in a sample of incarcerated female offenders | *Design:* Cross-sectional study  *Country:* USA  *Setting:* Correctional facility  *Follow-up:* NA | *N:* 84  *Gender:* F  *Age category:* Adult  *Diagnosis:* Psychopathy  *Offender type:* NS  *Controls:* NA | *Outcome measure:* ERP, startle eyeblink electromyographic activity  *Assessment method:* EEG, EMG  *Function:* Etiologic  *Stimulus/task:* Fear-conditioning task | Simultaneous analysis of psychopathy and APD revealed distinct patterns of cognitive processing and fear reactivity |
| Arnett et al. (1993) | To examine whether, in comparison to heart rate following reward, psychopaths would display greater heart rate following punishment than non-psychopaths | *Design:* Case-control study  *Country:* USA  *Setting:* Correctional facility  *Follow-up:* NA | *N:* 29  *Gender:* M  *Age category:* Adult  *Diagnosis:* Psychopathy  *Offender type:* NS  *Controls:* M (28): Offenders, low psychopathy | *Outcome measure:* Skin conductance, heart rate  *Assessment method:* SCD, ECG/photo-plethysmograph (HR)  *Function:* Etiologic  *Stimulus/task:* Successive Go-NoGo discrimination with four reward stimuli and four punishment stimuli | Psychopaths displayed lower HR and fewer SCRs than controls following punishment. This indicates that psychopaths were relatively unresponsive to punishment and feedback in this study |
| Baker (1985) | To examine neuropsychological and power spectral EEG characteristics of exhibitionists | *Design:* Case-control study  *Country:* Canada  *Setting:* Outpatient clinic  *Follow-up:* NA | *N:* 23  *Gender:* M  *Age category:* Adult  *Diagnosis:* Exhibitionistic disorder  *Offender type:* Sex offenders: Victim not specified  *Controls:* M (19): Healthy controls | *Outcome measure:* Brain activity  *Assessment method:* EEG  *Function:* Etiologic  *Stimulus/task:* Eyes open and eyes closed rest tasks, vocabulary and word fluency tasks, spatial task (block design) | Frontal asymmetry (higher alpha activity left) was higher in exhibitionists than in controls during rest |
| Barrat et al. (1997) | To examine whether subjects who commit impulsive versus non-impulsive aggression differ on measurements of personality, neuropsychology, and cognitive psychophysiology, and whether these differences can yield information regarding the etiology of impulsive aggression | *Design:* Case-control study  *Country:* USA  *Setting:* Correctional facility  *Follow-up:* NA | *N:* 57  *Gender:* M  *Age category:* Adult  *Diagnosis:* Antisocial personality disorder  *Offender type:* Violent offenders  *Controls:* M (44): Non-offenders | *Outcome measure:* ERP  *Assessment method:* EEG  *Function:* Etiologic  *Stimulus/task:* Two-choice response tasks | Impulsiveness, anger, and peak P300 latencies did not differ between the inmate groups but verbal symbol decoding and peak P300 amplitudes did |
| Baskin-Sommers et al. (2012) | To examine attentional abnormalities that undermine the processing of secondary affective stimuli in psychopathy and its associated factors | *Design:* Cross-sectional study  *Country:* USA  *Setting:* Correctional facility  *Follow-up:* NA | *N:* 87  *Gender:* M  *Age category:* Adult  *Diagnosis:* Psychopathy/psychopathic traits  *Offender type:* Violent offenders  *Controls:* NA | *Outcome measure:* Fear potentiated startle  *Assessment method:* EMG  *Function:* Etiologic  *Stimulus/task:* Fear conditioning task with electric shocks | Results are indicative for attention to moderate fearlessness of psychopathic individuals and for an early attention bottleneck as a proximal mechanism for deficient response modulation in psychopathy |
| Baskin-Sommers et al. (2011) | To examine the generalizability of psychopathy-related fear deficits in African American offenders | *Design:* Cross-sectional study  *Country:* USA  *Setting:* Correctional facility  *Follow-up:* NA | *N:* 92  *Gender:* M  *Age category:* Adult  Psychopathy/psychopathic traits  *Offender type:* NS  *Controls:* NA | *Outcome measure:* Fear potentiated startle  *Assessment method:* EMG  *Function:* Etiologic  *Stimulus/task:* Fear conditioning with noise probes | There is no evidence that psychopathy relates to fear deficits in African American offenders |
| Baskin-Sommers et al. (2011) | To evaluate whether poorly regulated behaviour of psychopathic individuals reflects a problem in reallocating attention to process peripheral information while engaged in goal-directed behaviour using male prisoners | *Design:* Cross-sectional study  *Country:* USA  *Setting:* Correctional facility  *Follow-up:* NA | *N:* 101  *Gender:* M  *Age category:* Adult  *Diagnosis:* Psychopathy  *Offender type:* Mixed offender types  *Controls:* NA | *Outcome measure:* ERP  *Assessment method:* EEG  *Function:* Etiologic  *Stimulus/task:* Alternative-focus versus threat-focus conditions task | Results suggest that psychopathic individuals find it easier to ignore threat-related distractors when they are peripheral versus central to their goal-directed behaviour |
| Baskin-Sommers et al. (2013) | To evaluate potential effects of a putative attention bottleneck on the emotion processing of psychopathic offenders during picture viewing by manipulating picture familiarity and examining emotion-modulated startle and late positive potential | *Design:* Cross-sectional study  *Country:* USA  *Setting:* Correctional facility  *Follow-up:* NA | *N:* 136  *Gender:* M  *Age category:* Adult  *Diagnosis:* Psychopathy  *Offender type:* NS  *Controls:* NA | *Outcome measure:* ERP, startle response  *Assessment method:* EEG, ERP  *Function:* Etiologic  *Stimulus/task:* Picture viewing task (emotional processing) | Psychopathic individuals displayed the classic deficit in emotion-modulated startle during novel pictures but they showed no deficit in emotion-modulated startle during familiar pictures. Results for LPP responses revealed psychopathy-related differences during familiar pictures and no psychopathy-related differences during novel pictures |
| Becker et al. (1992) | To evaluate test-retest reliability in two groups of adolescent sex offenders using audiotaped stimuli presented during a 24-hour interval | *Design:* Cohort study  *Country:* USA  *Setting:* Inpatient clinic and outpatient clinic  *Follow-up:* 1 week | *N:* 20  *Gender:* M  *Age category:* Juvenile  *Diagnosis:* Not explicitly stated  *Offender type:* Sex offenders: Child victims  *Controls:* NA | *Outcome measure:* Change in penis circumference  *Assessment method:* PPG  *Function:* Monitoring  *Stimulus/task:* Audiotaped cues (verbal portrayals of sexual interactions) | Test-retest reliability was demonstrated for 15 of the 19 audiotaped vignettes. The highest correlations were found for those sexual behaviours in which the adolescents had engaged |
| Becker et al. (1988) | To describe a community-based outpatient treatment program for male adolescent sexual offenders and therapy outcome for those adolescents who completed treatment and posttreatment evaluation | *Design:* Cohort study  *Country:* USA  *Setting:* Outpatient clinic  *Follow-up:* 1 week | *N:* 24  *Gender:* M  *Age category:* Juvenile  *Diagnosis:* Not explicitly stated  *Offender type:* Sex offenders: Child and adult victims  *Controls:* NA | *Outcome measure:* Change in penis circumference  *Assessment method:* PPG  *Function:* Monitoring  *Stimulus/task:* Audiotaped cues (verbal portrayals of sexual interactions) | For subjects who were involved with male victims the treatment was successful in assisting them to reduce their arousal to deviant sexual stimuli. This was not demonstrated statistically for subjects who were involved with female victims |
| Becker et al. (1992) | To examine the relationship between erection profiles, admission of guilt, and the offenders' own victimization | *Design:* Cross-sectional study  *Country:* USA  *Setting:* Outpatient clinic  *Follow-up:* NA | *N:* 83  *Gender:* M  *Age category:* Juvenile  *Diagnosis:* Not explicitly stated  *Offender type:* Sex offenders: Child and adult victims  *Controls:* NA | *Outcome measure:* Change in penis circumference  *Assessment method:* PPG  *Function:* Diagnostic  *Stimulus/task:* Audiotaped cues (verbal portrayals of sexual interactions) | Given that 58% of the juveniles who denied their sex offense were non-responders, plethysmography is of limited use diagnostically with this subgroup, since it does not differentiate between cues |
| Becker et al. (1992) | To evaluate adolescent sexual offenders by penile plethysmography using two-minute audiotaped stimulus cues to see whether they are suitable subjects for psychophysiologic assessment | *Design:* Cross-sectional study  *Country:* USA  *Setting:* Outpatient clinic  *Follow-up:* NA | *N:* 129  *Gender:* M  *Age category:* Juvenile  *Diagnosis:* Not explicitly stated  *Offender type:* Sex offenders: Child and adult victims  *Controls:* NA | *Outcome measure:* Change in penis circumference  *Assessment method:* PPG  *Function:* Diagnostic  *Stimulus/task:* Audiotaped cues (verbal portrayals of sexual interactions) | Adolescent males are suitable subjects for psychophysiologic assessment which can be used as a part of overall evaluation to determine the treatment needs in this population |
| Birbaumer et al. (2005) | To examine cerebral, peripheral, and subjective correlates of fear conditioning in criminal psychopaths and healthy control subjects | *Design:* Case-control study  *Country:* Germany  *Setting:* Correctional facility  *Follow-up:* NA | *N:* 10  *Gender:* M  *Age category:* Adult  *Diagnosis:* Psychopathy  *Offender type:* NS  *Controls:* M (10): Healthy controls | *Outcome measure:* Brain activity, electrodermal responses  *Assessment method:* fMRI, SCD  *Function:* Etiologic  *Stimulus/task:* Aversive differential Pavlovian delay conditioning paradigm with slides of neutral faces serving as conditioned and painful pressure as unconditioned stimuli | The dissociation of emotional and cognitive processing may be the neural basis of the lack of anticipation of aversive events in criminal psychopaths |
| Blackburn (1975) | To examine whether aggressive offenders have a greater amount of theta activity in their electrocortical rhythms than non-aggressive offenders and whether such individuals are cortically under-aroused, under-reactive, or more easily de-aroused | *Design:* Cross-sectional study  *Country:* UK  *Setting:* Inpatient clinic  *Follow-up:* NA | *N:* 80  *Gender:* M  *Age category:* Adult  *Diagnosis:* Personality disorder  *Offender type:* Mixed offender types  *Controls:* NA | *Outcome measure:* Brain activity  *Assessment method:* EEG  *Function:* Etiologic  *Stimulus/task:* Repetitive auditory stimulation and cold pressor test | More aggressive subjects tended to have a higher dominant frequency at rest, less increase in theta during monotonous stimulation, and greater alpha reactivity to cold pressor stimulation |
| Blackburn (1979) | To establish the generality of the hypothesis that psychopaths are cortically and autonomically under-aroused and under-reactive and to determine whether primary and secondary psychopaths are similar in this respect | *Design:* Cross-sectional study  *Country:* UK  *Setting:* Inpatient clinic  *Follow-up:* NA | *N:* 20  *Gender:* M  *Age category:* Adult  *Diagnosis:* Psychopathy  *Offender type:* Mixed offender types  *Controls:* M (20): Patients, non-psychopathic | *Outcome measure:* Electrocortical, electrodermal and cardiovascular activity  *Assessment method:* EEG, theratronics polygraph  *Function:* Etiologic  *Stimulus/task:* Repetitive auditory stimulation and cold pressor test | Psychopaths were not significantly differentiated from non-psychopaths, but several differences were found between primary and secondary psychopaths |
| Blair et al. (1997) | To investigate the psychophysiological responsiveness of psychopathic individuals to distress cues and threatening and neutral stimuli | *Design:* Case-control study  *Country:* UK  *Setting:* Correctional facility and inpatient clinic  *Follow-up:* NA | *N:* 18  *Gender:* M  *Age category:* Adult  *Diagnosis:* Psychopathy  *Offender type:* Violent offenders  *Controls:* M (18): Patients, non-psychopathic | *Outcome measure:* Skin conductance  *Assessment method:* SCD  *Function:* Etiologic  *Stimulus/task:* Distress cues, threatening and neutral stimuli | Psychopathic individuals showed (relative to controls) reduced electrodermal responses to distress cues. The two groups did not differ in electrodermal responses to threatening stimuli and neutral stimuli |
| Blanchard and Barbaree (2005) | To compare the rates of decline of sexual arousability in male paedophiles, hebephiles, and teleiophiles | *Design:* Cross-sectional study  *Country:* Canada  *Setting:* Inpatient clinic  *Follow-up:* NA | *N:* 2028  *Gender:* M  *Age category:* Adult  *Diagnosis:* Paedophilia  *Offender type:* Sex offenders: Child and adult victims  *Controls:* NA | *Outcome measure:* Change in penis circumference  *Assessment method:* PPG  *Function:* Etiologic  *Stimulus/task:* (Non-)sexual audio narratives and slides of (non-)sexual stimuli (different ages/genders) | Sexual arousability was an inverse function of age, and there were no differences between paedophiles, hebephiles, and teleiophiles in the rate at which arousability declined |
| Blanchard et al. (2002) | To investigate whether head injuries in childhood might increase the risk of paedophilia in males | *Design:* Case-control study  *Country:* Canada  *Setting:* Inpatient clinic  *Follow-up:* NA | *N:* 413  *Gender:* M  *Age category:* Adult  *Diagnosis:* Paedophilia  *Offender type:* Sex offenders: Child and adult victims  *Controls:* M (793): Patients, non-paedophilic | *Outcome measure:* Change in penis circumference  *Assessment method:* PPG  *Function:* Diagnostic  *Stimulus/task:* Film clips of nude adults or children smiling and walking slowly toward the camera and sexual audiotaped narratives | Paedophilia may be influenced by genetic factors, which are manifested in women as an increased risk of psychiatric problems, and in their sons, as an increased risk of erotic interest in children |
| Blanchard et al. (2001) | To calculate the specificity of phallometric testing for paedophilia using sex offenders against adult women and to examine whether the offender's actual number of prior sexual contacts with women affects such estimates | *Design:* Cross-sectional study  *Country:* Canada  *Setting:* Inpatient clinic  *Follow-up:* NA | *N:* 324  *Gender:* M  *Age category:* Adult  *Diagnosis:* Paedophilia  *Offender type:* Sex offenders: Child and adult victims  *Controls:* NA | *Outcome measure:* Change in penis circumference  *Assessment method:* PPG  *Function:* Diagnostic  *Stimulus/task:* Visual and auditory (erotic/sexual) depictions | The more adult women with whom a patient has had sexual contact, the less likely he is to be diagnosed as paedophilic |
| Blanchard et al. (2007) | To investigate whether the previously observed association of paedophilia with lower IQs is an artifact of heterogeneity in referral sources | *Design:* Cross-sectional study  *Country:* Canada  *Setting:* Inpatient clinic  *Follow-up:* NA | *N:* 832  *Gender:* M  *Age category:* Adult  *Diagnosis:* Paedophilia  *Offender type:* Sex offenders: Child and adult victims  *Controls:* NA | *Outcome measure:* Change in penis circumference  *Assessment method:* PPG  *Function:* Diagnostic  *Stimulus/task:* Audiotaped (erotic/sexual) cues | The relation between paedophilia and cognitive function is genuine and not artefactual. The findings were interpreted as evidence for the hypothesis that neurodevelopmental perturbations increase the risk of paedophilia in males |
| Blanchard et al. (2006) | To compare the mean levels of sexual response to children produced by 4 groups of men with sexual offences against prepubescent girls and two comparison groups with other offences/no offences | *Design:* Case-control study  *Country:* Canada  *Setting:* Inpatient clinic  *Follow-up:* NA | *N:* 291  *Gender:* M  *Age category:* Adult  *Diagnosis:* Paedophilia  *Offender type:* Sex offenders: Child and adult victims  *Controls:* M (89): Non-offenders | *Outcome measure:* Change in penis circumference  *Assessment method:* PPG  *Function:* Diagnostic  *Stimulus/task:* (Non-)sexual audio narratives and slides of (non-)sexual stimuli (different ages/genders) | The mean level of paedophilic response in men with offences against daughters or stepdaughters is intermediate between that in men with offences against otherwise-related or unrelated girls and that in men with no offences against girls at all |
| Blanchard et al. (2009) | To decouple the natural association between the absolute and relative magnitude of men’s penile responses to prepubescent girls in the laboratory | *Design:* Cross-sectional study  *Country:* Canada  *Setting:* Inpatient clinic  *Follow-up:* NA | *N:* 402  *Gender:* M  *Age category:* Adult  *Diagnosis:* Paedophilia  *Offender type:* Sex offenders: Child and adult victims  *Controls:* NA | *Outcome measure:* Change in penis circumference  *Assessment method:* PPG  *Function:* Diagnostic  *Stimulus/task:* Audiotaped cues (verbal portrayals of sexual interactions) | The results demonstrate the utility—or perhaps necessity—of relative ascertainment in the laboratory assessment of erotic age–preference |
| Bochkarev et al. (2021) | To examine the changes of evoked EEG oscillations in response to erotic visual stimuli to identify specific patterns in subjects with different variants of paedophilia | *Design:* Case-control study  *Country:* Russia  *Setting:* Correctional facility  *Follow-up:* NA | *N:* 30  *Gender:* M  *Age category:* Adult  *Diagnosis:* Paedophilia  *Offender type:* Sex offenders: Child victims  *Controls:* M (17): Offenders without the condition | *Outcome measure:* Evoked oscillations  *Assessment method:* EEG  *Function:* Etiologic  *Stimulus/task:* Pictures of neutral, heterosexual normative, hetero- and homosexual paedophilic content shortly demonstrated with backward masking | The obtained frequency and spatial patterns of EO changes in response to erotic visual pictures are related to specificity of hetero- and homosexual paedophilia |
| Bontemps et al. (2022) | To examine the self-referential processing of incarcerated youth with psychopathic traits | *Design:* Cross-sectional study  *Country:* USA  *Setting:* Correctional facility  *Follow-up:* NA | *N:* 39  *Gender:* M  *Age category:* Juvenile  *Diagnosis:* Psychopathy  *Offender type:* NS  *Controls:* NA | *Outcome measure:* Brain activity  *Assessment method:* EEG  *Function:* Etiologic  *Stimulus/task:* Processing self-descriptive information (filling in questionnaires) | Findings indicated some withdrawal-motivated activation patterns in the frontal lobes during the processing of self-relevant information |
| Bosch et al. (2022) | To examine the feasibility and acceptance of Sense-IT in forensic psychiatric patients with ASD and/or ID. A secondary aim was to explore trends in aggression and self-harm over a period of time prior to and during the use of biocueing | *Design:* Case-control study  *Country:* The Netherlands  *Setting:* Inpatient clinic  *Follow-up:* 2 weeks | *N:* 15  *Gender:* M (12) + F (3)  *Age category:* Adult  Diagnosis: Other: Autism spectrum disorder and intellectual disability  *Offender type:* NS  *Controls:* M (1) - F (5): Forensic psychiatric nurses | *Outcome measure:* Heart rate  *Assessment method:* Sense-IT (biocueing application; HR)  *Function:* Intervention  *Stimulus/task:* NA (Biocueing) | Participants experienced the biocueing application as positive and are willing to use biocueing |
| Brazil et al. (2012) | To investigate component amplitudes in psychopathic offenders, matched non-psychopathic offenders and healthy controls | *Design:* Case-control study  *Country:* The Netherlands  *Setting:* Inpatient clinic  *Follow-up:* NA | *N:* 43  *Gender:* M  *Age category:* Adult  *Diagnosis:* Psychopathy  *Offender type:* NS  *Controls:* M (16): Healthy controls | *Outcome measure:* ERP, vertical and horizontal eye movement  *Assessment method:* EEG, active electrodes (eye movement)  *Function:* Etiologic  *Stimulus/task:* Three-stimulus oddball paradigm | The results highlight differences between psychopathic and non-psychopathic offenders regarding the integrity of the neurocognitive processes driving attentional allocation, as well as the usefulness of alternative psychophysiological measures in differentiating psychopathy from general antisociality |
| Brazil et al. (2011) | To investigate error-monitoring during the observation of actions in psychopathy | *Design:* Case-control study  *Country:* The Netherlands  *Setting:* Inpatient clinic and outpatient clinic  *Follow-up:* NA | *N:* 18  *Gender:* M  *Age category:* Adult  *Diagnosis:* Psychopathy  *Offender type:* Violent offenders  *Controls:* M (18): Healthy controls | *Outcome measure:* Brain activity, eye movement  *Assessment method:* EEG, EOG  *Function:* Etiologic  *Stimulus/task:* Social flanker task | Although both groups showed similar event-related potentials in response to their actions, amplitudes after the observation of others’ action outcome were greatly reduced in psychopathy. The latter was not unique to observed errors, because the psychopathic group also showed reduced brain potentials after the observation of correct responses |
| Brazil et al. (2009) | To investigate early and late components of error monitoring in violent offenders with psychopathy | *Design:* Case-control study  *Country:* The Netherlands  *Setting:* Inpatient clinic  *Follow-up:* NA | *N:* 16  *Gender:* M  *Age category:* Adult  *Diagnosis:* Psychopathy  *Offender type:* Violent offenders  *Controls:* M (18): Healthy controls | *Outcome measure:* Brain activity, vertical eye *movement*  *Assessment method:* EEG, EOG  *Function:* Etiologic  *Stimulus/task:* Modified version of the Eriksen flanker task | Individuals with psychopathy showed intact early error processing and automatic behavioural adaptation but have deficits in later stages of error processing and controlled behavioural adaptation. This indicates that individuals with psychopathy are unable to effectively use error information to change their behaviour adequately |
| Bresin et al. (2014) | To examine ERN and PES in a sample of individuals with a criminal history | *Design:* Cross-sectional study  *Country:* USA  *Setting:* Correctional facility, inpatient clinic, other  *Follow-up:* NA | *N:* 71  *Gender:* M (49) + F (22)  *Age category:* Adult  *Diagnosis:* Psychopathy/psychopathic traits, antisocial personality disorder  *Offender type:* NS  *Controls:* NA | *Outcome measure:* Brain activity, vertical and horizontal eye movement  *Assessment method:* EEG, EOG  *Function:* Etiologic  *Stimulus/task:* Letter-flanker task | Individuals high in interpersonal-affective traits displayed enhanced adjusting to and monitoring of errors |
| Broom (2011) | To examine the relationship between psychopathy and performance on a modified version of the Iowa Gambling Task in a sample of incarcerated offenders | *Design:* Cross-sectional study  *Country:* USA  *Setting:* Correctional facility  *Follow-up:* NA | *N:* 67  *Gender:* M  *Age category:* Adult  *Diagnosis:* Psychopathy  *Offender type:* NS  *Controls:* NA | *Outcome measure:* Skin conductance, heart rate  *Assessment method:* Coulbourn Instruments 1-cm Ag-AgCl electrodes (SCD, HR assessment)  *Function:* Etiologic  *Stimulus/task:* Iowa gambling task | Psychopathy was found to have an effect on task performance in offenders. Significant correlations were found between PCL-R facet scores and HR and SCR responses to deck selections |
| Buschman et al. (2010) | As the first study on post-conviction polygraphy in the Netherlands, it focuses on cybercrime offenders to address the different child sexual behaviours exhibited by participants who are in treatment for possessing child abuse images | *Design:* Cross-sectional study  *Country:* The Netherlands  *Setting:* Outpatient clinic  *Follow-up:* NA | *N:* 25  *Gender:* M  *Age category:* Adult  *Diagnosis:* Not explicitly stated  *Offender type:* Sex offenders: Child victims  *Controls:* NA | *Outcome measure:* Physiological indicators NOS  *Assessment method:* Polygraph  *Function:* Etiologic  *Stimulus/task:* NA | Post-conviction polygraphy can provide additional data to inform the development of theory and contribute to the treatment, supervision, and more effective containment of offending behaviour and the reduction of future victimization |
| Caldwell et al. (2015) | To investigate whether cocaine abuse held any relationship to the neural systems underlying moral intuition, and what impact psychopathic traits might have on this relationship | *Design:* Case-control study  *Country:* USA  *Setting:* Correctional facility  *Follow-up:* NA | *N:* 87  *Gender:* M  *Age category:* Adult  *Diagnosis:* Psychopathy  *Offender type:* NS  *Controls:* M (87): Offenders, non-cocaine users | *Outcome measure:* Brain activity  *Assessment method:* fMRI  *Function:* Etiologic  *Stimulus/task:* Moral, non-moral, and neutral pictures | Cocaine users higher in psychopathic traits exhibited additional neural abnormalities in the right vSTR and more severe aberration in the right pgACC. These results suggest that regular cocaine abuse may be associated with affective deficits which can impact relatively high-level processes like moral cognition |
| Calzada-Reyes et al. (2012) | To assess the EEG of violent offenders by visual inspection and frequency-domain quantitative analysis | *Design:* Case-control study  *Country:* Cuba  *Setting:* Correctional facility  *Follow-up:* NA | *N:* 50  *Gender:* M  *Age category:* Adult  *Diagnosis:* Antisocial personality disorder  *Offender type:* Violent offenders  *Controls:* M (34): Offenders, no ASPD | *Outcome measure:* Brain activity  *Assessment method:* EEG  *Function:* Etiologic  *Stimulus/task:* NA (resting-state EEG) | QEEG analysis and techniques of source localization may reveal differences in brain electrical activity among offenders with ASPD, which was not obvious to visual inspection |
| Calzada-Reyes et al. (2013) | To compare the QEEG and the current source density measures of violent psychopath offenders to a non-psychopath violent group | *Design:* Case-control study  *Country:* Cuba  *Setting:* Correctional facility  *Follow-up:* NA | *N:* 31  *Gender:* M  *Age category:* Adult  *Diagnosis:* Psychopathy  *Offender type:* Violent offenders  *Controls:* M (27): Offenders, non-psychopathic | *Outcome measure:* Brain activity  *Assessment method:* EEG  *Function:* Etiologic  *Stimulus/task:* NA (resting-state EEG) | Abnormalities in a fronto-temporo-limbic network play a relevant role in the neurobiological basis of psychopathy |
| Calzada-Reyes et al. (2020) | To investigate the presence of electrophysiological differences between male and female psychopath offenders | *Design:* Cross-sectional study  *Country:* Cuba  *Setting:* Correctional facility  *Follow-up:* NA | *N:* 43  *Gender:* M (31) + F (12)  *Age category:* Adult  *Diagnosis:* Psychopathy  *Offender type:* Violent offenders  *Controls:* NA | *Outcome measure:* Brain activity  *Assessment method:* EEG  *Function:* Etiologic  *Stimulus/task:* NA (resting-state EEG) | The resting EEG visual analyses revealed a high percentage of EEG abnormalities in both studied groups |
| Calzada-Reyes et al. (2021) | To replicate and to extend current knowledge related to paralimbic dysfunctions associated with psychopathy | *Design:* Cross-sectional study  *Country:* Cuba  *Setting:* Correctional facility  *Follow-up:* NA | *N:* 132  *Gender:* M (97) + F (35)  *Age category:* Adult  *Diagnosis:* Psychopathy  *Offender type:* Violent offenders  *Controls:* NA | *Outcome measure:* Brain activity  *Assessment method:* EEG  *Function:* Etiologic  *Stimulus/task:* Closed and opened eyes, hyperventilation and recovery | The findings serve to confirm the contribution of functional and structural abnormalities in diverse brain regions, mainly in paralimbic areas, implicated in decision-making, emotion recognition, empathy, cognition and behavioural regulation in psychopathic behaviour |
| Cantor et al. (2016) | To identify analogous differences in functional connectivity in paedophilia | *Design:* Case-control study  *Country:* Canada  *Setting:* Outpatient clinic  *Follow-up:* NA | *N:* 37  *Gender:* M  *Age category:* Adult  *Diagnosis:* Paedophilia  *Offender type:* Sex offenders: Child victims  *Controls:* M (28): Non-sex offenders, M (39): Non-paedophilic men with no criminal history | *Outcome measure:* Functional connectivity of the brain, change in penis circumference  *Assessment method:* fMRI, PPG  *Function:* Etiologic  *Stimulus/task:* Audio narratives and slides of (non-)sexual stimuli (different ages/genders) | This study confirms the presence of significant differences in the functional connectivity of the brain in paedophilia |
| Cantor and Blanchard (2012) | To investigate white matter volumes in paedophiles, hebephiles, and teleiophiles | *Design:* Case-control study  *Country:* Canada  *Setting:* Outpatient clinic  *Follow-up:* NA | *N:* 19  *Gender:* M  *Age category:* Adult  *Diagnosis:* Paedophilia  *Offender type:* Sex offenders: Child victims  *Controls:* M (49): Offenders, hebephilic, M (47): Offenders, teleiophilic | *Outcome measure:* Change in penis circumference  *Assessment method:* PPG  *Function:* Diagnostic  *Stimulus/task:* Audio narratives and slides of (non-)sexual stimuli (different ages/genders) | White matter volumes (neuroanatomy) of hebephiles resembled that of paedophiles more closely than that of teleiophiles |
| Cantor et al. (2004) | To investigate the association between intelligence, memory, handedness, and paedophilia | *Design:* Case-control study  *Country:* Canada  *Setting:* Outpatient clinic  *Follow-up:* NA | *N:* 47  *Gender:* M  *Age category:* Adult  *Diagnosis:* Paedophilia  *Offender type:* Sex offenders: Child victims  *Controls:* M (158): Offenders, hebephilic, M (93): Offenders, teleiophilic | *Outcome measure:* Change in penis circumference  *Assessment method:* PPG  *Function:* Diagnostic  *Stimulus/task:* Audio narratives and slides of (non-)sexual stimuli (different ages/genders) | Paedophilia is negatively associated with IQ, immediate and delayed recall memory, and right-handedness; stated is that these results suggest that paedophilia is linked to early neurodevelopmental perturbations |
| Cantor et al. (2015) | To verify and characterize white matter involvement in paedophilia using diffusion tensor imaging | *Design:* Case-control study  *Country:* Canada  *Setting:* Outpatient clinic  *Follow-up:* NA | *N:* 24  *Gender:* M  *Age category:* Adult  *Diagnosis:* Paedophilia  *Offender type:* Sex offenders: Child victims  *Controls:* M (32): Healthy controls | *Outcome measure:* Change in penis circumference  *Assessment method:* PPG  *Function:* Diagnostic  *Stimulus/task:* Audio narratives and slides of (non-)sexual stimuli (different ages/genders) | Paedophilia is characterized by neuroanatomical differences in white matter microstructure, over and above any neural characteristics attributable to psychopathy and childhood adversity, which show neuroanatomic footprints of their own |
| Cantor et al. (2008) | To identify which brain regions distinguish paedophilic from non-paedophilic men | *Design:* Case-control study  *Country:* Canada  *Setting:* Outpatient clinic  *Follow-up:* NA | *N:* 43  *Gender:* M  *Age category:* Adult  *Diagnosis:* Paedophilia  *Offender type:* Sex offenders: Child victims *Controls:* M (32): Healthy controls | *Outcome measure:* Change in penis circumference  *Assessment method:* PPG  *Function:* Diagnostic  *Stimulus/task:* Audio narratives and slides of (non-)sexual stimuli (different ages/genders) | Paedophilia is negatively associated with white matter volumes of the temporal and parietal lobes, but there is no significant association with (sub)cortical grey matter characteristics |
| Cartocci et al. (2021) | To examine whether criminal offenders would show aberrant connectivity patterns in the brain network devoted to moral behaviour, salience attribution, and reward processing, which would in turn sustain maladaptive behaviour in these individuals | *Design:* Case-control study  *Country:* Italy  *Setting:* Inpatient clinic  *Follow-up:* NA | *N:* 13  *Gender:* M  *Age category:* Adult  *Diagnosis:* Psychotic disorder  *Offender type:* Violent offenders  *Controls:* M (13): Healthy controls | *Outcome measure:* Brain activity  *Assessment method:* fMRI  *Function:* Etiologic  *Stimulus/task:* NA (resting-state fMRI) | Mentally ill persons who were considered socially dangerous and institutionalized in a REMS psychiatric unit displayed higher functional connectivity, compared to a control group, in subcortical networks, especially in brain areas related to morality, reward processing, and impulse control |
| Casey et al. (2013) | To examine experiencing and suppression of emotion processing in psychopathy | *Design:* Cross-sectional study  *Country:* UK  *Setting:* Correctional facility  *Follow-up:* NA | *N:* 61  *Gender:* M  *Age category:* Adult  *Diagnosis:* Psychopathy  *Offender type:* Violent offenders  *Controls:* NA | *Outcome measure:* Heart rate  *Assessment method:* Photoplethysmograph (HR)  *Function:* Etiologic  *Stimulus/task:* Positive and negative images and emotion regulation task | Higher-scoring psychopaths were more cardiovascularly responsive when processing negative information than positive, possibly reflecting an anomalously rewarding aspect of processing normally unpleasant material. When required to experience emotional response higher factor 1 psychopathic individuals showed reduced responsiveness, suggesting that they were less able to do this |
| Castonguay et al. (1993) | To examine whether variables related either to subjects or their crimes would correlate with the magnitude of penile responses during sexual preference assessment | *Design:* Cross-sectional study  *Country:* Canada  *Setting:* Correctional facility and outpatient clinic  *Follow-up:* NA | *N:* 73  *Gender:* M  *Age category:* Adult and juvenile  *Diagnosis:* Paedophilia  *Offender type:* Sex offenders: Child and adult victims  *Controls:* NA | *Outcome measure:* Change in penis circumference  *Assessment method:* PPG  *Function:* Etiologic  *Stimulus/task:* Audiotaped recordings (portrayals of sexual interactions) | This study demonstrated that maximum penile response magnitude is related to both the age and the judiciary status of sex offenders |
| Cazala et al. (2019) | To identify through fMRI the brain responses of male outpatients with paedophilic disorder to visual stimuli depicting children and to compare them with male healthy controls matched on sexual orientation (to female or male adults), age, and handedness | *Design:* Case-control study  *Country:* France  *Setting:* Outpatient clinic  *Follow-up:* NA | *N:* 25  *Gender:* M  *Age category:* Adult  *Diagnosis:* Paedophilia  *Offender type:* Sex offenders: Child victims  *Controls:* M (24): Healthy controls | *Outcome measure:* Brain activity, change in penis circumference  *Assessment method:* fMRI, PPG  *Function:* Etiologic  *Stimulus/task:* Visual stimuli depicting children | Evidence implicates the right and left occipital and temporal gyri, in particular the right inferior temporal gyrus (BA 37), as possible candidate areas mediating SA in patients with paedophilic disorder in response to pictures of undressed children |
| Cazala et al. (2021) | To examine hemodynamic responses to moral pictures, as well as correlations between hemodynamic responses and moral ratings across sadistic and non-sadistic offenders | *Design:* Case-control study  *Country:* USA  *Setting:* Correctional facility  *Follow-up:* NA | *N:* 11  *Gender:* M  *Age category:* Adult  *Diagnosis:* Paraphilia  *Offender type:* Sex offenders: Adult victims  *Controls:* M (10): Offenders, non-sadistic | *Outcome measure:* Brain activity  *Assessment method:* fMRI  *Function:* Etiologic  *Stimulus/task:* Pictures that did or did not depict situations representing moral transgressions | The lack of anterior temporal cortex (ATC) engagement in sadists might be a biomarker of altered moral judgment |
| Cheng et al. (2012) | To investigate the contribution of neural processing associated with the perception of others’ distress in juveniles with disruptive behaviour disorders by using an experimental procedure that assesses the component processes (affective arousal and emotion understanding) of empathy | *Design:* Case-control study  *Country:* China  *Setting:* Correctional facility  *Follow-up:* NA | *N:* 13  *Gender:* M  *Age category:* Juvenile  *Diagnosis:* Juvenile psychopathic traits  *Offender type:* Mixed offender types  *Controls:* M (15): Offenders, low callous-unemotional traits, M (15): Healthy controls | *Outcome measure:* Brain activity  *Assessment method:* EEG  *Function:* Etiologic  *Stimulus/task:* Moral, non-moral, and neutral pictures | Juveniles with high callous-unemotional traits exhibited atypical neural dynamics of pain empathy processing in the early stage of affective arousal, which is coupled with their relative insensitivity to actual pain. Their capacity to understand intentionality, however, was not affected |
| Clark et al. (2022) | To test neural functioning of adolescent offenders with varying degrees of psychopathic traits using EEG spectra analysis | *Design:* Cross-sectional study  *Country:* USA  *Setting:* Correctional facility  *Follow-up:* NA | *N:* 40  *Gender:* M  *Age category:* Juvenile  *Diagnosis:* Psychopathy  *Offender type:* Violent offenders  *Controls:* NA | *Outcome measure:* Brain activity  *Assessment method:* EEG  *Function:* Etiologic  *Stimulus/task:* NA (resting-state EEG) | The findings indicate potentially different brain regions being operative at rest based on component elevations |
| Cohen et al. (2002) | To elucidate psychological and neuropsychiatric factors that could contribute to the motivation for and failure to inhibit paedophilic behaviour | *Design:* Case-control study  *Country:* USA  *Setting:* Outpatient clinic  *Follow-up:* NA | *N:* 22  *Gender:* M  *Age category:* Adult  *Diagnosis:* Paedophilia  *Offender type:* Sex offenders: Child victims  *Controls:* M (24): Healthy controls | *Outcome measure:* Change in penis circumference  *Assessment method:* PPG  *Function:* Etiologic  *Stimulus/task:* Audiotaped narratives of sexual interactions | Subjects with a history of paedophilia as compared with non-paedophilic controls may have persistent abnormalities in brain function, and specifically decreased glucose metabolism, in the temporal and frontal cortices—brain areas implicated in cortical regulation of sexual arousal. Phallometric data show that paedophiles are specifically aroused towards prepubescent children but also may be hyper aroused beyond this deviant choice of object |
| Cohn et al. (2015) | To investigate the distinct associations of juvenile psychopathic traits dimensions with resting state networks connectivity | *Design:* Cross-sectional study  *Country:* The Netherlands  *Setting:* Correctional facility  *Follow-up:* NA | *N:* 130  *Gender:* M (111) + F (9)  *Age category:* Juvenile  *Diagnosis:* Psychopathy  *Offender type:* NS  *Controls:* NA | *Outcome measure:* Brain activity  *Assessment method:* fMRI  *Function:* Etiologic  *Stimulus/task:* NA (resting-state fMRI) | The results confirm the association between psychopathic traits and brain network connectivity, and considerably add to emerging evidence supporting neurobiological heterogeneity in the processes leading to psychopathy |
| Cohn et al. (2015) | To investigate the relationship between the neural correlates of incentive processing and both DBD persistence and psychopathic traits | *Design:* Case-control study  *Country:* The Netherlands  *Setting:* Correctional facility  *Follow-up:* NA | *N:* 22 (Persistent DBD), 23 (Desistent DBD)  *Gender:* M (16) + F (6), M (18) + F (5)  *Age category:* Juvenile  *Diagnosis:* Psychopathy  *Offender type:* Mixed offender types  *Controls:* M (20) - F (13): Healthy controls | *Outcome measure:* Brain activity  *Assessment method:* fMRI  *Function:* Etiologic  *Stimulus/task:* Monetary incentive delay task | Aberrant incentive processing was related to persistence of childhood antisocial behaviour into late adolescence and to callous-unemotional traits. This mechanism may underlie treatment resistance in a subgroup of antisocial youth and provide a target for intervention |
| Cohn et al. (2013) | To investigate the associations between the neural correlates of fear conditioning, persistence of childhood-onset DBD during adolescence and psychopathic traits | *Design:* Case-control study  *Country:* The Netherlands  *Setting:* Correctional facility  *Follow-up:* NA | *N:* 25 (Persistent DBD), 25 (Desistent DBD)  *Gender:* M (18) + F (7), M (20) + F (5)  *Age category:* Juvenile  *Diagnosis:* Psychopathy  *Offender type:* NS  *Controls:* M (23) - F (3): Healthy controls | *Outcome measure:* Brain activity  *Assessment method:* fMRI  *Function:* Etiologic  *Stimulus/task:* Fear conditioning task | Both persistent and desistent DBD subgroups were found to show higher activation in fear processing-related brain areas during fear conditioning compared with healthy controls. Impulsive-irresponsible and grandiose-manipulative psychopathic traits were associated with higher activation, whereas callous-unemotional psychopathic traits were related to lower activation in fear-related areas. The association between neural activation and DBD subgroup membership was mediated by impulsive-irresponsible psychopathic traits |
| Cohn et al. (2016) | To investigate whether brain function during fear extinction is associated with DBD subgroup-membership and psychopathic traits | *Design:* Case-control study  *Country:* The Netherlands  *Setting:* Correctional facility  *Follow-up:* NA | *N:* 25 (Persistent DBD), 25 (Desistent DBD)  *Gender:* M (18) + F (7), M (20) + F (5)  *Age category:* Juvenile  *Diagnosis:* Disruptive behaviour disorder  *Offender type:* Mixed offender types  *Controls:* M (22) - F (3): Healthy controls | *Outcome measure:* Brain activity  *Assessment method:* fMRI  *Function:* Etiologic  *Stimulus/task:* Fear conditioning task | Both DBD persisters and desisters showed hyperreactivity during fear extinction when compared with HCs. Impulsive-irresponsible psychopathic traits were positively associated with responses in the fear neurocircuitry and mediated the association between neural activation and group membership. |
| Contreras-Rodríguez et al. (2014) | To identify both brain activation and task-induced functional connectivity using psychophysiological interaction analysis during an emotional face-matching task in psychopaths and control subjects | *Design:* Case-control study  *Country:* Spain  *Setting:* Correctional facility  *Follow-up:* NA | *N:* 22  *Gender:* M  *Age category:* Adult  *Diagnosis:* Psychopathy  *Offender type:* Violent offenders  *Controls:* M (22): Non-offenders | *Outcome measure:* Brain activity  *Assessment method:* fMRI  *Function:* Etiologic  *Stimulus/task:* Emotional face-matching task | Emotional stimulation may evoke a relevant cortical response in psychopaths, but a disruption in the processing of emotional faces exists involving the reciprocal functional interaction between the amygdala and neocortex, consistent with the notion of a failure to integrate emotion into cognition in psychopathic individuals |
| Contreras-Rodríguez et al. (2015) | To identify abnormal or distinctive functional links between and within emotional and cognitive brain systems in the psychopathic brain to further characterize the neural bases of psychopathy | *Design:* Case-control study  *Country:* Spain  *Setting:* Correctional facility  *Follow-up:* NA | *N:* 22  *Gender:* M  *Age category:* Adult  *Diagnosis:* Psychopathy  *Offender type:* Violent offenders  *Controls:* M (22): Non-offenders | *Outcome measure:* Brain activity  *Assessment method:* fMRI  *Function:* Etiologic  *Stimulus/task:* NA (resting-state fMRI) | A weakened link between emotional and cognitive domains in the psychopathic brain may combine with enhanced functional connections within frontal executive areas |
| Cooper et al. (1990) | To examine a female paedophile who had been both a victim of incest and an active participant and who exhibited multiple paraphilias by extensive clinical, psychometric, endocrine and laboratory sexual arousal studies | *Design:* Case study  *Country:* Canada  *Setting:* NS  *Follow-up:* NA | *N:* 1  *Gender:* F  *Age category:* Adult  *Diagnosis:* Paedophilia  *Offender type:* Sex offender: Child victims  *Controls:* NA | *Outcome measure:* Vaginal blood volume, vaginal pulse rate, vaginal pulse amplitude and response duration  *Assessment method:* Vaginal photoplethysmograph  *Function:* Etiologic  *Stimulus/task:* Sexual slides | The participant's psychiatric, psychometric and physiologic arousal profiles showed similarities to those of a sizable proportion of male child molesters, especially incestors |
| Cope et al. (2014) | To examine the modulatory effect of psychopathic traits on the neurobiological craving response to pictorial drug stimuli | *Design:* Cross-sectional study  *Country:* USA  *Setting:* Correctional facility  *Follow-up:* NA | *N:* 137  *Gender:* M (44) + F (93)  *Age category:* Adult  *Diagnosis:* Psychopathic traits, substance use disorder  *Offender type:* NS  *Controls:* NA | *Outcome measure:* Brain activity  *Assessment method:* fMRI  *Function:* Etiologic  *Stimulus/task:* Drug-related and neutral pictures | Psychopathic traits modulated the neurobiological craving response and results suggest that individual differences are important for understanding and treating substance abuse |
| Corley et al. (1994) | To examine whether there is underlying neuro-organicity contributing to sexually offending behaviour | *Design:* Cross-sectional study  *Country:* USA  *Setting:* Inpatient clinic  *Follow-up:* NA | *N:* 24  *Gender:* NS  *Age category:* NS  *Diagnosis:* Suspected paedophilia  *Offender type:* Sex offenders: Child and adult victims  *Controls:* NA | *Outcome measure:* Brain activity  *Assessment method:* QEEG  *Function:* Etiologic  *Stimulus/task:* NA | In every case, some type of abnormality was noted in the left posterior hemisphere, compared with a normal control population |
| Corominas-Roso et al. (2020) | To assess whether neurofeedback can be useful in the treatment of impulsive behaviour in long-term abstinent cocaine and heroin addicts | *Design:* RCT, longitudinal study  *Country:* Spain  *Setting:* Correctional facility  *Follow-up:* 5 months | *N:* 10  *Gender:* M  *Age category:* Adult  *Diagnosis:* Substance use disorder  *Offender type:* Violent offenders  *Controls:* M (10): Offenders, control group | *Outcome measure:* Brain activity  *Assessment method:* EEG  *Function:* Intervention  *Stimulus/task:* Neurofeedback | NFB is better than placebo in the modulation of impulsivity in this population of long-term abstinent cocaine- and heroin-dependent individuals, and the improvement of impulsivity is crucial to prevent relapse |
| Craft et al. (1962) | To outline psychological, psychiatric, and EEG characteristics that would be of diagnostic use in implementing the provisions of the Mental Health Act, and outlining clinical sub-groups that might have predictive value or respond differentially to contrasting treatment patterns | *Design:* Cross-sectional study  *Country:* UK  *Setting:* Inpatient clinic  *Follow-up:* NA | *N:* 100  *Gender:* M  *Age category:* Adult and juvenile  *Diagnosis:* Psychopathy  *Offender type:* Sex offenders: Child and adult victims  *Controls:* NA | *Outcome measure:* Brain activity  *Assessment method:* EEG  *Function:* Etiologic  *Stimulus/task:* Bemegride (central nervous system stimulant, drug) activation | The LEG study showed a marked contrast between the total incidence of abnormalities after injection of bemegride in the various controls used (17 per cent.), and in the group of psychopathic admissions (73 per cent.) tested |
| Crowley et al. (2010) | To examine whether brain activation would differ during the processing of risky decisions and resulting rewards and punishments between abstinent ASD boys and comparison boys | *Design:* Case-control study  *Country:* USA  *Setting:* Inpatient clinic  *Follow-up:* NA | *N:* 20  *Gender:* M  *Age category:* Juvenile  *Diagnosis:* Substance use disorder  *Offender type:* NS  *Controls:* M (20): Healthy controls | *Outcome measure:* Brain activity  *Assessment method:* fMRI  *Function:* Etiologic  *Stimulus/task:* Colorado balloon game | Adolescent boys with ASD had extensive neural hypoactivity during risky decision-making, coupled with decreased activity during reward and increased activity during loss. These neural patterns may underlie the dangerous, excessive, sustained risk-taking of such boys |
| da Cunha-Bang et al. (2019) | To estimate amygdala reactivity to angry and fearful faces with fMRI in a cohort of men with varying degrees of aggressive traits within both ends of the continuum (violent offenders and non-violent healthy control subjects) | *Design:* Case-control study  *Country:* Denmark  *Setting:* Correctional facility  *Follow-up:* NA | *N:* 16  *Gender:* M  *Age category:* Adult  *Diagnosis:* Psychopathy  *Offender type:* Violent offenders  *Controls:* M (27): Healthy controls | *Outcome measure:* Brain activity  *Assessment method:* fMRI  *Function:* Etiologic  *Stimulus/task:* Emotional faces paradigm | This study shows that an underlying "impulsive aggression" construct, reflected by a commonly used set of personality measures is positively associated with amygdala activation during implicit processing of fearful faces |
| da Cunha-Bang et al. (2017) | To examine neural responses to provocations and aggression using a laboratory model of reactive aggression | *Design:* Case-control study  *Country:* Denmark  *Setting:* Correctional facility  *Follow-up:* NA | *N:* 18  *Gender:* M  *Age category:* Adult  *Diagnosis:* Mixed personality disorder  *Offender type:* Violent offenders  *Controls:* M (26): Healthy controls | *Outcome measure:* Brain activity  *Assessment method:* fMRI  *Function:* Etiologic  *Stimulus/task:* Monetary task | Violent offenders display abnormally high neural reactivity to provocations within the amygdala and striatum and that this sensitivity is related to aggressive behaviour. Violent offenders showed reduced amygdala-prefrontal and striatoprefrontal connectivity in the context of provocations |
| Dargis et al. (2018) | To examine whether the severity of interpersonal and affective psychopathic traits would be associated with fewer visual fixations to the eye region of fear faces, fewer visual fixations to fear faces, lower recognition accuracy of fear faces, and lower negative valence rating of fear faces | *Design:* Cohort study  *Country:* USA  *Setting:* Correctional facility  *Follow-up:* 12 months (for the participants that completed two tasks) | *N:* 108  *Gender:* M  *Age category:* Adult  *Diagnosis:* Psychopathy  *Offender type:* NS  *Controls:* NA | *Outcome measure:* Eye movement  *Assessment method:* Eye tracker (ASL D6 desk-mounted)  *Function:* Etiologic  *Stimulus/task:* Facial emotion recognition task and facial emotion free view task | The interpersonal-affective traits of psychopathy were significantly related to fewer fixations to the eyes of fear faces during the emotion recognition task |
| de Barros (2013) | To investigate the association between PCL-R scores and levels of autonomic reactivity and the degree of callousness as shown by impaired autonomic reactivity during the presentation of unpleasant pictures | *Design:* Cross-sectional study  *Country:* Brazil  *Setting:* Correctional facility  *Follow-up:* NA | *N:* 30  *Gender:* M  *Age category:* Adult  *Diagnosis:* Psychopathy  *Offender type:* Violent offenders  *Controls:* NA | *Outcome measure:* Electrodermal activity  *Assessment method:* SCD (six-channel J&J I-330 oscilloscope)  *Function:* Etiologic  *Stimulus/task:* Emotion-eliciting pictures of empathic content | The results extend previous findings indicating a direct relationship between EDA and psychopathy and suggest that separate investigations of the two PCL-R factors have the potential to unravel more complex relationships between EDA and psychopathy |
| de Looff (2019) | To investigate whether heart rate and electrodermal activity can be used to signal imminent aggression | *Design:* Cohort study  *Country:* The Netherlands  *Setting:* Inpatient clinic  *Follow-up:* 5 days | *N:* 100  *Gender:* M (68) + F (32)  *Age category:* Adult  *Diagnosis:* Psychopathy  *Offender type:* NS  *Controls:* NA | *Outcome measure:* Electrodermal activity, blood volume pulse, skin temperature, movement  *Assessment method:* Empatica E4 wristband  *Function:* Prognostic  *Stimulus/task:* NA | Heart rate, skin conductance level, and the number of non-specific skin conductance responses per minute rose significantly in the 20 min preceding aggressive incidents. Although psychopathy was modestly correlated with displaying aggression, it was not a significant predictor of heart rate and skin conductance preceding aggression |
| de Vries-Bouw (2012) | To investigate salivary alpha-amylase, cortisol, heart rate, and heart rate variability in response to a standardized public speaking task, and examine interactions between these parameters and disruptive behaviour | *Design:* Case-control study  *Country:* The Netherlands  *Setting:* Correctional facility  *Follow-up:* NA | *N:* 48  *Gender:* M  *Age category:* Adult and juvenile  *Diagnosis:* Disruptive behaviour disorder  *Offender type:* NS  *Controls:* M (16): Healthy controls | *Outcome measure:* Heart rate, heart rate variability  *Assessment method:* VU-AMS  *Function:* Etiologic  *Stimulus/task:* Public speaking task | Alpha-amylase and cortisol reactivity, but not HR or HRV, showed significant inverse associations with dimensional measures of disruptive behaviour. There were no interactions between alpha-amylase and cortisol or HRV in relation to disruptive behaviour |
| Decety et al. (2015) | To examine socio-emotional processing of morally-laden behaviour in criminal offenders with high/low levels of psychopathy by measuring the neuro hemodynamic response elicited by viewing and interpreting the emotional consequences of such actions on others | *Design:* Case-control study  *Country:* USA  *Setting:* Correctional facility  *Follow-up:* NA | *N:* 105  *Gender:* M  *Age category:* Adult  *Diagnosis:* Psychopathy  *Offender type:* NS  *Controls:* M (50): Offenders, low psychopathy | *Outcome measure:* Brain activity  *Assessment method:* fMRI  *Function:* Etiologic  *Stimulus/task:* Dynamic visual stimuli depicting ecologically valid dyadic social interactions that resulted in either harm or assistance | Significant hemodynamic differences were detected in the posterior superior temporal sulcus, amygdala, insula, ventral striatum, and prefrontal cortex when individuals with high psychopathy viewed negative versus positive moral scenarios and when they evaluated the emotional responses of the protagonists |
| Decety et al. (2013) | To examine the extent to which perspective-taking can elicit an emotional response in psychopaths | *Design:* Case-control study  *Country:* USA  *Setting:* Correctional facility  *Follow-up:* NA | *N:* 37  *Gender:* M  *Age category:* Adult  *Diagnosis:* Psychopathy  *Offender type:* NS  *Controls:* M (44): Offenders, medium psychopathy, M (40): Offenders, low psychopathy | *Outcome measure:* Brain activity  *Assessment method:* fMRI  *Function:* Etiologic  *Stimulus/task:* Visual stimuli depicting bodily injuries | While individuals with psychopathy exhibited a strong response in pain-affective brain regions when taking an imagine-self perspective, they failed to recruit the neural circuits that are were activated in controls during an imagine-other perspective, which may contribute to lack of empathic concern |
| Decety et al. (2014) | Adult incarcerated males scoring high, medium, and low on psychopathy underwent fMRI scanning while viewing dynamic facial expressions of fear, sadness, happiness, and pain to examine whether deficits are specific to particular emotions | *Design:* Case-control study  *Country:* USA  *Setting:* Correctional facility  *Follow-up:* NA | *N:* 27  *Gender:* M  *Age category:* Adult  *Diagnosis:* Psychopathy  *Offender type:* NS  *Controls:* M (25): Offenders, medium psychopathy, M (28): Offenders, low psychopathy | *Outcome measure:* Brain activity  *Assessment method:* fMRI  *Function:* Etiologic  *Stimulus/task:* Video stimuli of facial expressions of fear, sadness, happiness and pain | Individuals scoring high on psychopathy had consistently less activation than controls in relevant brain regions during the viewing of dynamic video clips of happy, sad, fearful, and pain expressions |
| Decety et al. (2013) | To identify potential differences in patterns of neural activity in incarcerated psychopaths and incarcerated controls during the perception of empathy-eliciting stimuli depicting other people in pain | *Design:* Case-control study  *Country:* USA  *Setting:* Correctional facility  *Follow-up:* NA | *N:* 27  *Gender:* M  *Age category:* Adult  *Diagnosis:* Psychopathy  *Offender type:* NS  *Controls:* M (25): Offenders, medium psychopathy, M (28): Offenders, low psychopathy | *Outcome measure:* Brain activity  *Assessment method:* fMRI  *Function:* Etiologic  *Stimulus/task:* Videoclips (pain interactions task and pain expressions task) | In response to pain cues expressed by others, psychopaths exhibited deficits in vmPFC and OFC regardless of stimulus type, but displayed selective impairment in processing facial cues of distress in regions associated with cognitive mentalizing |
| Deeley (2006) | To investigated brain function in individuals with psychopathy and a control group while processing facial emotion | *Design:* Case-control study  *Country:* UK  *Setting:* Inpatient clinic and outpatient clinic  *Follow-up:* NA | *N:* 6  *Gender:* M  *Age category:* Adult  *Diagnosis:* Psychopathy  *Offender type:* Mixed offender types  *Controls:* M (9): Healthy controls | *Outcome measure:* Brain activity  *Assessment method:* fMRI  *Function:* Etiologic  *Stimulus/task:* Implicit emotion processing task using fearful, happy and neutral faces | People with psychopathy have biological differences from controls when processing facial emotion, and the pattern of response differs according to emotion type |
| Delfin et al. (2019) | To investigate the feasibility of including neuroimaging data in the prediction of recidivism by studying whether the inclusion of resting-state regional cerebral blood flow measurements leads to an incremental increase in predictive performance over traditional risk factors | *Design:* Cohort study  *Country:* Sweden  *Setting:* Inpatient clinic  *Follow-up:* Beginning at intake date and lasting until reconviction, death, deportation or until the end of follow-up (31/12/2013) | *N:* 44  *Gender:* M (39) + F (5)  *Age category:* Adult  *Diagnosis:* Psychopathy  *Offender type:* NS  *Controls:* NA | *Outcome measure:* Brain activity  *Assessment method:* SPECT  *Function:* Prognostic  *Stimulus/task:* NA | Results supported the feasibility of including neuroimaging data in the prediction of recidivism in forensic psychiatric patients |
| Delfin et al. (2020) | To examine whether NoGo N2 and P3 amplitudes would be correlated with self-reported trait disinhibition and with response inhibition accuracy and whether NoGo N2 and P3 latencies would be correlated with self-reported trait disinhibition and response inhibition accuracy | *Design:* Case-control study  *Country:* Sweden  *Setting:* Inpatient clinic  *Follow-up:* NA | *N:* 27  *Gender:* M  *Age category:* Adult  *Diagnosis:* Mixed diagnoses  *Offender type:* Violent offenders  *Controls:* M (20): Healthy controls | *Outcome measure:* Brain activity  *Assessment method:* fMRI  *Function:* Etiologic  *Stimulus/task:* Go-NoGo task | This study demonstrated attenuated NoGo P3 amplitude and delayed NoGo P3 latency in violent, inpatient MDOs compared to healthy controls |
| Delfin et al. (2021) | To examine whether NoGo N2 and P3 amplitudes would be correlated with self-reported trait disinhibition and with response inhibition accuracy and whether NoGo N2 and P3 latencies would be correlated with self-reported trait disinhibition and response inhibition accuracy | *Design:* Case-control study  *Country:* Sweden  *Setting:* Inpatient clinic  *Follow-up:* NA | *N:* 26  *Gender:* M  *Age category:* Adult  *Diagnosis:* Not explicitly stated  *Offender type:* Violent offenders  *Controls:* M (20): Healthy controls | *Outcome measure:* Brain activity  *Assessment method:* fMRI  *Function:* Etiologic  *Stimulus/task:* Go-NoGo task | Scores on the LHA Aggression and Antisocial subscales were robustly associated with longer NoGo P3 latency, and less robustly with longer NoGo N2 latency |
| Deming (2021) | To examine whether psychopathy is related to a diminished role of SN in switching between primarily DMN and primarily FPN activity | *Design:* Case-control-study  *Country:* USA  *Setting:* Correctional facility  *Follow-up:* NA | *N:* 148  *Gender:* M  *Age category:* Adult  *Diagnosis:* Psychopathy  *Offender type:* NS  *Controls:* M (48): Offenders, low psychopathy | *Outcome measure:* Brain activity  *Assessment method:* fMRI  *Function:* Etiologic  *Stimulus/task:* NA | The previously demonstrated switching effect of SN was replicated in a subgroup of low psychopathy participants. SN’s switching role was significantly reduced in high psychopathy participants, and analysis of model parameters revealed that SN modulation of FPN-to-DMN connectivity was uniquely impaired |
| Deming et al. (2023) | To examine whether the salience network's role in switching between states predominated by default mode network activity and states predominated by frontoparietal network activity is impaired in psychopathy | *Design:* Case-control-study  *Country:* USA  *Setting:* Correctional facility  *Follow-up:* NA | *N:* 148  *Gender:* M  *Age category:* Adult  *Diagnosis:* Psychopathy  *Offender type:* NS  *Controls:* M (203): Offenders, low psychopathy | *Outcome measure:* Brain activity  *Assessment method:* fMRI  *Function:* Etiologic  *Stimulus/task:* NA | SN’s switching role was significantly diminished in high psychopathy participants, which corroborates a novel theory of brain function in psychopathy |
| Deming et al. (2020) | To assess the generality of the affective perspective-taking deficit in psychopathy and assess the possibility of anomalies in brain responsiveness to conditions eliciting affective perspective-taking | *Design:* Cross-sectional study  *Country:* USA  *Setting:* Correctional facility  *Follow-up:* NA | *N:* 94  *Gender:* M  *Age category:* Adult  *Diagnosis:* Psychopathy  *Offender type:* NS  *Controls:* NA | *Outcome measure:* Brain activity  *Assessment method:* fMRI  *Function:* Etiologic  *Stimulus/task:* Static images portraying two individuals interacting with one individual’s face obscured by a shape | Psychopathy was negatively related to task accuracy during affective perspective-taking for fear, happiness, and sadness and to reduced hemodynamic activity during fear perspective-taking in several areas which adds to growing evidence of psychopathy-related abnormalities in a cognitive mechanism related to empathy |
| Deming et al. (2022) | To test whether psychopathy is associated with alterations in behavioural and physiological responses to prototypical facial emotion expressions | *Design:* Cross-sectional study  *Country:* USA  *Setting:* Correctional facility  *Follow-up:* NA | *N:* 88  *Gender:* M  *Age category:* Adult  *Diagnosis:* Psychopathy  *Offender type:* NS  *Controls:* NA | *Outcome measure:* Skin conductance, heart rate, facial electromyographic activity  *Assessment method:* The BIOPAC MP160 (SCD, EMG, HR)  *Function:* Etiologic  *Stimulus/task:* Facial expression task | The findings failed to identify aberrant behavioural and physiological responses to prototypical facial emotion expressions in relation to psychopathy |
| Deming et al. (2018) | To examine whether Factor 1 psychopathic traits would be associated with greater activity during self-focused judgments, relative to other-focused judgments, in cortical midline brain regions involved in social cognition | *Design:* Cross-sectional study  *Country:* USA  *Setting:* Correctional facility  *Follow-up:* NA | *N:* 60  *Gender:* M  *Age category:* Adult  *Diagnosis:* Psychopathy  *Offender type:* NS  *Controls:* NA | *Outcome measure:* Brain activity  *Assessment method:* fMRI  *Function:* Etiologic  *Stimulus/task:* Trait judgment task | Factor 1 scores were not significantly related to neural activity during self- or other-judgments. Factor 2 traits were associated with diminished activation to self-judgments, in relation to other-judgments in bilateral posterior cingulate cortex and right temporoparietal junction |
| Dengerink and Bertilson (1975) | To examine the physiological arousal of psychopaths/non-psychopaths while engaged in a social task | *Design:* Case-control study  *Country:* USA  *Setting:* Correctional facility  *Follow-up:* NA | *N:* 22  *Gender:* M  *Age category:* Adult  *Diagnosis:* Psychopathy  *Offender type:* NS  *Controls:* M (30): Offenders, mixed psychopathy group, M (40): Offenders, non-psychopathic | *Outcome measure:* Skin conductance, heart rate  *Assessment method:* Polygraph  *Function:* Etiologic  *Stimulus/task:* Buss aggression task | The current findings indicate that in mildly stressful social situations psychopaths may evidence relative patterns of arousal which are similar to those observed when the subjects are passive recipients of physical stimuli |
| Denomme and Shane (2019) | To evaluate the influence of withdrawal status on the magnitude of biases in neural reactivity to drug-related and food-related stimuli | *Design:* Case-control study  *Country:* USA  *Setting:* Correctional facility  *Follow-up:* NA | *N:* 44  *Gender:* M (NS) + F (NS) (Withdrawal), M (NS) + F (NS) (No withdrawal)  *Age category:* Adult  *Diagnosis:* Psychopathy  *Offender type:* NS  *Controls:* M - F (57): Offenders, no substance use disorder | *Outcome measure:* Brain activity  *Assessment method:* fMRI  *Function:* Etiologic  *Stimulus/task:* Cue-elicited craving task | Biases in neural reactivity towards drug- versus food-related cues only occurred among the WD participants. Withdrawal status may be an important factor to consider when interpreting dependence-related biases in neural reactivity following reward-related cues |
| Denomme et al. (2018) | To examine drug- and food-related hemodynamic signal-change differences in the insula, DMPFC, ACC, amygdala, and striatum between dependent and non-dependent groups, whether psychopathic traits would mediate neural reactivity to drug versus food stimuli in the dependent group, and whether psychopathic traits would interact with substance use | *Design:* Cross-sectional study  *Country:* USA  *Setting:* Correctional facility  *Follow-up:* NA | *N:* 105  *Gender:* M (70) + F (35)  *Age category:* Adult  *Diagnosis:* Psychopathy  *Offender type:* NS  *Controls:* NA | *Outcome measure:* Brain activity  *Assessment method:* fMRI  *Function:* Etiologic  *Stimulus/task:* Cue-elicited craving task | These results help reconcile prior studies on psychopathy and drug-stimulus processing and provide neurocognitive support for the notion that psychopathic traits serve as an underlying risk factor for substance use disorder |
| Dobbs and Speck (1968) | To examine whether there are differences between a group of antisocial individuals and a matched group of controls with respect to VTR characteristics with respect to frequency density spectra | *Design:* Case-control study  *Country:* USA  *Setting:* Inpatient clinic  *Follow-up:* NA | *N:* 14  *Gender:* M  *Age category:* Adult  *Diagnosis:* Mixed diagnoses  *Offender type:* Violent offenders  *Controls:* M (10) - F (4): Staff members | *Outcome measure:* Brain activity  *Assessment method:* EEG  *Function:* Etiologic  *Stimulus/task:* Light flashes | The subjects differed from controls in having increased amplitude of response of OIII to light flashes, greater incidence of temporal theta activity, and a tendency to lack a change in spectral density pattern during stimulation as compared to the resting state |
| Dolan and Fullam (2009) | To examine whether violent patients with schizophrenia who have high psychopathy scores show attenuated amygdala responses to emotional (particularly fearful) faces compared with those with low psychopathy scores | *Design:* Case-control study  *Country:* Australia  *Setting:* Inpatient clinic  *Follow-up:* NA | *N:* 12  *Gender:* M  *Age category:* Adult  *Diagnosis:* Psychopathy  *Offender type:* Violent offenders  *Controls:* M (12): Patients, low psychopathy | *Outcome measure:* Brain activity  *Assessment method:* fMRI  *Function:* Etiologic  *Stimulus/task:* Implicit face affect processing task | Patients with schizophrenia and high levels of psychopathic traits appear to have blunted amygdala responses to fearful faces. At a dimensional level, psychopathy subfacets show a differential relationship to functioning in amygdala-prefrontal circuitry |
| Dreßing et al. (2001) | To examine whether a homosexual paedophile patient differs from healthy heterosexual controls in the activation of neuronal networks when visually stimulated by sexually significant stimuli | *Design:* Case-control study  *Country:* Germany  *Setting:* Inpatient clinic  *Follow-up:* NA | *N:* 1  *Gender:* M  *Age category:* Adult  *Diagnosis:* Paedophilia  *Offender type:* Sex offender: Child victims  *Controls:* M (2): Healthy controls | *Outcome measure:* Brain activity  *Assessment method:* fMRI  *Function:* Etiologic  *Stimulus/task:* Arousing visual stimuli | Presentation of the pictures of boys resulted in the paedophilic offender in a significant activation of the attention network and the right orbitofrontal cortex. In the controls, there was no activation of left hemispheric areas relevant for speech |
| Drislane et al. (2013) | To examine whether psychopaths show generally reduced amplitude of probe P3 response, reflecting diminished cortical post-processing of noise-probe stimuli, relative to non-psychopaths | *Design:* Case-control study  *Country:* USA  *Setting:* Correctional facility  *Follow-up:* NA | *N:* 49  *Gender:* M  *Age category:* Adult  *Diagnosis:* Psychopathy  *Offender type:* NS  *Controls:* M (29): Offenders, non-psychopathic | *Outcome measure:* Amplitude of P3 response  *Assessment method:* EEG  *Function:* Etiologic  *Stimulus/task:* Abrupt noise probes presented during the viewing of pleasant, neutral and unpleasant pictures | The findings demonstrate a reduced cortical orienting response to abrupt aversive stimuli in participants exhibiting features of psychopathy that are distinct from ASPD |
| Duindam et al. (2021) | To examine to what extent baseline-resting heart rate and heart rate variability can be used as markers of CU in incarcerated juvenile and adult offenders | *Design:* Cross-sectional study  *Country:* The Netherlands  *Setting:* Correctional facility  *Follow-up:* NA | *N:* 190  *Gender:* M  *Age category:* Adult and juvenile  *Diagnosis:* Psychopathy  *Offender type:* Mixed offender types  *Controls:* NA | *Outcome measure:* Heart rate, heart rate variability  *Assessment method:* ECG  *Function:* Etiologic  *Stimulus/task:* Aquatic film clip | Impaired cardiac autonomic activity is related to CU traits in juveniles, suggesting that socioemotional processing difficulties should be considered in understanding these deficits |
| Eisenbarth et al. (2013) | To assess whether previously reported behavioral differences would be reflected in differential brain activity in a sample of female offenders | *Design:* Case-control study  *Country:* Italy  *Setting:* Inpatient clinic  *Follow-up:* NA | *N:* 13  *Gender:* F  *Age category:* Adult  *Diagnosis:* Psychopathy  *Offender type:* NS  *Controls:* F (10): Patients, low psychopathy | *Outcome measure:* Brain activity  *Assessment method:* EEG  *Function:* Etiologic  *Stimulus/task:* Emotional facial expressions (i.e., fear, angry, and happy) | Results are consistent with a cortical deficit in processing facial expression of negative emotions in psychopathic men |
| Espinoza et al. (2019) | To examine whether by analyzing time-varying resting state functional network connectivity states obscured in traditional static connectivity analysis can be uncovered and networks both within and outside limbic/paralimbic regions associated with psychopathic traits not revealed by seed or region of interest analysis can be identified | *Design:* Case-control study  *Country:* USA  *Setting:* Correctional facility  *Follow-up:* NA | *N:* 903  *Gender:* M  *Age category:* Adult  *Diagnosis:* Psychopathy  *Offender type:* NS  *Controls:* M (82): Healthy controls | *Outcome measure:* Functional network connectivity  *Assessment method:* fMRI  *Function:* Etiologic  *Stimulus/task:* NA (resting-state fMRI) | This study observed several significant differences in dynamic connectivity measures attributable to individual factor elements of psychopathy, as well unique patterns attributable, categorically, to those meeting full diagnostic criteria for psychopathy |
| Espinoza et al. (2018) | To examine the neural correlates associated with psychopathy to improve early assessment and perhaps inform treatments for this condition | *Design:* Cross-sectional study  *Country:* USA  *Setting:* Correctional facility  *Follow-up:* NA | *N:* 985  *Gender:* M  *Age category:* Adult  *Diagnosis:* Psychopathy  *Offender type:* NS  *Controls:* NA | *Outcome measure:* Brain functional connectivity  *Assessment method:* fMRI  *Function:* Etiologic  *Stimulus/task:* NA (resting-state fMRI) | The affective and interpersonal symptoms of psychopathy are associated with aberrant connectivity in multiple brain networks, including paralimbic regions |
| Fanti et al. (2020) | To examine differences in amygdala activity during threat acquisition and extinction between primary and secondary variants and to investigate whether amygdala activation during threat conditioning is lateralized to the left or the right amygdala | *Design:* Cross-sectional study  *Country:* The Netherlands  *Setting:* Correctional facility  *Follow-up:* NA | *N:* 23 (Anxious), 25 (Primary Psychopathy), 27 (Secondary Psychopathy), 18 (Abuse)  *Gender:* M (NS) + F (NS)  *Age category:* Juvenile  *Diagnosis:* Psychopathy  *Offender type:* NS  *Controls:* NS (43): Offenders, low risk group | *Outcome measure:* Brain activity  *Assessment method:* fMRI  *Function:* Etiologic  *Stimulus/task:* Differential delay threat-conditioning task | Subjects in the primary group showed lower right amygdala activity in response to neutral male faces compared to the low, anxiety and secondary group, whereas subjects in the group with history of abuse exhibited higher right amygdala activity during threat acquisition compared to the rest of the groups. During threat extinction, the primary group showed lower right amygdala activity compared to the secondary and abuse groups |
| Fazio et al. (2017) | To investigate the possible differences in height and/or leg length in paedophilic, hebephilic, and teleiophilic individuals | *Design:* Case-control study  *Country:* USA  *Setting:* Outpatient clinic  *Follow-up:* NA | *N:* 20  *Gender:* M  *Age category:* Adult  *Diagnosis:* Paedophilia  *Offender type:* Sex offenders: Child victims  *Controls:* M (36): Offenders, hebephilic, M (46): Offenders, teleiophilic | *Outcome measure:* Change in penis circumference  *Assessment method:* PPG  *Function:* Diagnostic  *Stimulus/task:* Audio narratives and slides of (non-)sexual stimuli (different ages/genders) | Paedophiles demonstrated reduced measured height and reduced leg length as compared with teleiophiles. This contributes additional evidence to a biological, developmental origin of paedophilia |
| Fede (2017) | To explore the role of network engagement and components during moral processing | *Design:* Case-control study  *Country:* USA  *Setting:* Correctional facility  *Follow-up:* NA | *N:* 539  *Gender:* M (309) + F (230)  *Age category:* Adult  *Diagnosis:* Psychopathy  *Offender type:* NS  *Controls:* M (39,3 %) - F (60,7 %): Healthy controls | *Outcome measure:* Brain activity  *Assessment method:* fMRI  *Function:* Etiologic  *Stimulus/task:* Moral processing task | Patterns of neural engagement and connectivity consistent with proposed models of moral cognition were found. Age, IQ, and sex moderated neural engagement and connectivity during moral cognition in brain regions including the temporoparietal junction and prefrontal cortex. Incarcerated individuals differed from community controls on functional network connectivity and dynamism during moral processing and psychopathic traits were related to network engagement in regions including the temporoparietal junction, cingulate, and temporal poles |
| Fede et al. (2016) | To replicate prior psychopathy research on negative moral judgments and to extend this work by examining psychopathy-related abnormalities in the processing of controversial moral stimuli and positive moral processing | *Design:* Cross-sectional study  *Country:* USA  *Setting:* Correctional facility  *Follow-up:* NA | *N:* 245  *Gender:* M  *Age category:* Adult  *Diagnosis:* Psychopathy  *Offender type:* NS  *Controls:* NA | *Outcome measure:* Brain activity  *Assessment method:* fMRI  *Function:* Etiologic  *Stimulus/task:* Phrases describing moral acts or concepts | Psychopathic traits were related to brain abnormalities in moral-processing regions during the processing of controversial moral stimuli. Results also found differential patterns of wrong and not-wrong moral processing that were associated with psychopathy |
| Fenton et al. (1974) | To examine differences between patients with posterior temporal slow wave foci and patients with normal EEG records concerning several developmental, forensic, psychiatric, behavioral, and psychometric variables | *Design:* Case-control study  *Country:* UK  *Setting:* Inpatient clinic  *Follow-up:* NA | *N:* 32  *Gender:* M (25) + F (7)  *Age category:* Adult  *Diagnosis:* Psychopathy  *Offender type:* Mixed offender types  *Controls:* M (NS) - F (NS): Patients, normal EEG | *Outcome measure:* Brain activity  *Assessment method:* EEG  *Function:* Etiologic  *Stimulus/task:* NA | Ratings of the degree of violent behavior manifested by the patients before admission and since their admission revealed a slight trend for those patients with posterior temporal slow wave anomalies to be more violent than patients with normal EEG records (however, not significant) |
| Fielenbach et al. (2018) | To investigate the effects of a theta/sensorimotor rhythm neurofeedback training protocol on levels of impulsivity, levels of drug craving, and actual drug intake in a population of forensic psychiatric patients with a diagnosis of substance use disorder | *Design:* RCT, longitudinal study  *Country:* The Netherlands  *Setting:* Inpatient clinic  *Follow-up:* 10 weeks | *N:* 21  *Gender:* M  *Age category:* Adult  *Diagnosis:* Substance use disorder  *Offender type:* NS  *Controls:* M (21): Patients, treatment-as-usual (TAU) | *Outcome measure:* Brain activity  *Assessment method:* EEG  *Function:* Intervention  *Stimulus/task:* Neurofeedback | Forensic psychiatric patients are able to increase SMR magnitude over the course of neurofeedback training. However, at the group level, the increase in SMR activity was not related to any of the included impulsivity or drug craving measures |
| Fielenbach et al. (2019) | To investigate whether forensic psychiatric patients diagnosed with substance use disorder were able to learn to control EEG-activity based on a sensorimotor rhythm/theta neurofeedback protocol | *Design:* Cohort study  *Country:* The Netherlands  *Setting:* Inpatient clinic  *Follow-up:* 10 weeks | *N:* 19  *Gender:* M  *Age category:* Adult  *Diagnosis:* Substance use disorder  *Offender type:* Mixed offender types  *Controls:* NA | *Outcome measure:* Brain activity  *Assessment method:* EEG  *Function:* Intervention  *Stimulus/task:* Neurofeedback | All patients improved on self-reported impulsivity measures and levels of craving, but only levels of craving were associated with responding to neurofeedback treatment |
| Fielenbach et al. (2019) | To investigate the interpersonal mechanisms responsible for patients’ ability to respond to neurofeedback | *Design:* Single case study  *Country:* The Netherlands  *Setting:* Inpatient clinic  *Follow-up:* Mean duration: 5.4 weeks | *N:* 4  *Gender:* M  *Age category:* Adult  *Diagnosis:* Substance use disorder  *Offender type:* Violent offenders  *Controls:* NA | *Outcome measure:* Brain activity  *Assessment method:* EEG  *Function:* Intervention  *Stimulus/task:* Neurofeedback | There is insufficient evidence for the beneficial effects of a theta/sensorimotor rhythm (SMR) neurofeedback intervention on measures of impulsivity and craving, and there may be great interindividual differences in patients’ ability to regulate cortical activity |
| Fink et al. (2016) | To investigate stimulus-locked ERP components elicited by distractor stimuli in three tasks (VO-Distinct, VO-Repeated, Go/NoGo) as a predictor of treatment discontinuation in a sample of male and female prison inmates | *Design:* Cohort study  *Country:* USA  *Setting:* Correctional facility  *Follow-up:* 12 weeks | *N:* 123  *Gender:* M (46) + F (77)  *Age category:* Adult  *Diagnosis:* Substance use disorder  *Offender type:* NS  *Controls:* NA | *Outcome measure:* ERP  *Assessment method:* EEG  *Function:* Predictive  *Stimulus/task:* Visual distractor task, visual oddball task, equal probability Go-NoGo task | The discontinuation group showed a significantly higher accuracy rate to novel stimuli in the VO-D task, exhibited a less positive P3a amplitude and a less positive PC5 in the VO-D task, exhibited a more negative N200 amplitude in the VOR-R task and displayed less positive PC4 amplitude |
| Firestone et al. (2000) | To compare the prevalence of psychopathy and phallometric indexes of deviant sexual arousal and to study the relationship between phallometrically assessed deviant sexual arousal and psychopathy among incest offenders, child molesters and rapists | *Design:* Cross-sectional study  *Country:* Canada  *Setting:* Outpatient clinic  *Follow-up:* NA | *N:* 156 (Incest offenders), 260 (Child molesters), 123 (Rapists)  *Gender:* M  *Age category:* Adult  *Diagnosis:* Psychopathy  *Offender type:* Sex offenders: Child and adult victims  *Controls:* NA | *Outcome measure:* Change in penis circumference *Assessment method:* Indium-Gallium strain gauge and Farrell Instruments CAT200  *Function:* Etiologic  *Stimulus/task:* Audio narratives and slides of (non-)sexual stimuli (different ages/genders) | Deviant sexual arousal to auditory stimuli was evident only on the Paedophile Index for child molesters. Child molesters evidenced a significant correlation between psychopathy and the Rape Index and psychopathy and the Paedophile Index. There were no such significant findings in the incest offender or rapist groups |
| Florez et al. (2017) | To compare HRV and IAT scores in a sample of inmates to validate and contrast their capacities to detect psychopathy | *Design:* Cross-sectional study  *Country:* Spain  *Setting:* Correctional facility  *Follow-up:* NA | *N:* 204  *Gender:* M (175) + F (29)  *Age category:* Adult  *Diagnosis:* Psychopathy  *Offender type:* NS  *Controls:* NA | *Outcome measure:* Heart rate variability *Assessment method:* Polar WearLink  *Function:* Diagnostic  *Stimulus/task:* Images and words | An HRV experiment is better than IAT in order to detect psychopathy |
| Flor-Henry et al. (1991) | Paedophiles were investigated neurophysiologically in order to verify the possible presence of left hemisphere EEG abnormalities | *Design:* Case-control study  *Country:* Canada  *Setting:* Outpatient clinic  *Follow-up:* NA | *N:* 96  *Gender:* M  *Age category:* Adult  *Diagnosis:* Paedophilia  *Offender type:* Sex offenders: Child victims  *Controls:* M (46): Healthy controls | *Outcome measure:* Brain activity  *Assessment method:* EEG  *Function:* Etiologic  *Stimulus/task:* At rest with eyes open, at rest with a vocabulary exercise and a oral word fluency and a visuospatial exercise | Results suggest in this sexual deviation a neurophysiological instability of the dominant hemisphere with dysregulation of interhemispheric relationship |
| Flor-Henry et al. (1988) | To compare EEG characteristics of male genital exhibitionists with those of a control group | *Design:* Case-control study  *Country:* Canada  *Setting:* Outpatient clinic  *Follow-up:* NA | *N:* 43  *Gender:* M  *Age category:* Adult  *Diagnosis:* Exhibitionistic disorder  *Offender type:* Sex offenders: Victim not specified  *Controls:* M (46): Healthy controls | *Outcome measure:* Brain activity  *Assessment method:* EEG  *Function:* Etiologic  *Stimulus/task:* Eyes open and eyes closed tasks, vocabulary and word fluency tasks, spatial task (block design) | EEG power and coherence were significantly different in the exhibitionist group with regard to left hemispheric functions and inter-hemispheric relationships |
| Forth and Hare (1989) | To examine whether the contingent negative variation is absent or greatly attenuated in psychopaths | *Design:* Case-control study  *Country:* Canada  *Setting:* Correctional facility  *Follow-up:* NA | *N:* 12  *Gender:* M  *Age category:* Adult  *Diagnosis:* Psychopathy  *Offender type:* NS  *Controls:* M (11): Offenders, non-psychopathic | *Outcome measure:* Brain activity, eye movement  *Assessment method:* EEG, EOG  *Function:* Etiologic  *Stimulus/task:* Forewarned reaction time task | The results are consistent with the hypothesis that psychopaths are proficient at focusing attention on events that interest them |
| Freeman et al. (2014) | To examine the degree to which specific DMN subregions are affected in criminal psychopaths, and how activity in these subregions relates to affective/ interpersonal and antisocial/lifestyle traits of psychopathy | *Design:* Case-control study  *Country:* USA  *Setting:* Correctional facility  *Follow-up:* NA | *N:* 22  *Gender:* M  *Age category:* Adult  *Diagnosis:* Psychopathy  *Offender type:* NS  *Controls:* M (22): Offenders, low psychopathy | *Outcome measure:* Brain activity  *Assessment method:* fMRI  *Function:* Etiologic  *Stimulus/task:* Go-NoGo task | Attenuated deactivation of the mPC subregion of the DMN is intrinsic to psychopathy, and is a pattern that may be more associated with affective psychopathic traits |
| Freund (1965) | To investigate the efficacy of a modification of the penis plethysmograph test of sexual interest in the differential diagnosis of heterosexual paedophilia in male adults | *Design:* Case-control study  *Country:* Czech Republic  *Setting:* Correctional facility  *Follow-up:* NA | *N:* 20  *Gender:* M  *Age category:* Adult  *Diagnosis:* Suspected paedophilia  *Offender type:* Sex offenders: Child victims  *Controls:* M (20): Healthy controls | *Outcome measure:* Change in penis circumference  *Assessment method:* PPG  *Function:* Diagnostic  *Stimulus/task:* Pictures of naked men, women and children (of either sex) | The results favor the conclusion that the method described may serve in the detection of heterosexual paedophilia in male adults, but it still remains a crude instrument |
| Freund and Blanchard (1989) | To investigate the sensitivity and specificity of our phallometric test for paedophilia (and hebephilia) | *Design:* Case-control study  *Country:* Canada  *Setting:* Outpatient clinic  *Follow-up:* NA | *N:* 154  *Gender:* M  *Age category:* Adult  *Diagnosis:* Suspected paedophilia  *Offender type:* Sex offenders: Child victims  *Controls:* M (26): Offenders against adult women | *Outcome measure:* Change in penis circumference  *Assessment method:* PPG  *Function:* Diagnostic  *Stimulus/task:* Film strips of nude children and adults walking slowly toward the camera and audiotaped narratives | The high specificity of the test implies that a positive phallometric diagnosis of paedophilia may be the decisive factor in clinical assessment when the patient's age preference cannot be determined with confidence from his history. The sensitivity of this test could not be completely determined |
| Freund and Watson (1992) | To investigate whether the etiology of preferred partner sex among paedophiles is related to the etiology of preferred partner sex among males preferring adult partners | *Design:* Cross-sectional study  *Country:* Canada  *Setting:* Correctional facility  *Follow-up:* NA | *N:* 465  *Gender:* M  *Age category:* Adult  *Diagnosis:* Paedophilia  *Offender type:* Sex offenders: Child victims  *Controls:* NA | *Outcome measure:* Change in penis circumference  *Assessment method:* PPG  *Function:* Diagnostic  *Stimulus/task:* NA | Results suggest that the resulting proportion of true paedophiles among persons with a homosexual erotic development is greater than that in persons who develop heterosexually |
| Freund and Watson (1991) | To assess the specificity and sensitivity of the phallometric test of an erotic preference for minors | *Design:* Case-control study  *Country:* Canada  *Setting:* Correctional facility  *Follow-up:* NA | *N:* 42  *Gender:* M  *Age category:* Adult  *Diagnosis:* Paraphilia  *Offender type:* Sex offenders: Adult victims  *Controls:* M (50): Healthy controls | *Outcome measure:* Change in penis circumference  *Assessment method:* PPG  *Function:* Diagnostic  *Stimulus/task:* Sexual and neutral film clips and slides showing nude 8- to 11-year-old girls and boys and landscapes | The specificity was determined to be 96.9% (sex offenders against female adults), 80.6% (paid volunteers), 78.2% and 88.6% (heterosexual paedophiles and homosexual paedophiles or hebephiles). The sensitivity was determined to be 44.5% (offenders against only 1 female child), 74.6% (offenders against at least 1 female child), and 86.7% (offenders against only 1 male minor (child or pubescent)) |
| Fromberger et al. (2013) | To test whether sexually relevant features of a stimulus are preattentively selected and automatically induce focal attention to these sexually relevant aspects in people suffering from paedophilic interests | *Design:* Case-control study  *Country:* Germany  *Setting:* Inpatient clinic  *Follow-up:* NA | *N:* 22  *Gender:* M  *Age category:* Adult  *Diagnosis:* Paedophilia  *Offender type:* Sex offenders: Child victims  *Controls:* M (8): Forensic control group, M (52): Healthy controls | *Outcome measure:* Eye movement  *Assessment method:* Eye tracker (SMI RED 250)  *Function:* Etiologic  *Stimulus/task:* (Erotic) pictures | The results confirmed the hypothesis that paedophiles automatically selected sexually relevant stimuli (children) |
| Fromberger et al. (2012) | To evaluate the diagnostic accuracy of eye-movement parameters regarding paedophilic sexual preferences | *Design:* Case-control study  *Country:* Germany  *Setting:* Inpatient clinic  *Follow-up:* NA | *N:* 22  *Gender:* M  *Age category:* Adult  *Diagnosis:* Paedophilia  *Offender type:* Sex offenders: Child victims  *Controls:* M (8): Forensic control group, M (52): Healthy controls | *Outcome measure:* Eye movement  *Assessment method:* Eye tracker (SMI RED 250)  *Function:* Diagnostic  *Stimulus/task:* (Erotic) pictures | Despite some methodological limitations, measuring eye movements seems to be a promising approach to assess deviant paedophilic interests. Eye movements, which represent automatic attentional processes, demonstrated high diagnostic accuracy |
| Frommann et al. (2013) | To investigate whether male inpatients with schizophrenia and a history of hands-on violent offenses are more impaired in emotion recognition than matched schizophrenia patients without any history of violence | *Design:* Case-control study  *Country:* Germany  *Setting:* Inpatient clinic  *Follow-up:* NA | *N:* 19  *Gender:* M  *Age category:* Adult  *Diagnosis:* Psychotic disorder  *Offender type:* Violent offenders  *Controls:* M (19): Patients, non-violent | *Outcome measure:* ERP  *Assessment method:* EEG  *Function:* Etiologic  *Stimulus/task:* Pictures of facial affect | The results support the hypothesis that in forensic schizophrenic emotional faces are more salient and evoke higher arousal. Larger impairment in affect recognition performance combined with higher salience and arousal may contribute to the occurrence of violent acts in schizophrenia patients |
| Gehrer et al. (2020) | To examine patterns of visual attention during live social interactions and their association with symptom clusters of psychopathy | *Design:* Cross-sectional study  *Country:* Germany  *Setting:* Correctional facility  *Follow-up:* NA | *N:* 30  *Gender:* M  *Age category:* Adult  *Diagnosis:* Psychopathy  *Offender type:* Mixed offender types  *Controls:* NA | *Outcome measure:* Eye movement  *Assessment method:* Eye tracker (SMI RED 250)  *Function:* Etiologic  *Stimulus/task:* Live social interactions | The data reveal that across both listening and talking conditions, higher affective psychopathy is a significant predictor of reduced eye contact |
| Gehrer et al. (2019) | To investigate whether incarcerated criminals with high psychopathic traits exhibit reduced attention orienting to the eyes when compared to a group of low-psychopathic incarcerated offenders | *Design:* Case-control study  *Country:* Germany  *Setting:* Correctional facility  *Follow-up:* NA | *N:* 19  *Gender:* M  *Age category:* Adult  *Diagnosis:* Psychopathy  *Offender type:* Mixed offender types  *Controls:* M (17): Offenders, non-psychopathic | *Outcome measure:* Eye movement  *Assessment method:* Eye tracker (SMI RED 250)  *Function:* Etiologic  *Stimulus/task:* Gender and emotion categorization task | The results suggest a pervasive impairment to attention orienting toward the eyes in psychopaths compared to non-psychopathic offenders |
| Geurts et al. (2016) | To assess reward expectation and its mechanisms in a criminal sample (incarcerated, psychopathic criminals) | *Design:* Case-control study  *Country:* The Netherlands  *Setting:* Inpatient clinic  *Follow-up:* NA | *N:* 14  *Gender:* M  *Age category:* Adult  *Diagnosis:* Psychopathy  *Offender type:* NS  *Controls:* M (20): Healthy controls | *Outcome measure:* Brain activity  *Assessment method:* fMRI  *Function:* Etiologic  *Stimulus/task:* Monetary incentive delay task | Incarcerated psychopathic criminals can be dissociated from non-criminal individuals with comparable impulsive/antisocial personality tendencies based on the degree to which reward-related brain regions interact with brain regions that control behaviour |
| Gibbels et al. (2019) | To analyze clinical, neuropsychological, and neurobiological features of convicted and non-convicted CSOs to unravel possible factors that might increase or decrease the probability to be convicted | *Design:* Case-control study  *Country:* Germany  *Setting:* Correctional facility and outpatient clinic  *Follow-up:* NA | *N:* 43  *Gender:* M  *Age category:* Adult  *Diagnosis:* Paedophilia  *Offender type:* Sex offenders: Child victims  *Controls:* M (31): Non-convicted CSOs | *Outcome measure:* Brain activity  *Assessment method:* fMRI  *Function:* Etiologic  *Stimulus/task:* Go-NoGo task | Convicted and non-convicted paedophilic CSOs revealed similar clinical characteristics, inhibition performances, and neuronal activation |
| Gibbens et al. (1955) | The criminal records/EEG recordings of prisoners with severe psychopathic personalities were examined to investigate prognosis during a follow-up investigation | *Design:* Cohort study  *Country:* UK  *Setting:* NS  *Follow-up:* 5 years | *N:* 69  *Gender:* M  *Age category:* Adult  *Diagnosis:* Psychopathy  *Offender type:* NS  *Controls:* M (56): Offenders, non-psychopathic | *Outcome measure:* Brain activity  *Assessment method:* EEG  *Function:* Prognostic  *Stimulus/task:* NA | EEG abnormality was found four times as frequently in psychopaths as in controls. EEG abnormality was not related to the after-conduct of psychopaths as a whole. There was evidence that in all psychopaths over 25/inadequate psychopaths abnormality of EEG is a favorable prognostic sign |
| Gillespie et al. (2019) | To examine the relationship of distinct psychopathic traits with recognition accuracy and pupillary responses to varied emotional expressions in a convicted offender sample | *Design:* Case-control study  *Country:* UK  *Setting:* Correctional facility  *Follow-up:* NA | *N:* 73  *Gender:* M  *Age category:* Adult  *Diagnosis:* Psychopathy  *Offender type:* Violent offenders  *Controls:* M (25): Healthy controls | *Outcome measure:* Pupil dilation responses  *Assessment method:* Eye tracker  *Function:* Etiologic  *Stimulus/task:* Facial emotion recognition task | Results highlight a potential role of the locus coeruleus-noradrenaline system in the pathophysiology of psychopathy, and demonstrate the potential of the pupillary response as a technique for understanding attention-emotion interactions in psychopathy |
| Gou et al. (2021) | To build an individualization-oriented prediction model for violence in patients with schizophrenia on the basis of multimodal data and to identify hub regions that contribute to violence to further the understanding of the pathology of violence in schizophrenia | *Design:* Case-control study  *Country:* China  *Setting:* Inpatient clinic  *Follow-up:* NA | *N:* 42  *Gender:* M  *Age category:* Adult  *Diagnosis:* Psychotic disorder  *Offender type:* Violent offenders  *Controls:* M (32): Patients, non-violent | *Outcome measure:* Brain activity  *Assessment method:* fMRI  *Function:* Diagnostic  *Stimulus/task:* NA | This study provides the first evidence supporting that the combination of specific multimodal neuroimaging and clinical data in ML analysis can effectively identify violent patients with schizophrenia |
| Greenberg et al. (1996) | To investigate the relationship between paedophiles with aggressive tendencies and the degree of sexual violence in their paedophilic acts | *Design:* Case-control study  *Country:* Canada  *Setting:* NS  *Follow-up:* NA | *N:* 88  *Gender:* M  *Age category:* Adult  *Diagnosis:* Paedophilia  *Offender type:* Sex offenders: Child victims  *Controls:* M (88): Medium hostility, M (87): Low hostility | *Outcome measure:* Change in penis circumference  *Assessment method:* PPG  *Function:* Etiologic  *Stimulus/task:* Videotape and audiotape narrative of sexual stimuli | Neither of the possible measurements of sexual violence bore any relationship to aggressive and hostile tendencies (non-sexual) in our sample |
| Gregory et al. (2015) | To measure brain activation associated with the representation of punishment or reward information during an event-related probabilistic response-reversal task | *Design:* Case-control study  *Country:* UK  *Setting:* Correctional facility  *Follow-up:* NA | *N:* 22  *Gender:* M  *Age category:* Adult  *Diagnosis:* Mixed diagnoses  *Offender type:* Violent offenders  *Controls:* M (18): Healthy controls | *Outcome measure:* Brain activity  *Assessment method:* fMRI  *Function:* Etiologic  *Stimulus/task:* Probabilistic response-reversal task | Offenders with antisocial personality disorder and psychopathy displayed discrete regions of increased activation in the posterior cingulate cortex and anterior insula in response to punished errors during the task reversal phase, and decreased activation to all correct rewarded responses in the superior temporal cortex |
| Guan et al. (2015) | To explore the course of response inhibition function in juvenile delinquents with antisocial personality characteristics by recording event-related potentials in a Go-Nogo task | *Design:* Case-control study  *Country:* China  *Setting:* Correctional facility  *Follow-up:* NA | *N:* 20  *Gender:* M  *Age category:* Juvenile  *Diagnosis:* Antisocial personality traits  *Offender type:* NS  *Controls:* M (20): Healthy controls | *Outcome measure:* Brain activity  *Assessment method:* EEG  *Function:* Etiologic  *Stimulus/task:* Go-NoGo task | Juvenile delinquents with antisocial personality characteristics exhibited impaired response inhibition function |
| Habermeyer et al. (2013) | To identify neuronal networks related to the immediate processing of erotic stimuli in heterosexual male paedophiles and healthy age-matched controls | *Design:* Case-control study  *Country:* Switzerland  *Setting:* Outpatient clinic  *Follow-up:* NA | *N:* 8  *Gender:* M  *Age category:* Adult  *Diagnosis:* Paedophilia  *Offender type:* Sex offenders: Child victims  *Controls:* M (8): Healthy controls | *Outcome measure:* Brain activity  *Assessment method:* fMRI  *Function:* Etiologic  *Stimulus/task:* Visual erotic stimuli of prepubescent children and adults | Erotic pictures activate some of the brain regions already known to be involved in the processing of erotic pictures, erotic pictures of prepubescent children activate brain regions critical for choosing response strategies in both groups, and erotically salient stimuli selectively activate a brain region in paedophilic subjects that had previously been attributed to reward and punishment |
| Habermeyer et al. (2013) | To assess how inhibition in paedophilia is related to differential recruitment of frontal brain areas | *Design:* Case-control study  *Country:* Switzerland  *Setting:* Outpatient clinic  *Follow-up:* NA | *N:* 11  *Gender:* M  *Age category:* Adult  *Diagnosis:* Paedophilia  *Offender type:* Sex offenders: Child victims  *Controls:* NA | *Outcome measure:* Brain activity  *Assessment method:* fMRI  *Function:* Etiologic  *Stimulus/task:* Go-NoGo task | Slower reaction time and less accurate visual target discrimination in paedophilia was accompanied by attenuated deactivation of brain areas belonging to the default mode network |
| Habermeyer et al. (2012) | To compare visual erotic stimulation pre- and on-treatment with the LH-RH agonist leuprolide acetate in a case of homosexual paedophilia | *Design:* Case study, longitudinal study  *Country:* Switzerland  *Setting:* Correctional facility and outpatient clinic  *Follow-up:* 10 months | *N:* 1  *Gender:* M  *Age category:* Adult  *Diagnosis:* Paedophilia  *Offender type:* Sex offender: Child victims  *Controls:* NA | *Outcome measure:* Brain activity  *Assessment method:* fMRI  *Function:* Monitoring  *Stimulus/task:* Visual erotic and non-erotic stimuli of men and boys | The pre-treatment contrasts of the erotic pictures against the respective neutral pictures showed an activation of the right amygdala and adjacent parahippocampal gyrus that decreased significantly under treatment with leuprolide acetate |
| Hamilton et al. (2014) | Previous work has found that psychopathic individuals show reduced interference on the Box Stroop task, this study sought to replicate and extend these findings | *Design:* Cross-sectional study  *Country:* USA  *Setting:* Correctional facility  *Follow-up:* NA | *N:* 117  *Gender:* M  *Age category:* Adult  *Diagnosis:* Psychopathy  *Offender type:* NS  *Controls:* NA | *Outcome measure:* Frontal N100  *Assessment method:* EEG  *Function:* Etiologic  *Stimulus/task:* Box Stroop task | Psychopathic individuals have less coordinated responses to conflict than healthy individuals, a conjecture that has implications for information integration and self-regulation |
| Hansen et al. (2007) | To investigate whether the four facets of Hare’s Psychopathy Checklist-Revised are related to physiological and cognitive mechanisms | *Design:* Cross-sectional study  *Country:* Norway  *Setting:* Correctional facility  *Follow-up:* NA | *N:* 53  *Gender:* M  *Age category:* Adult  *Diagnosis:* Psychopathy  *Offender type:* Violent offenders  *Controls:* NA | *Outcome measure:* Heart rate, heart rate variability  *Assessment method:* Ambulatory Monitoring System (ECG)  *Function:* Etiologic  *Stimulus/task:* Baseline, continuous performance test, working memory test, recovery | The different facets of psychopathy are differentially associated with both physiological and cognitive functions |
| Hare (1965) | To examine whether the amount of fear elicited by cues associated with punishment decreases as the temporal remoteness of anticipated punishment increases, with the rate of decrease being greater for psychopathic than for non-psychopathic persons | *Design:* Cross-sectional study  *Country:* Canada  *Setting:* Correctional facility  *Follow-up:* NA | *N:* 11  *Gender:* M  *Age category:* Adult  *Diagnosis:* Psychopathy  *Offender type:* NS  *Controls:* M (11): Offenders, non-psychopathic, M (11): Healthy controls | *Outcome measure:* Skin resistance  *Assessment method:* Lafayette GSR recorder  *Function:* Etiologic  *Stimulus/task:* Number presented via a memory drum | The results were consistent with the hypothesis that the temporal gradient of fear arousal and response inhibition is steeper for psychopaths than for normal persons |
| Hare (1968) | To examine possible differences in autonomic activity between primary psychopaths, secondary psychopaths, and non-psychopaths | *Design:* Case-control study  *Country:* Canada  *Setting:* Correctional facility  *Follow-up:* NA | *N:* 39  *Gender:* M  *Age category:* Adult  *Diagnosis:* Psychopathy  *Offender type:* NS  *Controls:* M (12): Offenders, non-psychopathic | *Outcome measure:* Skin resistance, heart rate, digital vasoconstriction, respiration rate  *Assessment method:* Offner Type R Dynograph  *Function:* Etiologic  *Stimulus/task:* Repetitive tones and arithmetic problems | The results lend some support to the hypothesis that the autonomic functioning of the primary psychopath differs from that of non-psychopathic individuals |
| Hare (1978) | To examine possible differences in electrodermal activity between psychopaths, and non-psychopaths | *Design:* Case-control study  *Country:* Canada  *Setting:* Correctional facility  *Follow-up:* NA | *N:* 24  *Gender:* M  *Age category:* Adult  *Diagnosis:* Psychopathy  *Offender type:* NS  *Controls:* M (40): Offenders, non-psychopathic | *Outcome measure:* Skin conductance, heart rate, respiration, vasomotor activity  *Assessment method:* Beckman Type R Dynograph  *Function:* Etiologic  *Stimulus/task:* Fast and slow rise-time tones | The results support the hypothesis that psychopathic inmates are electrodermally hyporesponsive to very strong stimuli but not to weaker stimuli. The group considered to be most psychopathic gave smaller skin conductance responses than did the other group only when the stimulus was most intense |
| Hare (1982) | To examine whether or not psychopaths would exhibit a pattern of heart rate acceleration and little electrodermal arousal when they can physically remove themselves from the premonitory cues | *Design:* Case-control study  *Country:* Canada  *Setting:* Correctional facility  *Follow-up:* NA | *N:* 34  *Gender:* M  *Age category:* Adult  *Diagnosis:* Psychopathy  *Offender type:* NS  *Controls:* M (17): Offenders, low psychopathy | *Outcome measure:* Skin conductance, heart rate  *Assessment method:* Beckman Type R Dynograph  *Function:* Etiologic  *Stimulus/task:* Continuous white noise, recording of a nightclub comedian, continuous tone that changed in frequency | The findings suggest that making use of distraction when an aversive stimulus is avoidable results in less conflict and heightened emotional arousal in psychopaths than in other inmates |
| Hare (1974) | To investigate some of the behavioral and physiological responses of psychopaths in a situation involving interaction with another individual | *Design:* Case-control study  *Country:* Canada  *Setting:* Correctional facility  *Follow-up:* NA | *N:* 17  *Gender:* M  *Age category:* Adult  *Diagnosis:* Psychopathy  *Offender type:* NS  *Controls:* M (17): Offenders, non-psychopathic | *Outcome measure:* Skin conductance, heart rate  *Assessment method:* Polygraph  *Function:* Etiologic  *Stimulus/task:* Mixed-motive game | The electrodermal data were inconsistent with the view that psychopaths experience little fear arousal prior to reception of aversive stimuli by themselves or by others |
| Harenski et al. (2018) | To examine functional connectivity related to mentalizing in psychotic disorders and associations with symptoms | *Design:* Case-control study  *Country:* USA  *Setting:* Correctional facility  *Follow-up:* NA | *N:* 46  *Gender:* M  *Age category:* Adult  *Diagnosis:* Psychotic disorder  *Offender type:* NS  *Controls:* M (41): Offenders, non-psychopathic | *Outcome measure:* Brain activity  *Assessment method:* fMRI  *Function:* Etiologic  *Stimulus/task:* Affective mentalizing task | Psychotic offenders showed impaired task performance and reduced activation in a component comprising the dorsomedial prefrontal cortex, superior temporal gyrus, and ventrolateral prefrontal cortex. Positive and cognitive symptoms were inversely correlated with component activity and task performance |
| Harenski et al. (2014) | Prior studies have found that male psychopathy is associated with reduced limbic and paralimbic activation when processing emotional stimuli and making moral judgments. This study investigated whether these findings extend to female psychopathy | *Design:* Case-control study  *Country:* USA  *Setting:* Correctional facility  *Follow-up:* NA | *N:* 157  *Gender:* F  *Age category:* Adult  *Diagnosis:* Psychopathy  *Offender type:* NS  *Controls:* F (46): Healthy controls | *Outcome measure:* Brain activity  *Assessment method:* fMRI  *Function:* Etiologic  *Stimulus/task:* Moral, non-moral, and neutral pictures | Female psychopathy is characterized by reduced limbic activation during emotion processing. In contrast, reduced temporoparietal activation to moral transgressions has been less observed in male psychopathy |
| Harenski et al. (2014) | Neuroimaging studies have found that adult male psychopaths show reduced engagement of limbic and paralimbic circuitry while making moral judgments. This study investigated whether these findings extend to adolescent males with psychopathic traits | *Design:* Cross-sectional study  *Country:* USA  *Setting:* Correctional facility  *Follow-up:* NA | *N:* 111  *Gender:* M  *Age category:* Juvenile  *Diagnosis:* Psychopathy  *Offender type:* Violent offenders  *Controls:* NA | *Outcome measure:* Brain activity  *Assessment method:* fMRI  *Function:* Etiologic  *Stimulus/task:* Moral, non-moral, and neutral pictures | This study observed negative associations between psychopathic traits and hemodynamic responses in the ATC and amygdala during moral judgments of emotional pictures, which varied across the type of psychopathic traits examined |
| Harenski et al. (2010) | To use fMRI to record hemodynamic activity from incarcerated psychopaths and non-psychopathic offenders during moral decision-making | *Design:* Case-control study  *Country:* USA  *Setting:* Correctional facility  *Follow-up:* NA | *N:* 16  *Gender:* M  *Age category:* Adult  *Diagnosis:* Psychopathy  *Offender type:* NS  *Controls:* M (16): Offenders, non-psychopathic | *Outcome measure:* Brain activity  *Assessment method:* fMRI  *Function:* Etiologic  *Stimulus/task:* Moral, non-moral, and neutral pictures | The results reveal potential neural underpinnings of moral insensitivity in psychopathy |
| Harenski et al. (2012) | To study the neural correlates of pain observation in sexual sadists and non-sadists | *Design:* Case-control study  *Country:* USA  *Setting:* Inpatient clinic  *Follow-up:* NA | *N:* 8  *Gender:* M  *Age category:* Adult  *Diagnosis:* Paraphilia  *Offender type:* Sex offenders: Adult victims  *Controls:* M (7): Patients, non-sadistic | *Outcome measure:* Brain activity  *Assessment method:* fMRI  *Function:* Etiologic  *Stimulus/task:* Social scenes (a person in pain and matched no-pain pictures) | Sexual sadists, relative to non-sadists, showed greater amygdala activation when viewing pain pictures. Sexual sadists, but not non-sadists, showed a positive correlation between pain severity ratings and activity in the anterior insula |
| Harris et al. (2012) | To determine the cues that control rapists’ erectile responses to rape stories in the laboratory | *Design:* Case-control study  *Country:* Canada  *Setting:* Inpatient clinic  *Follow-up:* NA | *N:* 12  *Gender:* M  *Age category:* Adult  *Diagnosis:* Not explicitly stated  *Offender type:* Sex offenders: Adult victims  *Controls:* M (14): Non-offenders | *Outcome measure:* Change in penis circumference  *Assessment method:* PPG  *Function:* Etiologic  *Stimulus/task:* Audio scenarios that systematically varied with regard to the presence or absence of three orthogonally varied elements: sexual activity and nudity, violence and injury, and expression of non-consent | Sexual interest in (or indifference to) non-consent is at least as central to accounting for the unique sexual orientation of rapists as is sexual responding to violence and injury |
| Herpertz et al. (2001) | To use multiple psychophysiological measures to compare emotional responses to unpleasant and pleasant stimuli | *Design:* Case-control study  *Country:* Germany  *Setting:* Inpatient clinic  *Follow-up:* NA | *N:* 43  *Gender:* M  *Age category:* Adult  *Diagnosis:* Mixed diagnoses  *Offender type:* Mixed offenders  *Controls:* M (44): Healthy controls | *Outcome measure:* Skin conductance, startle reflex, electromyographic activity  *Assessment method:* SCD, EMG, startle system  *Function:* Etiologic  *Stimulus/task:* Pleasant, unpleasant, and neutral slides | The results support the theory that psychopaths are characterized by a pronounced lack of fear in response to aversive events and suggest a general deficit in processing affective information, regardless of whether stimuli are negative or positive. Emotional hyporesponsiveness was specific to psychopaths |
| Hillbr et al. (1988) | To examine the absolute and relative strengths of the relationship between psychiatric diagnosis, electroencephalographic measures, psychological test data, social history data, and acts of severe violence | *Design:* Case-control study  *Country:* USA  *Setting:* Inpatient clinic  *Follow-up:* NA | *N:* 40  *Gender:* M  *Age category:* Adult  *Diagnosis:* Mixed diagnoses  *Offender type:* Violent offenders  *Controls:* M (45): Patients, non-violent | *Outcome measure:* Brain activity  *Assessment method:* EEG  *Function:* Etiologic  *Stimulus/task:* NA | A characterological type of dysphoria was found to have the strongest association with violence, followed by the absence of temporal lobe damage, and a passive style of response to threat |
| Hillbr et al. (1991) | The relationship between the frequency and severity of violence and three parameters of alcohol abuse was examined retrospectively in a sample of forensic psychiatric patients while EEG characteristics were also compared | *Design:* Case-control study  *Country:* USA  *Setting:* Inpatient clinic  *Follow-up:* NA | *N:* 60  *Gender:* M  *Age category:* Adult  *Diagnosis:* Substance use disorder  *Offender type:* Violent offenders  *Controls:* M (25): Forensic patients, non-abusers | *Outcome measure:* Brain activity  *Assessment method:* EEG  *Function:* Etiologic  *Stimulus/task:* NA | Acute alcohol abusers appeared to suffer from more severe central nervous system impairment |
| Hinton et al. (1980) | To compare psychophysiological characteristics of groups of security patients separated on some criteria of primary psychopathic behaviour and non-primary psychopathic behaviour, diagnosis of schizophrenia and psychopathy, and ratings of ward behaviour | *Design:* Case-control study  *Country:* UK  *Setting:* Inpatient clinic  *Follow-up:* NA | *N:* 35  *Gender:* M  *Age category:* Adult  *Diagnosis:* Psychopathy  *Offender type:* NS  *Controls:* M (7): Patients, schizophrenic, M (7): Patients, low risk | *Outcome measure:* Skin resistance, heart rate beat-to-beat variability, pupillary dilatation, electromyographic activity, muscle tension  *Assessment method:* Devices M19, six-channel polygraph and FM tape (videorecording pupil dilatation)  *Function:* Etiologic  *Stimulus/task:* Decision-making stress, tones, praise, criticism, a frustration situation, mental test performance, empathy test and passive noxious stimulation | Patients rated as disoriented by nurses and diagnosed schizophrenic tended to have increased spontaneous electrodermal fluctuations and reduced orienting response recovery time relative to non-disoriented and diagnosed psychopaths. Low rate spontaneous fluctuation in skin resistance plus long skin resistance differentiated ‘high public risk’ psychopaths from ‘low risk’ domestic offenders |
| Hoff et al. (2009) | To study emotional disability in psychopathy, this study compared fMRI-BOLD (blood oxygen level dependent) responses to healthy controls | *Design:* Case-control study  *Country:* Norway  *Setting:* Inpatient clinic  *Follow-up:* NA | *N:* 1  *Gender:* M  *Age category:* Adult  *Diagnosis:* Psychopathy  *Offender type:* Sex offenders: Child victims  *Controls:* M (6) - F (6): Healthy controls | *Outcome measure:* Brain activity  *Assessment method:* fMRI  *Function:* Etiologic  *Stimulus/task:* Drawings of facial expressions alternated with scrambled drawings | Exposure to facial expressions activated brain regions of older origin in the psychopath, whereas all activated regions in controls were neurocortical. This supports the notion that the processing of emotional stimuli in psychopathy is atypical |
| Hoppenbrouwers et al. (2013) | To assess inhibition and excitability directly from the left DLPFC in psychopathic offenders compared to healthy subjects whilst measuring working memory performance | *Design:* Case-control study  *Country:* Canada  *Setting:* Inpatient clinic  *Follow-up:* NA | *N:* 13  *Gender:* M  *Age category:* Adult  *Diagnosis:* Psychopathy  *Offender type:* NS  *Controls:* M (15): Healthy controls | *Outcome measure:* Brain activity  *Assessment method:* EEG  *Function:* Etiologic  *Stimulus/task:* Inhibition, excitability, and working memory task | Psychopathic offenders suffer from dysfunctional inhibitory neurotransmission in the DLPFC and impaired working memory which may account for the behavioral impairments associated with this disorder |
| Hoppenbrouwers et al. (2014) | To explore functional interhemispheric connectivity in psychopathic offenders using TMS and to provide the first neurophysiological data on interhemispheric (dys)function in individuals with psychopathy | *Design:* Case-control study  *Country:* Canada  *Setting:* Inpatient clinic  *Follow-up:* NA | *N:* 17  *Gender:* M  *Age category:* Adult  *Diagnosis:* Psychopathy  *Offender type:* NS  *Controls:* M (14): Healthy controls | *Outcome measure:* Brain activity  *Assessment method:* EEG  *Function:* Etiologic  *Stimulus/task:* Transcranial magnetic stimulation (TMS) | Global abnormalities in right-to-left functional connectivity were observed in psychopathic offenders compared with controls. Furthermore, psychopathic offenders showed increased intracortical inhibition in the right, but not the left, hemisphere |
| Hosking et al. (2017) | To test whether striatal hyperreactivity in psychopathic individuals is driven by a disruption in the cortical regulation of striatal information processing | *Design:* Cross-sectional study  *Country:* USA  *Setting:* Correctional facility  *Follow-up:* NA | *N:* 49  *Gender:* M  *Age category:* Adult  *Diagnosis:* Psychopathy  *Offender type:* NS  *Controls:* NA | *Outcome measure:* Brain activity  *Assessment method:* fMRI  *Function:* Etiologic  *Stimulus/task:* Delay-discounting task and resting state | Cortico-striatal circuit dysregulation drives maladaptive decision-making in psychopathy, supporting the notion that reward system dysfunction comprises an important neurobiological risk factor |
| Howard and McCullagh (2007) | To confirm neuroaffective processing deficits in psychopaths by measuring late brain event-related potential (ERP) components and behavior in groups of psychopathic and non-psychopathic inmates while they performed two tasks | *Design:* Case-control study  *Country:* UK  *Setting:* Correctional facility  *Follow-up:* NA | *N:* 17  *Gender:* NS  *Age category:* Adult  *Diagnosis:* Psychopathy  *Offender type:* NS  *Controls:* NS (17): Offenders, non-psychopathic | *Outcome measure:* ERP  *Assessment method:* EEG  *Function:* Etiologic  *Stimulus/task:* Categorization task, vigilance task | Results suggest that only under conditions of focused attention did psychopaths show a neuro-affective processing deficit and that their attentional focus was disrupted by the affective background |
| Howard et al. (1994) | To examine contingent negative variation (CNV) as a possible measure of sexual preference in child sex offenders | *Design:* Case-control study  *Country:* New-Zealand  *Setting:* Correctional facility  *Follow-up:* NA | *N:* 34  *Gender:* M  *Age category:* Adult  *Diagnosis:* Paedophilia  *Offender type:* Sex offenders: Child victims  *Controls:* M (19): Non-offenders | *Outcome measure:* Brain activity, change in penis circumference  *Assessment method:* EEG, PPG  *Function:* Diagnostic  *Stimulus/task:* EEG: match/mismatch paradigm using slides of nude male and female figures of different age categories; PPG: slides of males/females of several age categories, and a heterosexual intercourse video | It is concluded that CNV has promise as a measure of both deviant and non-deviant sexual preference |
| Howard and Lumsden (1996) | To assess a possible role for the Go/No Go contingent negative variation (CNV) as an indicator of subsequent reoffending by mentally disordered offenders released from a special high-security hospital | *Design:* Case-control study  *Country:* UK  *Setting:* Inpatient clinic  *Follow-up:* 15 years | *N:* 21  *Gender:* M  *Age category:* Adult  *Diagnosis:* Psychopathy, mental illness not otherwise specified  *Offender type:* NS  *Controls:* M (23): Released forensic patients, low risk | *Outcome measure:* Brain activity  *Assessment method:* EEG  *Function:* Prognostic  *Stimulus/task:* Go-NoGo task | Go/No Go CNV may be a useful predictor of recidivism in mentally disordered offenders |
| Howes (1998) | To test whether the significance of any deviance observed in the plethysmographic profiles of incarcerated rapists may be more validly determined if they are compared with the sexual arousal profiles of incarcerated non-sexual offenders rather than with the profiles of normal control groups | *Design:* Case-control study  *Country:* Canada  *Setting:* Correctional facility  *Follow-up:* NA | *N:* 50  *Gender:* M  *Age category:* Adult  *Diagnosis:* Paedophilia  *Offender type:* Sex offenders: Child and adult victims  *Controls:* M (50): Non-sex offenders | *Outcome measure:* Change in penis circumference  *Assessment method:* PPG  *Function:* Diagnostic  *Stimulus/task:* Visual erotic slides | The only marked difference between the two groups was in the almost-perfect ability of control participants to inhibit deviant arousal and the weak ability of sexual offenders to accomplish this. Determination of an offender's ability to inhibit deviant arousal may be the only aspect of plethysmographic testing which has practical application in the assessment of rapists |
| Howes (2009) | To compare the phallometric testing profiles of incarcerated sexual offenders with those of incarcerated non-sexual offenders | *Design:* Case-control study  *Country:* Canada  *Setting:* Correctional facility  *Follow-up:* NA | *N:* 50  *Gender:* M  *Age category:* Adult  *Diagnosis:* Paedophilia  *Offender type:* Sex offenders: Child and adult victims  *Controls:* M (50): Non-sex offenders | *Outcome measure:* Change in penis circumference  *Assessment method:* PPG  *Function:* Diagnostic  *Stimulus/task:* Audiovisual (deviant) sexual stimuli | Phallometric assessment offers a means of identifying any individual’s sexual preferences and a means of determining the extent to which he is able to control deviant arousal. Predictions of level of risk to engage in acts of sexual violence are rendered much more accurate by the inclusion of data from this technique |
| Howner (2010) | To investigate autonomic reactivity in response to emotional stimuli in mentally disordered offenders with various degrees of antisocial behaviour, but without psychopathy | *Design:* Case-control study  *Country:* Sweden  *Setting:* Correctional facility  *Follow-up:* NA | *N:* 41  *Gender:* M  *Age category:* Adult  *Diagnosis:* Antisocial personality disorder  *Offender type:* NS  *Controls:* M (20): Healthy controls | *Outcome measure:* Skin conductance  *Assessment method:* SCD  *Function:* Etiologic  *Stimulus/task:* Negative and neutral pictures | Differences in emotional response were found between the HC1 and the offender groups, but not between the two subgroups of offenders. Therefore, the results suggest that levels of antisocial traits may not be a differentiating factor with regard to emotional reactivity |
| Howner (2010) | To investigate the neural underpinning of emotional facial perception and whether this differs between psychopathic offenders and offenders with autism spectrum disorder, in comparison to a healthy control group | *Design:* Case-control study  *Country:* Sweden  *Setting:* Correctional facility  *Follow-up:* NA | *N:* 14  *Gender:* M  *Age category:* Adult  *Diagnosis:* Psychopathy  *Offender type:* NS  *Controls:* M (12): Healthy controls | *Outcome measure:* Brain activity  *Assessment method:* fMRI  *Function:* Etiologic  *Stimulus/task:* Fearful and neutral facial expressions | The whole offender group demonstrated increased neuronal (BOLD) activity compared to the HC2 group within the network involved in perceiving and processing emotional facial information |
| Ilyina et al. (2022) | To identify the characteristics of oculomotor activity in persons with paedophilic disorder under conditions of specific erotic visual stimulation | *Design:* Case-control study  *Country:* Russia  *Setting:* Correctional facility  *Follow-up:* NA | *N:* 10  *Gender:* M  *Age category:* Adult  *Diagnosis:* Paedophilia  *Offender type:* Sex offenders: Child victims  *Controls:* M (26): Healthy controls | *Outcome measure:* Eye movement  *Assessment method:* Eye tracker  *Function:* Etiologic  *Stimulus/task:* Photographs of women and children in everyday setting | Persons with paedophilia make a greater number of fixations on the image of a child, as well as the total duration of fixations when viewing this image is longer than in the control group groups |
| Iria et al. (2020) | To assess the performance and the arousal of antisocial offenders and controls in experimental tasks of identification of facial expressions of emotion using three contingencies: monetary reward, response cost, and no contingency | *Design:* Case-control study  *Country:* Portugal  *Setting:* Correctional facility  *Follow-up:* NA | *N:* 41  *Gender:* M  *Age category:* Adult  *Diagnosis:* Antisocial personality disorder  *Offender type:* NS  *Controls:* M (40): Healthy controls | *Outcome measure:* Skin conductance  *Assessment method:* Polygraph  *Function:* Etiologic  *Stimulus/task:* Pictures of facial expressions and reward, response cost and no contingency condition | The offenders with ASP presented higher SCR than the controls in the two monetary conditions. Findings suggest that offenders with ASP are hypersensitive to monetary contingencies; monetary reward seems to interfere negatively with their performance while monetary response cost improves it |
| Jankovic et al. (2021) | To investigate the complex associations between early adversity, heart rate variability, cluster B personality disorders, and self-reported aggressive behavior in a group of male forensic inpatients | *Design:* Cohort study  *Country:* The Netherlands  *Setting:* Inpatient clinic  *Follow-up:* 16 weeks | *N:* 50  *Gender:* M  *Age category:* Adult  *Diagnosis:* Personality disorder  *Offender type:* NS  *Controls:* NA | *Outcome measure:* Brain activity, heart rate variability  *Assessment method:* EEG, ECG  *Function:* Etiologic  *Stimulus/task:* VR Training | Patients with cluster B personality disorders were more likely to have adverse early childhood experiences and reduced sympathetic dominance in response to a threat than patients without cluster B personality disorders. HRV and cluster B personality disorders did not significantly mediate the association between early childhood adversity and self-reported aggressive behavior |
| Jiang et al. (2013) | To examine whether persons with ASPD show differences in brain activities between lying and truth-telling | *Design:* Cross-sectional study  *Country:* China  *Setting:* Correctional facility  *Follow-up:* NA | *N:* 32  *Gender:* NS  *Age category:* Adult  *Diagnosis:* Antisocial personality disorder  *Offender type:* Violent offenders  *Controls:* NA | *Outcome measure:* Brain activity  *Assessment method:* fMRI  *Function:* Etiologic  *Stimulus/task:* Truth and lie telling task with pictures | Truthful and untruthful communications of ASPD subjects can be differentiated in terms of brain BOLD activities |
| Jiang et al. (2017) | To investigate the topological architecture of intrinsic brain networks in patients with ASPD | *Design:* Case-control study  *Country:* China  *Setting:* Correctional facility  *Follow-up:* NA | *N:* 32  *Gender:* NS  *Age category:* Adult  *Diagnosis:* Antisocial personality disorder  *Offender type:* Violent offenders  *Controls:* NS (35): Offenders without the condition | *Outcome measure:* Brain activity  *Assessment method:* fMRI  *Function:* Etiologic  *Stimulus/task:* NA (resting-state fMRI) | ASPD is associated with both reduced brain integration and segregation in topological organization of functional brain networks, particularly in the fronto-parietal control network. These disruptions may contribute to disturbances in behavior and cognition in patients with ASPD |
| Jones et al. (2018) | To explore the neural correlates of empathy in juveniles who sexually offend, and the possible role of child sexual abuse | *Design:* Case-control study  *Country:* USA  *Setting:* Inpatient clinic  *Follow-up:* NA | *N:* 27  *Gender:* M  *Age category:* Juvenile  *Diagnosis:* Mixed diagnoses  *Offender type:* Sex offenders: Victim not specified  *Controls:* M (11): Healthy controls | *Outcome measure:* Brain activity  *Assessment method:* fMRI  *Function:* Etiologic  *Stimulus/task:* View images with different instructions: watch condition, empathize condition, and cognitive load/memorize condition | fMRI data showed no statistical differences in engagement of brain regions associated with empathy between controls and all juvenile sex offenders (JSOs). There were no significant differences between JSOs with and without a history of child sexual abuse |
| Jones et al. (2017) | To compare juveniles who sexually offend to non-offending juveniles in their capacities to behaviorally and neurologically regulate, or reappraise, negative emotions | *Design:* Case-control study  *Country:* USA  *Setting:* Inpatient clinic and outpatient clinic  *Follow-up:* NA | *N:* 29  *Gender:* M  *Age category:* Juvenile  *Diagnosis:* Mixed diagnoses  *Offender type:* Sex offenders: Victim not specified  *Controls:* M (11): Non-offenders | *Outcome measure:* Brain activity  *Assessment method:* fMRI  *Function:* Etiologic  *Stimulus/task:* Attend and reappraisal task viewing different neutral and negative images | Findings suggest that juveniles who sexually offend are capable of emotion regulation |
| Jordan et al. (2014) | To examine the potential of the eye tracking method and the fMRI measure to explore the impact of testosterone suppression to castration level on automatic and controlled attentional processes | *Design:* Case study  *Country:* Germany  *Setting:* Inpatient clinic  *Follow-up:* 4 months | *N:* 1  *Gender:* M  *Age category:* Adult  *Diagnosis:* Paedophilia  *Offender type:* Sex offenders: Child victims  *Controls:* NA | *Outcome measure:* Brain activity, eye movement  *Assessment method:* fMRI, eye tracker  *Function:* Monitoring  *Stimulus/task:* Attend and reappraisal task viewing different neutral and negative images | The experimental designs using eye tracking and fMRI could prospectively add additional and valuable information in the evaluation of ADT in paraphilic patients and sex offenders |
| Jordan et al. (2018) | To compare people with a self-reported sexual interest in children who participate in the outpatient preventive treatment project, paedophilic forensic inpatients, and a non-paedophilic control group | *Design:* Case-control study  *Country:* Germany  *Setting:* Inpatient clinic and outpatient clinic  *Follow-up:* NA | *N:* 33  *Gender:* M  *Age category:* Adult  *Diagnosis:* Paedophilia  *Offender type:* Sex offenders: Child victims  *Controls:* M (8): Inpatients, M (52): Healthy controls | *Outcome measure:* Eye movement  *Assessment method:* Eye tracker  *Function:* Etiologic  *Stimulus/task:* Mental Rotation Stimuli and Sexual Distractors | Outpatients with a self-reported sexual interest in children differed from paedophilic forensic inpatients with respect to attentional control but not with regard to sexual interest. They demonstrated significantly better attentional control than paedophilic forensic inpatients in the face of adult sexual stimuli, but the difference regarding child sexual stimuli did not reach significance |
| Jordan et al. (2016) | To investigate how early (fixation latency) and late (relative fixation time) attentional processes were allocated to the cognitive target stimuli and the sexual distractors | *Design:* Case-control study  *Country:* Germany  *Setting:* Inpatient clinic  *Follow-up:* NA | *N:* 22  *Gender:* M  *Age category:* Adult  *Diagnosis:* Paedophilia  *Offender type:* Sex offenders: Child victims  *Controls:* M (7): Inpatients, M (50): Healthy controls | *Outcome measure:* Eye movement  *Assessment method:* Eye tracker  *Function:* Etiologic  *Stimulus/task:* Mental Rotation Stimuli and Sexual Distractors | Paedophiles demonstrated significantly lower attentional control in the sexual distractor task than both control groups. They showed a shorter fixation latency and longer fixation time for sexual distractors than non-paedophiles and demonstrated a longer fixation latency and shorter fixation time for cognitive target stimuli |
| Jordan et al. (2016) | To measure sexual interest under cognitive load in paedophiles, forensic control patients, and healthy subjects | *Design:* Case-control study  *Country:* Germany  *Setting:* Inpatient clinic  *Follow-up:* NA | *N:* 22  *Gender:* M  *Age category:* Adult  *Diagnosis:* Paedophilia  *Offender type:* Sex offenders: Child victims  *Controls:* M (7): Inpatients without any history of sexual assault against children, M (50): Healthy controls | *Outcome measure:* Eye movement  *Assessment method:* Eye tracker  *Function:* Etiologic  *Stimulus/task:* Mental Rotation Stimuli and Sexual Distractors | Controls showed an impairment of cognitive performance when sexually relevant distractors were presented. They took significantly more time to look at sexual adult distractors than at sexual child distractors. Both forensic groups performed much poorer than the healthy control group without specificity for certain sexual distractors. While forensic control subjects tended to view adult stimuli longer than those of children, no differences were found for paedophiles |
| Joyal et al. (2007) | To verify whether violent persons with schizophrenia and the co-morbid diagnoses of an antisocial personality disorder and a substance use disorder present a different pattern of prefrontal functioning than seriously violent persons with schizophrenia only | *Design:* Case-control study  *Country:* Finland  *Setting:* Correctional facility  *Follow-up:* NA | *N:* 24  *Gender:* M  *Age category:* Adult  *Diagnosis:* Psychotic disorder  *Offender type:* Violent offenders  *Controls:* M (12): Non-offenders | *Outcome measure:* Brain activity  *Assessment method:* fMRI  *Function:* Etiologic  *Stimulus/task:* Go-NoGo task | Frontal basal cortices were significantly less activated in persons with Sz+APD+SUD during the execution of a Go/No-go task than in persons with Sz only and non-violent persons without a mental illness. Significantly higher activations in frontal motor, premotor and anterior cingulate regions were observed in the Sz+APD+SUD group than in the Sz-only group |
| Juárez et al. (2013) | To examine paralimbic connectivity in adult incarcerated individuals stratified by psychopathy scores | *Design:* Case-control study  *Country:* USA  *Setting:* Correctional facility  *Follow-up:* NA | *N:* 54  *Gender:* NS  *Age category:* Adult  *Diagnosis:* Psychopathy  *Offender type:* NS  *Controls:* NS (48): Offenders, low psychopathy | *Outcome measure:* Brain activity  *Assessment method:* fMRI  *Function:* Etiologic  *Stimulus/task:* Auditory target detection oddball task | Consistent with the hypothesis of limbic/paralimbic abnormalities associated with psychopathy, modulation trends correlated strongly with PCL-R scores. There is strong evidence to implicate the posterior cingulate in aberrant functional connectivity associated with the manifestation of psychopathic symptoms |
| Jutai et al. (1987) | To determine whether low left-hemisphere arousal or unusual cortical responses to speech stimuli might be associated with anomalies in language function that reportedly occur when psychopaths perform lateralized information-processing tasks | *Design:* Case-control study  *Country:* Canada  *Setting:* Correctional facility  *Follow-up:* NA | *N:* 11  *Gender:* M  *Age category:* Adult  *Diagnosis:* Psychopathy  *Offender type:* NS  *Controls:* M (13): Offenders, non-psychopathic | *Outcome measure:* ERP, skin conductance, eye movement  *Assessment method:* EEG, EOG, Beckman Type 611 polygraph  *Function:* Etiologic  *Stimulus/task:* Speech stimuli | The results were consistent with the hypothesis that psychopaths have limited left-hemisphere resources for processing linguistic stimuli |
| Jutai and Hare (1983) | To investigate selective attention during performance of a complex perceptual-motor task in psychopathic offenders | *Design:* Case-control study  *Country:* Canada  *Setting:* Correctional facility  *Follow-up:* NA | *N:* 11  *Gender:* M  *Age category:* Adult  *Diagnosis:* Psychopathy, antisocial personality disorder  *Offender type:* NS  *Controls:* M (10): Offenders, low psychopathy | *Outcome measure:* Brain activity, skin conductance, eye movement, heart rate  *Assessment method:* Polygraph (Beckman type 411): Skin conductance coupler, cardiotachometer, EOG (electrooculogram), EEG  *Function:* Etiologic  *Stimulus/task:* Complex perceptual-motor task (playing a video game) + tone pips (irrelevant stimuli) | Results provided some support for the hypothesis that psychopaths allocate a relatively large proportion of their attentional resources to things of immediate interest, effectively ignoring other stimuli |
| Kaine et al. (1988) | To examine the ability to suppress, fake positive or otherwise distort penile responses in sex preference | *Design:* Case-control study  *Country:* Canada  *Setting:* Correctional facility  *Follow-up:* NA | *N:* 10  *Gender:* NS  *Age category:* Adult  *Diagnosis:* Paedophilia  *Offender type:* Sex offenders: Child and adult victims  *Controls:* NS (10): Non-sex offenders | *Outcome measure:* Change in penis circumference  *Assessment method:* PPG  *Function:* Diagnostic  *Stimulus/task:* Visual and audiovisual stimuli depicting male and female models at various ages and degrees of sexual development | A clear ability to suppress penile responses to preferred stimuli is demonstrated in both sex offender and other (non-sex) offender groups. No clear ability to "fake" penile arousal to a non-preferred stimulus was demonstrated |
| Kamenskov (2013) | To analyze the experimental-practical validity of a psychophysiological method (polygraph testing) in the diagnosis of paraphilia | *Design:* Case-control study  *Country:* Serbia  *Setting:* NS  *Follow-up:* NA | *N:* 69  *Gender:* NS  *Age category:* Adult  *Diagnosis:* Paedophilia  *Offender type:* Sex offenders: Child and adult victims  *Controls:* NS (44): Offenders without the condition | *Outcome measure:* Galvanic skin reflex, cardiovascular reactions, thoracic and diaphragmatic breathing, muscle activity, speech reactions  *Assessment method:* Polygraph  *Function:* Diagnostic  *Stimulus/task:* Verbal and visual sexual stimulation | Results of verbal and visual psychophysiological stimulation allowed to diagnose a sexual disorder in people with paraphilia in 80% and 69.6% of the cases, respectively. The experimental-practical validity of the psychophysiological diagnosis was 0.63 that corresponded to the high level of validity |
| Kärgel et al. (2017) | To disentangle behavioral and neural characteristics of response inhibition processes associated with paedophilia or child sexual offending | *Design:* Case-control study  *Country:* Germany  *Setting:* Correctional facility  *Follow-up:* NA | *N:* 77  *Gender:* M  *Age category:* Adult  *Diagnosis:* Paedophilia  *Offender type:* Sex offenders: Child victims  *Controls:* M (40): Healthy controls | *Outcome measure:* Brain activity  *Assessment method:* fMRI  *Function:* Etiologic  *Stimulus/task:* Go-NoGo task | Heightened inhibition-related recruitment of the left posterior cingulate and the left superior frontal cortexas well as decreased amount of commission errors is related to better inhibitory control in paedophiles who successfully avoid committing hands-on sexual offenses against children |
| Kärgel et al. (2015) | To provide new insights in how functional integration of brain regions may relate to paedophilia or child sexual abuse | *Design:* Case-control study  *Country:* Germany  *Setting:* Correctional facility  *Follow-up:* NA | *N:* 26  *Gender:* M  *Age category:* Adult  *Diagnosis:* Paedophilia  *Offender type:* Sex offenders: Child victims  *Controls:* M (14): Healthy controls | *Outcome measure:* Brain activity  *Assessment method:* fMRI  *Function:* Etiologic  *Stimulus/task:* NA (resting-state fMRI) | Diminished resting state functional connectivity was found in brain networks critically involved in widespread motivational and socio-emotional processes |
| Kennard et al. (1955) | To compare the EEGs and wave-frequencies in groups of subjects which were thought to show marked differences in EEG patterns and to seek psychological characteristics that might be related to these patterns | *Design:* Case-control study  *Country:* Canada  *Setting:* Correctional facility  *Follow-up:* NA | *N:* 31  *Gender:* M (NS) + F (NS)  *Age category:* Adult and juvenile  *Diagnosis:* Psychopathy  *Offender type:* NS  *Controls:* M/F (50): Healthy controls, M/F: Schizophrenic patients | *Outcome measure:* Brain activity  *Assessment method:* EEG  *Function:* Etiologic  *Stimulus/task:* NA (resting-state EEG) | It is clear that many slight and subtle pattern differences may be more clearly and more objectively seen by the use of frequency analysis than by means of the usual scanning with the eye |
| Kiehl (2000) | To determine if the frontocentral ERP negativities previously observed in psychopaths during language tasks are due to abnormalities in semantic processes (i.e., N4) and/or in combination with abnormalities in attentional/contextual updating processes (i.e., P3) | *Design:* Case-control study  *Country:* Canada  *Setting:* Inpatient clinic  *Follow-up:* NA | *N:* 11  *Gender:* M  *Age category:* Adult  *Diagnosis:* Psychopathy  *Offender type:* Mixed offender types  *Controls:* M (10): Offenders, non-psychopathic | *Outcome measure:* ERP  *Assessment method:* EEG  *Function:* Etiologic  *Stimulus/task:* Visual stimuli | The P300 response to target stimuli was smaller in psychopaths than in non-psychopaths consistent with the hypothesis that psychopathy is associated with an impaired ability to allocate attentional resources |
| Kiehl (2000) | To determine if the frontocentral ERP negativities previously observed in psychopaths during language tasks are due to abnormalities in semantic processes (i.e., N4) and/or in combination with abnormalities in attentional/contextual updating processes (i.e., P3) | *Design:* Case-control study  *Country:* Canada  *Setting:* Inpatient clinic  *Follow-up:* NA | *N:* 11  *Gender:* M  *Age category:* Adult  *Diagnosis:* Psychopathy  *Offender type:* Mixed offender types  *Controls:* M (10): Offenders, non-psychopathic | *Outcome measure:* ERP  *Assessment method:* EEG  *Function:* Etiologic  *Stimulus/task:* Visual stimuli | The P300 response to target stimuli was smaller in psychopaths than in non-psychopaths consistent with the hypothesis that psychopathy is associated with an impaired ability to allocate attentional resources |
| Kiehl (2000) | To examine the integrity of the neural systems underlying semantic processes in the absence of concurrent task demands to attempt to isolate and characterize the conditions in which large frontocentral negativities are elicited | *Design:* Case-control study  *Country:* Canada  *Setting:* Correctional facility  *Follow-up:* NA | *N:* 25  *Gender:* M  *Age category:* Adult  *Diagnosis:* Psychopathy  *Offender type:* NS  *Controls:* M (25): Offenders, non-psychopathic | *Outcome measure:* ERP, eye movement  *Assessment method:* EEG, EOG  *Function:* Etiologic  *Stimulus/task:* Sentence processing paradigm | No group differences were observed between psychopaths and non-psychopaths in the amplitude of the N400 potential elicited by terminal words of sentences that were either congruent or incongruent with the previous sentence context |
| Kiehl et al. (2006) | To examine the neural systems underlying processes of attention and orienting in psychopaths | *Design:* Case-control study  *Country:* Canada  *Setting:* Correctional facility  *Follow-up:* NA | *N:* Sample 1: 23; Sample 2: 18  *Gender:* M  *Age category:* Adult  *Diagnosis:* Psychopathy  *Offender type:* NS  *Controls:* M (Sample 1: 21, Sample 2: 18): Offenders, non-psychopathic | *Outcome measure:* ERP, eye movement  *Assessment method:* EEG, EOG  *Function:* Etiologic  *Stimulus/task:* Auditory oddball task | The data support the hypothesis that psychopathy may be related to dysfunction of the paralimbic system, a system that includes parts of the temporal and frontal lobes |
| Kiehl et al. (1999) | To elucidate and characterize the neural correlates of cognitive processes of psychopaths | *Design:* Case-control study  *Country:* Canada  *Setting:* Inpatient clinic  *Follow-up:* NA | *N:* 11  *Gender:* M  *Age category:* Adult  *Diagnosis:* Psychopathy  *Offender type:* Mixed offender types  *Controls:* M (10): Offenders, non-psychopathic | *Outcome measure:* Brain activity  *Assessment method:* EEG  *Function:* Etiologic  *Stimulus/task:* Visual oddball task | There are substantial differences between psychopaths and others in the processing of even simple cognitive tasks and provide support for information processing models of psychopathy |
| Kiehl et al. (1999) | To test the hypothesis that psychopathy is associated with abnormal processing of semantic and affective verbal information | *Design:* Case-control study  *Country:* Canada  *Setting:* Inpatient clinic  *Follow-up:* NA | *N:* 8  *Gender:* M  *Age category:* Adult  *Diagnosis:* Psychopathy  *Offender type:* Mixed offender types  *Controls:* M (12): Offenders, mixed psychopathy group, M (9): Offenders, non-psychopathic | *Outcome measure:* ERP  *Assessment method:* EEG  *Function:* Etiologic  *Stimulus/task:* Lexical decision task and identification task | Non-psychopaths showed the expected event-related potential ERP differentiation between word stimuli, whereas psychopaths did not. In each task, the ERPs of the psychopaths included a large centrofrontal negative-going wave N350; which was absent or very small in the non-psychopaths |
| Kiehl et al. (2006) | To examine the integrity of the neural systems underlying semantic processes in the absence of concurrent task demands to attempt to isolate and characterize the conditions in which large frontocentral negativities are elicited and to determine whether the abnormalities observed in psychopaths during semantic language tasks are related to the processes known to elicit the N400 during sentence processing tasks | *Design:* Case-control study  *Country:* Canada  *Setting:* Correctional facility  *Follow-up:* NA | *N:* 25  *Gender:* M  *Age category:* Adult  *Diagnosis:* Psychopathy  *Offender type:* NS  *Controls:* M (25): Offenders, non-psychopathic | *Outcome measure:* ERP, eye movement  *Assessment method:* EEG, EOG  *Function:* Etiologic  *Stimulus/task:* Canonical semantic sentence processing paradigm | The data do not support the hypothesis that the semantic processes, and underlying neural systems, associated with the generation of the N400 during sentence processing tasks are abnormal in psychopathy |
| Kiehl et al. (2000) | To test the hypothesis that schizophrenia and psychopathy are associated with abnormal neural processing during the suppression of inappropriate responses | *Design:* Case-control study  *Country:* Canada  *Setting:* Inpatient clinic  *Follow-up:* NA | *N:* 25  *Gender:* M  *Age category:* Adult  *Diagnosis:* Psychopathy  *Offender type:* Mixed offender types  *Controls:* M (11): Patients, non-psychopathic | *Outcome measure:* ERP  *Assessment method:* EEG  *Function:* Etiologic  *Stimulus/task:* Go-NoGo task | Results support the hypothesis that the neural processes involved in response inhibition are abnormal in both schizophrenia and psychopathy; however, the nature of these processes appears to be different in the two disorders |
| Kiehl et al. (2001) | fMRI was used to elucidate the neurobiological correlates of the anomalies in criminal psychopaths during performance of an affective memory task | *Design:* Case-control study  *Country:* Canada  *Setting:* Correctional facility  *Follow-up:* NA | *N:* 8  *Gender:* NS  *Age category:* Adult  *Diagnosis:* Psychopathy  *Offender type:* NS  *Controls:* NS (8): Offenders, non-psychopathic, NS (8): Healthy controls | *Outcome measure:* Brain activity  *Assessment method:* fMRI  *Function:* Etiologic  *Stimulus/task:* Affective memory task | Results suggest that the affective abnormalities so often observed in psychopathic offenders may be linked to deficient or weakened input from limbic structures |
| Kiehl et al. (2004) | To elucidate the neural architecture underlying lexicosemantic processing in criminal psychopathic individuals during performance of a concrete/abstract lexical decision task | *Design:* Case-control study  *Country:* Canada  *Setting:* Correctional facility  *Follow-up:* NA | *N:* 8  *Gender:* M  *Age category:* Adult  *Diagnosis:* Psychopathy  *Offender type:* NS  *Controls:* M (8): Healthy controls | *Outcome measure:* Brain activity  *Assessment method:* fMRI  *Function:* Etiologic  *Stimulus/task:* Lexical decision task | Results support the theory that psychopathy is associated with right hemisphere abnormalities for processing conceptually abstract material |
| Kimonis et al. (2017) | To differentiate primary and secondary variants of juvenile psychopathy with affective startle potentiation | *Design:* Case-control study  *Country:* USA  *Setting:* Correctional facility  *Follow-up:* NA | *N:* 46  *Gender:* M  *Age category:* Juvenile  *Diagnosis:* Juvenile psychopathic traits  *Offender type:* NS  *Controls:* M (42): Offenders, secondary psychopathy, M (37): Offenders, maltreatment, M (111): Offenders, non-maltreatment | *Outcome measure:* Fear-potentiated startle  *Assessment method:* EMG  *Function:* Etiologic  *Stimulus/task:* Aversive, pleasant and neutral pictures | Primary CU variants displayed reduced startle potentiation to aversive images relative to control, maltreated, and also secondary variants that exhibited greater startle modulation |
| Kingston et al. (2007) | To examine the utility of the diagnosis of paedophilia in a sample of extra-familial child molesters | *Design:* Cross-sectional study  *Country:* Canada  *Setting:* Outpatient clinic  *Follow-up:* NA | *N:* 85 (DSM-diagnosis), 110 (Deviant phallometric index score), 49 (DSM-diagnosis + deviant phallometric index score), 103 (Paedophilic based on the SSPI-score)  *Gender:* M  *Age category:* Adult  *Diagnosis:* Paedophilia  *Offender type:* Sex offenders: Victim not specified  *Controls:* M (79): Offenders, no diagnosis, M (45): Offenders, no deviant phallometric index score, M (43): Offenders, no diagnosis and no deviant phallometric index score, M (103): Offenders, not paedophilic based on the SSPI-score | *Outcome measure:* Change in penis circumference  *Assessment method:* PPG  *Function:* Diagnostic  *Stimulus/task:* Audio (erotic/sexual) stimuli | Few significant differences existed in psychological measures between paedophilic and non-paedophilic extra-familial child molesters regardless of the classification system employed |
| Kirenskaya and Kamenskov (2019) | To provide a comparison of how the prefrontal cortex functioning using AS performance and CNV analysis in patients with disorders of sexual interest (paraphilia) differed in the severity of self-consciousness impairments | *Design:* Case-control study  *Country:* Russia  *Setting:* Outpatient clinic  *Follow-up:* NA | *N:* 42  *Gender:* M  *Age category:* Adult  *Diagnosis:* Paraphilia  *Offender type:* Sex offenders: Victim not specified  *Controls:* M (23): Healthy controls | *Outcome measure:* Brain activity, eye movement  *Assessment method:* EEG, EOG  *Function:* Etiologic  *Stimulus/task:* Visual stimuli (led light) | The study found a decreased level of frontal cortex activation in subjects with disorders of self-consciousness; in subjects without disturbances of self-consciousness a high level of frontal cortex activation was observed |
| Kirenskaya et al. (2013) | To test whether patients with stereotyped paraphilia had frontal cortex impairment using AS performance and CNV analysis and to compare AS performance and CNV characteristics in patients with stereotyped paraphilia and schizophrenia | *Design:* Case-control study  *Country:* Russia  *Setting:* Outpatient clinic  *Follow-up:* NA | *N:* 12  *Gender:* M  *Age category:* Adult  *Diagnosis:* Paraphilia  *Offender type:* Sex offenders: Victim not specified  *Controls:* M (16): Schizophrenic, M (19): Healthy controls | *Outcome measure:* Brain activity, eye movement  *Assessment method:* EEG, EOG  *Function:* Etiologic  *Stimulus/task:* Visual stimuli (led light) | Two distinct types of contingent negative variation abnormalities were found. The schizophrenic patient's results suggest frontal dysfunction in schizophrenia |
| Kirenskaya and Tkachenko (2003) | To study neurophysiological mechanisms of abnormal sexual behavior in persons with organic mental disorders with the application of EEG spectral analysis in the state of rest and during functional loads | *Design:* Case-control study  *Country:* Russia  *Setting:* Outpatient clinic  *Follow-up:* NA | *N:* 19  *Gender:* M  *Age category:* Adult  *Diagnosis:* Paraphilia  *Offender type:* Sex offenders: Victim not specified  *Controls:* M (21): Patients, no paraphilia, M (19): Healthy controls | *Outcome measure:* Brain activity  *Assessment method:* EEG  *Function:* Etiologic  *Stimulus/task:* NA (resting-state EEG) | The results suggest the formation of a stationary activation focus in the right hemisphere with signs of involvement of the limbic structures in patients with paraphilias |
| Klapwijk et al. (2016) | To compare the neural correlates of cognitive and affective aspects of empathy between youth with ASD and youth with CD/CU+ | *Design:* Case-control study  *Country:* The Netherlands  *Setting:* Correctional facility and inpatient clinic  *Follow-up:* 1 week | *N:* 32  *Gender:* M  *Age category:* Juvenile  *Diagnosis:* Conduct disorder  *Offender type:* Violent offenders  *Controls:* M (33): Healthy controls | *Outcome measure:* Brain activity  *Assessment method:* fMRI  *Function:* Etiologic  *Stimulus/task:* Written emotional responses (angry, disappointed or happy) | Boys with CD have difficulties with processing explicit emotional cues from others on behavioral and neural levels |
| Klapwijk et al. (2016) | To compare the neural correlates of cognitive and affective aspects of empathy between youth with ASD and youth with CD/CU+ | *Design*: Case-control study  *Country:* The Netherlands  *Setting:* Correctional facility, inpatient clinic, and outpatient clinic  *Follow-up:* NA | *N:* 23 (ASD), 23 (CD/CU)  *Gender:* M  *Age category:* Juvenile  *Diagnosis:* Psychopathy  *Offender type:* Violent offenders  *Controls:* M (33): Healthy controls | *Outcome measure:* Brain activity  *Assessment method:* fMRI  *Function:* Etiologic  *Stimulus/task:* Angry and fearful faces | Results suggest differential abnormal brain responses associated with specific aspects of empathic functioning in ASD and CD/CU+. Decreased amygdala responses in ASD and CD/CU+ suggest impaired emotion processing. Reduced vmPFC responses suggest problems in processing cognitive aspects of empathy in ASD. Reduced IFG/AI responses suggest decreased emotional resonance in CD/CU+ |
| Klein (2005) | To examine whether differences exist in persons with developmental disabilities who have committed either an offense against a child or an offense against an adult | *Design*: Cross-sectional study  *Country:* USA  *Setting:* Inpatient clinic and outpatient clinic  *Follow-up:* NA | *N:* 42  *Gender:* M  *Age category:* Adult  *Diagnosis:* Other  *Offender type:* Sex offenders: Child and adult victims  *Controls:* NA | *Outcome measure:* Change in penis circumference  *Assessment method:* PPG  *Function:* Etiologic  *Stimulus/task:* Audio and visual (erotic/sexual) stimuli | Certain differences may exist between developmentally disabled offenders against adult women and developmentally disabled offenders against children with regard to deviant arousal, which is in opposition to the hypothesis proposed |
| Knott et al. (2016) | To investigate the time course of the explicit processing of erotic, emotional, and neutral pictures in paedophilic patients and healthy controls with event-related brain potentials (ERP) | *Design*: Case-control study  *Country:* Canada  *Setting:* Outpatient clinic  *Follow-up:* NA | *N:* 22  *Gender:* M  *Age category:* Adult  *Diagnosis:* Paedophilia  *Offender type:* Sex offenders: Child victims  *Controls:* M (20): Healthy controls | *Outcome measure:* Brain activity  *Assessment method:* EEG  *Function:* Etiologic  *Stimulus/task:* Erotic, negative, positive, and neutral visual stimuli (pictures) | Failure of rapid attentional capture by erotic stimuli suggests a relative reduction in early processing in paedophilic patients which may be associated with relatively diminished sexual interest in adults |
| Kolarsky and Madlafousek (1972) | Two questions were examined: Are sexual deviates sexually arousable by female courtship behavior as normal men are and are these deviates sexually inhibited by female non-erotic activity as normal men appear to be? | *Design*: Case-control study  *Country:* Czechoslovakia  *Setting:* Outpatient clinic  *Follow-up:* NA | *N:* 15  *Gender:* M  *Age category:* Adult and juvenile  *Diagnosis:* Paraphilia  *Offender type:* Sex offenders: Adult victims  *Controls:* M (15): Healthy controls | *Outcome measure:* Change in penis circumference  *Assessment method:* PPG  *Function:* Etiologic  *Stimulus/task:* Film scenes of a female actress | In contrast to normal men deviates are not sexually inhibited by female non-erotic movements. Deviates were found sexually arousable by courtship movements just as normal men were |
| Kolarsky et al. (1978) | To compare the phm responses of normals and deviates to a number of film scenes showing another actress, this time nude, exhibiting behavior with different kinds of 'seductive' cues | *Design*: Case-control study  *Country:* Czechoslovakia  *Setting:* NS  *Follow-up:* NA | *N:* 14  *Gender:* M  *Age category:* Adult  *Diagnosis:* Paraphilia  *Offender type:* Sex offenders: Adult victims  *Controls:* M (14): Healthy controls | *Outcome measure:* Change in penis circumference  *Assessment method:* PPG  *Function:* Diagnostic  *Stimulus/task:* Short film scenes showing a naked actress's seductive behaviour | Deviates responded positively to the scenes and differentiated strong and weak seduction scenes similarly to normals |
| Kolla et al. (2016) | Because it is currently unknown whether phenotypic MAO-A markers can influence brain function in ASPD, this study investigated VS MAO-A level and the functional connectivity (FC) of two seed regions, superior and inferior VS | *Design*: Cross-sectional study  *Country:* Canada  *Setting:* Correctional facility  *Follow-up:* NA | *N:* 19  *Gender:* M  *Age category:* Adult  *Diagnosis:* Personality disorder  *Offender type:* Mixed offender types  *Controls:* NA | *Outcome measure:* Brain activity  *Assessment method:* fMRI  *Function:* Etiologic  *Stimulus/task:* NA (resting-state fMRI) | Results highlight an association of VS MAO-A level with the FC of striatal regions linked to impulsive behavior in ASPD and suggest that phenotype-based brain markers of ASPD have relevance to understanding brain function |
| Konicar et al. (2021) | To investigate the central nervous and autonomic peripheral changes occurring after brain self-regulation in a group of severe male offenders with psychopathy | *Design*: Cohort study  *Country:* Germany  *Setting:* Inpatient clinic  *Follow-up:* 38 days | *N:* 14  *Gender:* M  *Age category:* Adult  *Diagnosis:* Psychopathy  *Offender type:* Mixed offender types  *Controls:* NA | *Outcome measure:* Brain activity, electrodermal activity  *Assessment method:* EEG, EDA-sensor  *Function:* Intervention  *Stimulus/task:* Neurofeedback | Regarding the central nervous system, an overall suppression of the psychopathic overrepresentation of slow frequency bands was found, such as delta and theta band activity, after EEG neurofeedback. An increase in alpha band activity could be observed after the SCP self-regulation training. Electrodermal activity adaptively changed according to the regulation task, and this flexibility improved over training time |
| Konopasek (2015) | To explore whether the variable of polygraph-facilitated sexual history disclosure and the timeliness of such disclosure is related to treatment outcome and sexual recidivism | *Design*: Cohort study  *Country:* USA  *Setting:* Outpatient clinic  *Follow-up:* Within 12 months of evaluation/treatment intake | *N:* 192  *Gender:* M (182) + F (10)  *Age category:* Adult  *Diagnosis:* Paraphilia  *Offender type:* Sex offenders: Child and adult victims  *Controls:* NA | *Outcome measure:* Physiological indicators NOS  *Assessment method:* Polygraph  *Function:* Predictive  *Stimulus/task:* NA | Results, though revealing no relationship between polygraph-facilitated expeditious disclosure and sexual recidivism, did present preliminary findings that such disclosure efforts may facilitate treatment completion |
| Korponay et al. (2017) | To determine whether volumes of striatal subregions are linked to assessments of overall psychopathy severity as well as to assessments of distinct components of psychopathic traits and whether the observed striatal structural abnormalities are accompanied by alterations in striatal functional connectivity | *Design*: Case-control study  *Country:* USA  *Setting:* Correctional facility  *Follow-up:* NA | *N:* 41  *Gender:* M  *Age category:* Adult  *Diagnosis:* Psychopathy  *Offender type:* NS  *Controls:* M (48): Offenders, intermediate psychopathy, M (35): Offenders, non-psychopathic | *Outcome measure:* Brain activity  *Assessment method:* fMRI  *Function:* Etiologic  *Stimulus/task:* NA (resting-state fMRI) | Findings associate the impulsive/antisocial dimension of psychopathy with enlarged striatal subnuclei and aberrant functional connectivity between the striatum and other brain regions. Co-localization of volumetric and functional connectivity findings suggests that these neural abnormalities may be pathophysiologically linked |
| Krusemark et al. (2016) | To examine whether participants with high psychopathy scores would exhibit an exaggerated N2pc relative to non-psychopathic participants in response to set-congruent cues during the color task | *Design*: Case-control study  *Country:* USA  *Setting:* Correctional facility  *Follow-up:* NA | *N:* 24  *Gender:* M  *Age category:* Adult  *Diagnosis:* Psychopathy  *Offender type:* NS  *Controls:* M (23): Offenders, intermediate psychopathy, M (24): Offenders, low psychopathy | *Outcome measure:* Brain activity, eye movement  *Assessment method:* EEG, EOG  *Function:* Etiologic  *Stimulus/task:* Visual search task | Participants with high psychopathy scores showed an exaggerated N2pc response to set-congruent information and this provided novel electrophysiological evidence that psychopathy is associated with exaggerated endogenous attention effects during early stages of processing |
| Krylova et al. (2021) | To examine neural correlates of visual erotic processing in paedophilic sexual offenders using MEG and investigate whether P+CSO exhibits differential vMMNm amplitudes and/or latencies elicited by sexual images of adults and children relative to healthy controls | *Design*: Case-control study  *Country:* Germany  *Setting:* Inpatient clinic  *Follow-up:* NA | *N:* 17  *Gender:* M  *Age category:* Adult  *Diagnosis:* Paedophilia  *Offender type:* Sex offenders: Child victims  *Controls:* M (20): Healthy controls | *Outcome measure:* Brain activity, ERF  *Assessment method:* MEG  *Function:* Etiologic  *Stimulus/task:* Sexual images depicting adults and children | P+CSO exhibited significantly longer vMMNm latencies than HC. Moreover, P+CSO showed widespread increased amplitudes in response to child images starting from P3a and P3b components and lasting up to 400ms post-stimulus presentation localized in frontal and temporal brain regions |
| Kumari et al. (2006) | To investigate and compare brain activation during an n-back working memory task in groups of men with schizophrenia and a history of violence, schizophrenia without a history of violence, ASPD and a history of physical violence, and no history of violence or a mental disorder | *Design*: Case-control study  *Country:* UK  *Setting:* Inpatient clinic and outpatient clinic  *Follow-up:* NA | *N:* 25  *Gender:* M  *Age category:* Adult  *Diagnosis:* Mixed diagnoses  *Offender type:* Violent offenders  *Controls:* M (13): Healthy controls | *Outcome measure:* Brain activity  *Assessment method:* fMRI  *Function:* Etiologic  *Stimulus/task:* N-back working memory task | Reduced functional response in the frontal and inferior parietal regions leads to serious violence in schizophrenia perhaps via impaired executive functioning |
| Kumari et al. (2009) | To examine behavioral and brain abnormalities in violent men with schizophrenia or APD during anticipatory fear | *Design*: Case-control study  *Country:* UK  *Setting:* Inpatient clinic and outpatient clinic  *Follow-up:* NA | *N:* 39  *Gender:* M  *Age category:* Adult  *Diagnosis:* Mixed diagnoses  *Offender type:* Violent offenders  *Controls:* M (14): Healthy controls | *Outcome measure:* Brain activity  *Assessment method:* fMRI  *Function:* Etiologic  *Stimulus/task:* Safe (control) condition and a ‘threat of shock’ (experimental) condition | Aberrant activity in occipital and temporal regions when exposed to sustained visual threat cues is associated with a predisposition to violence in both schizophrenia and APD. This appears to arise from dissimilar behavioral mechanisms related to differences in the strength of aversive conditioning and behavioural response to sustained threat cues |
| Kumari et al. (2005) | To investigate prepulse inhibition of the startle response in antisocial personality disorder and schizophrenia in relation to a history of serious violence | *Design*: Case-control study  *Country:* UK  *Setting:* Inpatient clinic and outpatient clinic  *Follow-up:* NA | *N:* 18  *Gender:* M  *Age category:* Adult  *Diagnosis:* Antisocial personality disorder, schizophrenia  *Offender type:* Violent offenders  *Controls:* M (14): Outpatients, non-violent and schizophrenic, M (14): Healthy controls | *Outcome measure:* Fear-potentiated startle  *Assessment method:* EMG  *Function:* Etiologic  *Stimulus/task:* Noise (audio) stimuli | PPI deficits were most severe in the APD group, both schizophrenia groups also showed significant impairment relative to the healthy group but no significant difference in PPI was found between violent and non-violent schizophrenia patients. Higher ratings of violence were negatively associated with PPI suggesting that neural structures and functions underlying PPI are implicated in (inhibition of) violence |
| Lake et al. (2011) | To evaluate whether the differential sensitivity to threat stimuli associated with psychopathy and anxiety is better understood as opposite ends of a unitary continuum or as a reflection of fundamentally different processes | *Design*: Cross-sectional study  *Country:* USA  *Setting:* Correctional facility  *Follow-up:* NA | *N:* 87  *Gender:* M  *Age category:* Adult  *Diagnosis:* Psychopathy/psychopathic traits  *Offender type:* Violent and non-violent offenders  *Controls:* NA | *Outcome measure:* Fear potentiated startle  *Assessment method:* EMG  *Function:* Etiologic  *Stimulus/task:* Instructed fear-conditioning task | The abnormal sensitivity to threat cues associated with psychopathy and anxiety relates to different underlying processes, which has implications for understanding the relationship between low- and high-anxious psychopathy |
| Lalumière et al. (1998) | To test the prediction that sexual preferences for prepubertal individuals and non-consenting, violent sexual activities are associated with a greater number of older brothers but not sisters | *Design*: Cross-sectional study  *Country:* Canada  *Setting:* Inpatient clinic  *Follow-up:* NA | *N:* 78  *Gender:* M  *Age category:* Adult  *Diagnosis:* Personality disorder  *Offender type:* Sex offenders: Child and adult victims  *Controls:* NA | *Outcome measure:* Change in penis circumference  *Assessment method:* PPG  *Function:* Etiologic  *Stimulus/task:* Audio and visual (erotic/sexual) stimuli | Among a mixed group of male sexual offenders, the number of older brothers (but not older sisters) was positively correlated with the strength of deviant sexual preferences as measured by phallometric tests |
| Lalumière et al. (2003) | To examine whether rapists are sexually aroused by coercive, non-consensual sex | *Design*: Cross-sectional study  *Country:* Canada  *Setting:* Inpatient clinic  *Follow-up:* NA | *N:* 24  *Gender:* M  *Age category:* Adult  *Diagnosis:* Suspected paraphilia  *Offender type:* Sex offenders: Adult victims  *Controls:* M (11): Non-sex offenders, M (19): Healthy controls | *Outcome measure:* Change in penis circumference  *Assessment method:* PPG  *Function:* Diagnostic  *Stimulus/task:* Audiotaped (erotic/sexual) narratives | Comparison participants showed a preference for consenting scenarios, whereas rapists showed little discrimination between rape and consenting scenarios, and perhaps a slight preference for rape |
| Langevin et al. (1987) | To compare neuropsychological functioning, diagnoses, and substance abuse among killers, non-homicidal assaulters, and non-violent offender controls | *Design*: Case-control study  *Country:* Canada  *Setting:* NS  *Follow-up:* NA | *N:* 39  *Gender:* M  *Age category:* Adult  *Diagnosis:* Personality disorder  *Offender type:* Violent offenders  *Controls:* M (16): Offenders, non-violent | *Outcome measure:* Brain activity  *Assessment method:* EEG  *Function:* Etiologic  *Stimulus/task:* Baseline, hyperventilation, and photic stimulation | Neuropsychological variables are significant in one-fifth to one-quarter of violent offenders. Killers are more like non-homicidal violent offenders than non-violent offenders |
| Langevin and Curnoe (2010) | To examine the relationship of psychopathy, ADHD and brain dysfunction in a sample of adult male sexual, violent, and non-violent offenders | *Design*: Cross-sectional  *Country:* Canada  *Setting:* Inpatient clinic and outpatient clinic  *Follow-up:* NA | *N:* 1520  *Gender:* M  *Age category:* Adult  *Diagnosis:* Psychopathy/psychopathic traits, paraphilia  *Offender type:* Sex offenders: Victim not specified  *Controls:* M (133): Violent non-sex offenders, M (42): Non-violent non-sex offenders | *Outcome measure:* Brain activity  *Assessment method:* EEG  *Function:* Etiologic  *Stimulus/task:* NA | Psychopathy is significantly associated with ADHD and brain dysfunction, which association should be considered in programs for treatment and prevention of criminal behavior |
| Langevin and Curnoe (2011) | To examine the best predictor of lifetime recidivism among PCL-R scores, ADHD diagnosis, and brain dysfunction measures in a sample of adult male sexual, violent, and non-violent offenders | *Design*: Cohort, retrospective study  *Country:* Canada  *Setting:* Inpatient clinic and outpatient clinic  *Follow-up:* Not explicitly stated, presumably several years | *N:* 1520  *Gender:* M  *Age category:* Adult  *Diagnosis:* Psychopathy/psychopathic traits, paraphilia  *Offender type:* Sex offenders: Victim not specified  *Controls:* M (133): Violent non-sex offenders, M (42): Non-violent non-sex offenders | *Outcome measure:* Brain activity  *Assessment method:* EEG  *Function:* Prognostic  *Stimulus/task:* NA | General recidivism was primarily associated with past criminal history and secondarily with learning disorders and ADHD |
| Larson et al. (2013) | To address the paucity of neuroimaging data available to specify the neural mechanisms mediating the attention-related modulation of emotion processing in psychopathy | *Design*: Case-control study  *Country:* USA  *Setting:* Correctional facility  *Follow-up:* NA | *N:* 24  *Gender:* M  *Age category:* Adult  *Diagnosis:* Psychopathy  *Offender type:* NS  *Controls:* M (25): Offenders, non-psychopathic | *Outcome measure:* Brain activity  *Assessment method:* fMRI  *Function:* Etiologic  *Stimulus/task:* Instructed fear task and electrical stimulation | Psychopaths’ amygdala-mediated fear deficit appears and disappears as a function of attention-related priorities. Psychopaths’ failure to recruit the amygdala in response to salient emotion cues while pursuing immediate goals may be a manifestation of a more general LPFC-instantiated attention bottleneck that severely limits the processing of potentially important peripheral information |
| Laws et al. (2000) | To examine the extent to which the use of multiple measures of paedophilic interest improved the diagnostic accuracy of any single measure | *Design*: Cross-sectional study  *Country:* USA  *Setting:* Outpatient clinic  *Follow-up:* NA | *N:* 72  *Gender:* M  *Age category:* Adult  *Diagnosis:* Not explicitly stated  *Offender type:* Sex offenders: Child victims  *Controls:* NA | *Outcome measure:* Change in penis circumference  *Assessment method:* PPG  *Function:* Diagnostic  *Stimulus/task:* Erotic slides or audio material | All three measures of paedophilic interest significantly differentiated boy-object and girl-object child molesters. The card-sort measure showed the greatest classification accuracy and was the only measure to significantly improve accuracy, once the other two modalities were considered |
| Levenston et al. (2000) | To compare the reactions of psychopaths and non-psychopaths to distinct subcategories of pleasurable and aversive pictures and examine the dynamic interplay of attention and emotion in the processing of these affective stimuli by measuring reflex reactions to noise probes at sequential times during the picture viewing interval | *Design*: Case-control study  *Country:* USA  *Setting:* Correctional facility  *Follow-up:* NA | *N:* 18  *Gender:* M  *Age category:* Adult  *Diagnosis:* Psychopathy  *Offender type:* NS  *Controls:* M (18): Offenders, non-psychopathic | *Outcome measure:* Skin conductance, blink response, heart rate, facial electromyographic activity  *Assessment method:* SCD, EMG, Coulbourn S75-01 High Gain Bioamplifier (HR)  *Function:* Etiologic  *Stimulus/task:* Pleasant and unpleasant slide pictures and neutral pictures | Non-psychopaths showed moderate and strong reflex potentiation for victim and threat scenes, respectively. For psychopaths, startle was inhibited during victim scenes and only weakly potentiated during threat and they showed more reliable blink inhibition across pleasant contents than non-psychopaths and greater heart rate orienting to affective pictures. The results indicate a heightened aversion threshold in psychopaths |
| Levy and Kennard (1953) | To discover whether any relationship exists between type of EEG record and the personality structure in a group of inmates | *Design*: Case-control study  *Country:* Canada  *Setting:* NS  *Follow-up:* NA | *N:* 50  *Gender:* M  *Age category:* Adult  *Diagnosis:* Psychopathy  *Offender type:* Violent offenders  *Controls:* M (50): Non-violent offenders | *Outcome measure:* Brain activity  *Assessment method:* EEG  *Function:* Etiologic  *Stimulus/task:* Questions were asked during the EEG | In the present study EEG abnormalities were found to be equally distributed between crimes of violence and non-violence |
| Lievaart et al. (2018) | To investigate whether individual differences in trait anger in forensic psychiatric patients are associated with individual differences in anger-primed inhibitory control using behavioral and electrophysiological measures of response inhibition | *Design*: Cross-sectional study  *Country:* The Netherlands  *Setting:* Inpatient clinic  *Follow-up:* NA | *N:* 38  *Gender:* M (25) + F (13)  *Age category:* Adult  *Diagnosis:* Mixed diagnoses  *Offender type:* Violent offenders  *Controls:* NA | *Outcome measure:* Brain activity  *Assessment method:* EEG  *Function:* Etiologic  *Stimulus/task:* Aggression‑related Go-NoGo task | In a forensic population trait anger is inversely related to impulse control, particularly in hostile contexts and higher scores on trait anger are associated with deficits in automatic error-processing which may contribute the continuation of impulsive angry behaviors despite their negative consequences |
| Lijffijt et al. (2012) | To compare electrophysiological measures of auditory sensory gating assessed by the paired-click paradigm in males with ASPD to healthy controls | *Design*: Case-control study  *Country:* USA  *Setting:* Correctional facility  *Follow-up:* NA | *N:* 37  *Gender:* M  *Age category:* Adult  *Diagnosis:* Antisocial personality disorder  *Offender type:* Violent offenders  *Controls:* M (28): NS | *Outcome measure:* Brain activity  *Assessment method:* EEG  *Function:* Etiologic  *Stimulus/task:* Paired-click paradigm | Controls and ASPD did not differ in P50, N100, or P200 amplitude or ASG. Past alcohol or drug use disorders had no effect. In controls, impulsivity was related to improved P50 and P200 gating. In ASPD, P50 or N100 gating was impaired with more symptoms or increased impulsivity, respectively, suggesting impaired early filtering of irrelevant information. In controls the relationship between P50 and P200 gating and impulsivity was reversed, suggesting better gating with higher impulsivity scores |
| Lijffijt et al. (2017) | To investigate whether enhanced stimulus orienting operationalized as N1 and P2 auditory evoked potentials to increasing loudness can be associated with trait impulsivity, impulsive action, or impulsive choice | *Design*: Case-control study  *Country:* USA  *Setting:* Correctional facility  *Follow-up:* NA | *N:* 36  *Gender:* M  *Age category:* Adult  *Diagnosis:* Antisocial personality disorder  *Offender type:* Mixed offender types  *Controls:* M (16): Healthy controls | *Outcome measure:* Brain activity  *Assessment method:* EEG  *Function:* Etiologic  *Stimulus/task:* Tones and intensity-sensitivity task, paired-click paradigm, passive oddball task and Flanker task | Study results suggest an association between enhanced early stimulus orienting, impulsive action, and impaired signal-noise discriminability |
| Lindberg et al. (2003) | To characterize sleep of habitually violent offenders with antisocial personality disorder | *Design*: Case-control study  *Country:* Finland  *Setting:* Inpatient clinic  *Follow-up:* NA | *N:* 19  *Gender:* M  *Age category:* Adult  *Diagnosis:* Antisocial personality disorder  *Offender type:* Violent offenders  *Controls:* M (11): Healthy controls | *Outcome measure:* Brain activity, movement  *Assessment method:* EEG (polysomnography), actigraph (wrist-worn)  *Function:* Etiologic  *Stimulus/task:* NA (resting-state EEG) | Results suggest increased amount of slow-wave sleep and elevated delta and theta power in antisocial violent offenders |
| Lindberg et al. (2004) | To examine a possible relationship between childhood ADHD and sleep architecture in habitually violent men with antisocial personality disorder and alcoholism | *Design*: Case-control study  *Country:* Finland  *Setting:* Inpatient clinic  *Follow-up:* NA | *N:* 14  *Gender:* M  *Age category:* Adult  *Diagnosis:* Antisocial personality disorder, substance use disorder  *Offender type:* Violent offenders  *Controls:* M (10): Healthy controls | *Outcome measure:* Brain activity  *Assessment method:* EEG (polysomnography), actigraph (wrist-worn)  *Function:* Etiologic  *Stimulus/task:* NA (resting-state EEG) | Childhood ADHD is associated with abnormal sleep architecture in habitually violent men with antisocial personality disorder; childhood ADHD and ASP seem to (partly) share the same central nervous system deficit |
| Lindberg et al. (2006) | To characterize sleep in severely violent women with antisocial personality disorder | *Design*: Case-control study  *Country:* Finland  *Setting:* Inpatient clinic  *Follow-up:* NA | *N:* 3  *Gender:* F  *Age category:* Adult  *Diagnosis:* Antisocial personality disorder  *Offender type:* Violent offenders  *Controls:* F (10): Healthy controls | *Outcome measure:* Brain activity  *Assessment method:* EEG (polysomnography)  *Function:* Etiologic  *Stimulus/task:* NA (resting-state EEG) | Increased amount of slow-wave sleep in cases. As in males, severe female aggression seems to be associated with profound changes in sleep architecture |
| Lindberg et al. (2005) | To analyze quantitative EEG of habitually violent homicidal offenders with antisocial personality disorder to assess daytime vigilance | *Design*: Case-control study  *Country:* Finland  *Setting:* Inpatient clinic  *Follow-up:* NA | *N:* 16  *Gender:* M  *Age category:* Adult  *Diagnosis:* Antisocial personality disorder  *Offender type:* Violent offenders  *Controls:* M (15): Healthy controls | *Outcome measure:* Brain activity  *Assessment method:* EEG  *Function:* Etiologic  *Stimulus/task:* NA (resting-state EEG) | Results suggest an overall reduction in alpha power and bilateral increase in occipital delta and theta power in cases, indicating decreased daytime vigilance in cases and thus brain dysfunction |
| Liu et al. (2014) | To investigate the underlying neural mechanisms of APD using resting-state functional magnetic resonance imaging | *Design*: Case-control study  *Country:* China  *Setting:* Correctional facility  *Follow-up:* NA | *N:* 32  *Gender:* M  *Age category:* Adult  *Diagnosis:* Personality disorder  *Offender type:* Violent offenders  *Controls:* M (35): Healthy controls | *Outcome measure:* Brain activity  *Assessment method:* fMRI  *Function:* Etiologic  *Stimulus/task:* NA (resting-state fMRI) | APD patients had a significant reduction in the ALFF in the right orbitofrontal cortex, the left temporal pole, the right inferior temporal gyrus, and the left cerebellum posterior lobe compared to normal controls, The right orbitofrontal cortex had a negative correlation between ALFF values and MMPI psychopathic deviate scores. Alterations in ALFF in these specific brain regions suggest that APD patients may be associated with abnormal activities in the frontotemporal network |
| Lobbesteal and Arntz (2010) | To compare the emotional reactivity to abuse-related stress of patients with borderline personality disorder and antisocial personality disorder on a direct and indirect level | *Design*: Case-control study  *Country:* The Netherlands  *Setting:* Correctional facility, inpatient clinic and outpatient clinic  *Follow-up:* NA | *N:* 45 (BPD), 21 (ASPD), 46 (Cluster C)  *Gender:* M (45) + F (67)  *Age category:* Adult  *Diagnosis:* Personality disorder  *Offender type:* NS  *Controls:* M (16) - F (19): Healthy controls | *Outcome measure:* Skin conductance, heart rate, systolic blood pressure, diastolic blood pressure, facial electromyographic activity  *Assessment method:* Omron M5-I (SBP and DBP), Vitaport III system (HR, SCR [skin conductance response], SCL [skin conductance level], EMG)  *Function:* Etiologic  *Stimulus/task:* Abuse scene of 20 min derived from the movie ‘No child of mine’ | BPD and ASPD patients are alike in their implicit cognitive abuse-related stress reactivity, but can be differentiated in their self-reported and physiological response patterns |
| Lobbesteal et al. (2009) | To examine emotional, cognitive, and physiological correlates of anger and compared these between ASPD patients with varying degrees of psychopathy and control groups | *Design*: Case-control study  *Country:* The Netherlands  *Setting:* Correctional facility, inpatient clinic and outpatient clinic  *Follow-up:* NA | *N:* 45 (BPD), 21 (ASPD), 46 (Cluster C)  *Gender:* M (45) + F (67)  *Age category:* Adult  *Diagnosis:* Personality disorder  *Offender type:* NS  *Controls:* M (16) - F (19): Healthy controls | *Outcome measure:* Skin conductance, heart rate, systolic blood pressure, diastolic blood pressure, facial electromyographic activity  *Assessment method:* Omron M5-I (SBP and DBP), Vitaport III system (HR, SCR [skin conductance response], SCL [skin conductance level], EMG)  *Function:* Etiologic  *Stimulus/task:* A stress-induction interview | ASPD patients did not display deviant self-reported anger but physiological hyporesponsivity and cognitive hyper-responsivity. This ASPD anger response might reflect a controlled predatory-like fight preparation |
| Loewinger-Cloyd (2007) | To evaluate if there is a positive association between the results of two measures utilized in the evaluation of males who have sexually offended: the Monarch 21 PPG and the Affinity measure of viewing time | *Design*: Cross-sectional study  *Country:* USA  *Setting:* Outpatient clinic  *Follow-up:* NA | *N:* 96  *Gender:* M  *Age category:* Adult  *Diagnosis:* Not explicitly stated  *Offender type:* Sex offenders: Victim not specified  *Controls:* NA | *Outcome measure:* Change in penis circumference  *Assessment method:* PPG  *Function:* Diagnostic  *Stimulus/task:* Audio and visual sexual stimuli (different gender and age stimuli) | Significant but modest associations were found between the Monarch 21 PPG and the Affinity (including sexual deviance ratios and differentials) |
| Looman (2000) | To investigate sexual arousal in rapists using two stimulus sets (audiotapes) | *Design*: Case-control study  *Country:* Canada  *Setting:* Inpatient clinic  *Follow-up:* NA | *N:* 180  *Gender:* M  *Age category:* Adult  *Diagnosis:* Not explicitly stated  *Offender type:* Sex offenders: Child and adult victims  *Controls:* M (71): Child molesters, M (37): Mixed sex offenders | *Outcome measure:* Change in penis circumference  *Assessment method:* PPG  *Function:* Diagnostic  *Stimulus/task:* (Sexual/erotic) audiotapes | Neither stimulus set distinguished the groups of rapists, child molesters and mixed offenders in terms of the rape indices; also, only 25% of rapists were classified as deviant |
| Loomans et al. (2015) | To explore an eye blink startle paradigm as a means of distinguishing between men with both antisocial personality disorder and psychopathy, and men with ASPD alone | *Design*: Case-control study  *Country:* The Netherlands  *Setting:* Inpatient clinic  *Follow-up:* NA | *N:* 53  *Gender:* M  *Age category:* Adult  *Diagnosis:* Psychopathy, antisocial personality disorder  *Offender type:* NS  *Controls:* M (50): Staff members, M (33): Healthy controls | *Outcome measure:* Startle response  *Assessment method:* EMG  *Function:* Diagnostic and etiologic  *Stimulus/task:* Visual stimuli (pleasant, unpleasant and neutral pictures) and acoustic probes | Eye blink startle modulation deficiencies among men with psychopathy were replicated; the psychopathy and ASPD groups could be distinguished by startle stimulus onset asynchrony, but this pattern was also seen in one healthy group – the forensic hospital employees |
| Lu et al. (2015) | To examine whether functional connectivity in RSNs are different between adolescents with CD and the typically-developing group and that the alterations in the brain’s neuronal circuit have a powerful influence on the brain organization of adolescents with CD | *Design*: Case-control study  *Country:* China  *Setting:* Correctional facility  *Follow-up:* NA | *N:* 18  *Gender:* M  *Age category:* Juvenile  *Diagnosis:* Conduct disorder  *Offender type:* NS  *Controls:* M (18): Healthy controls | *Outcome measure:* Brain activity  *Assessment method:* fMRI  *Function:* Etiologic  *Stimulus/task:* NA (resting-state fMRI) | Compared with the TD group, the CD group manifested decreased functional connectivity in four representative RSNs: the anterior default mode network, which is considered to be correlated with impaired social cognition, the somatosensory network, the lateral visual network, and the medial visual network, which are expected to be relevant to the perceptual systems responsible for perceptual dysfunction in male adolescents with CD |
| Lu et al. (2020) | A voxel-mirrored homotopic connectivity method based on rsfMRI was employed for the first time to examine the abnormalities of interhemispheric functional connectivity in patients with CD | *Design*: Case-control study  *Country:* China  *Setting:* Correctional facility  *Follow-up:* NA | *N:* 18  *Gender:* M  *Age category:* Juvenile  *Diagnosis:* Conduct disorder  *Offender type:* NS  *Controls:* M (18): Healthy controls | *Outcome measure:* Brain activity  *Assessment method:* fMRI  *Function:* Etiologic  *Stimulus/task:* NA (resting-state fMRI) | In CD patients, reduced homotopic connectivity was observed relative to TDs in the middle occipital gyrus, pre-and postcentral gyrus, Rolandic operculum, and paracentral lobe (components of visual and motor networks). The VMHC of the MOG and PCL was found to be negatively correlated with clinical scores in the CD group. The regions with altered VMHC exhibited a relatively good and robust ability to discriminate CD patients from TDs |
| Lu et al. (2021) | To examine whether the dynamic characteristics of lBA might be disrupted in CD patients during resting-state and whether these abnormalities may be associated with clinical features | *Design*: Case-control study  *Country:* China  *Setting:* Correctional facility  *Follow-up:* NA | *N:* 18  *Gender:* M  *Age category:* Juvenile  *Diagnosis:* Conduct disorder  *Offender type:* NS  *Controls:* M (18): Healthy controls | *Outcome measure:* Brain activity  *Assessment method:* fMRI  *Function:* Etiologic  *Stimulus/task:* NA (resting-state fMRI) | The findings extended previous work by providing a novel perspective on the neural mechanisms underlying adolescent patients with CD and demonstrated that the altered dynamic local brain activity may be a potential biomarker for CD diagnosis |
| Luckhaus et al. (2013) | To investigate treatment effects of Training of Affect Recognition (TAR) in schizophrenia patients with violent offences | *Design*: Cross-over clinical trial (RCT; waiting group condition) and pre-post treatment (within subject) design, longitudinal study  *Country:* Germany  *Setting:* Inpatient clinic  *Follow-up:* 2 months | *N:* 10  *Gender:* M  *Age category:* Adult  *Diagnosis:* Schizophrenia  *Offender type:* Violent offenders  *Controls:* M (9): Patients, waiting group condition | *Outcome measure:* ERP  *Assessment method:* EEG; sLORETA (standardized low resolution electromagnetic tomography)  *Function:* Monitoring  *Stimulus/task:* Pictures of facial affect (PFA)-test | Violent offenders with schizophrenia were amenable to TAR. Post- versus pretreatment changes of neural activity (assessed with LORETA; no changes in ERPs) may mirror a gain of efficiency in structural face decoding and a shift towards a more reflective mode of emotional face decoding, relying on increased frontal brain activity |
| Lykins et al. (2010) | To investigate the relation between magnitude of penile response to two different phallometric tests for paedophilia and the agreement in diagnoses | *Design*: Cross-sectional study  *Country:* Canada  *Setting:* Outpatient clinic  *Follow-up:* NA | *N:* 79  *Gender:* M  *Age category:* Adult  *Diagnosis:* Paedophilia  *Offender type:* Sex offenders: Child and adult victims  *Controls:* NA | *Outcome measure:* Change in penis circumference  *Assessment method:* PPG  *Function:* Diagnostic  *Stimulus/task:* Visual (sexual/erotic) stimuli | Consistency of diagnosis (obtained from different phallometric tests) increases as penile responding increases |
| Lykins et al. (2010) | To investigate sexual arousal to female children in gynephilic men | *Design*: Cross-sectional study  *Country:* Canada  *Setting:* Outpatient clinic  *Follow-up:* NA | *N:* 214  *Gender:* M  *Age category:* Adult  *Diagnosis:* Not explicitly stated  *Offender type:* Sex offenders: Adult victims  *Controls:* NA | *Outcome measure:* Change in penis circumference  *Assessment method:* PPG  *Function:* Monitoring  *Stimulus/task:* Audiotaped (sexual/erotic) narratives | In gynephilic men, sexual arousal to both pubescent and prepubescent girls was significantly greater than to neutral stimuli. This could explain why gynephylic men with unusually weak inhibitory control, in circumstances where weakly erotic objects (pre-pubescent girls) are more available than strongly erotic objects (adult women) can become child molesters |
| MacDougall (2016) | To investigate the association between adolescent psychopathy and stimulus-evoked autonomic response assessed with heart rate and skin conductance | *Design*: Cross-sectional study  *Country:* USA  *Setting:* Correctional facility  *Follow-up:* NA | *N:* 56  *Gender:* M  *Age category:* Juvenile  *Diagnosis:* Conduct disorder, juvenile psychopathic traits  *Offender type:* Mixed offender types  *Controls:* NA | *Outcome measure:* Skin conductance, heart rate  *Assessment method:* BioLog recorder (with heart and skin conductance electrodes)  *Function:* Monitoring  *Stimulus/task:* White noise countdown task | No significant association between psychopathy and heart rate was found, but a positive association between grandiose-manipulative traits and skin conductance activity and a negative association between callous-unemotional traits and SC activity; thus autonomic processes may contribute to distinct psychopathic traits in different ways, implicating slightly differential brain functioning |
| Mackaronis (2014) | To compare unobtrusive (a viewing time measure), physiologically-based (penile plethysmography), and actuarially based methods to assess sexual interest in adolescent child sex offenders | *Design*: Cross-sectional study  *Country:* USA  *Setting:* Inpatient clinic  *Follow-up:* NA | *N:* 16  *Gender:* M  *Age category:* Adult and juvenile  *Diagnosis:* Not explicitly stated  *Offender type:* Sex offenders: Child victims  *Controls:* NA | *Outcome measure:* Change in penis circumference  *Assessment method:* PPG  *Function:* Monitoring  *Stimulus/task:* Audio and visual neutral and (deviant) sexual stimuli (narratives and pictures) | Viewing time and PPG assessments showed some overlap; actuarially-based assessment may have limited utility with adolescents |
| Madsen et al. (2004) | To investigate whether periodic polygraph testing in sex offenders acted as a deterrent for engaging in risk behaviour | *Design*: Quasi-experimental (partially RCT), longitudinal study  *Country:* UK  *Setting:* Inpatient clinic and outpatient clinic  *Follow-up:* 6 months | *N:* 27  *Gender:* M  *Age category:* Adult  *Diagnosis:* Not explicitly stated  *Offender type:* Sex offenders: Child and adult victims  *Controls:* M (22): Sex offenders, polygraph aware group | *Outcome measure:* Skin resistance, cardiovascular activity, respiration  *Assessment method:* Polygraph  *Function:* Intervention  *Stimulus/task:* Interview during polygraphic assessment | Polygraph testing had an impact on the level of seriousness of the risk behaviours engaged in by sex offenders, but this only occured after experience of the test itself; high refusal and attrition rates suggested that some offenders sought to avoid the polygraph |
| Maes et al. (2001) | To examine cortisol, prolactine, and body temperature at baseline and meta-chlorophenylpiperazine induced in men with paedophilia | *Design*: Case-control, longitudinal study  *Country:* Belgium  *Setting:* Outpatient clinic  *Follow-up:* 3 hours | *N:* 8  *Gender:* M  *Age category:* Adult  *Diagnosis:* Paedophilia  *Offender type:* Sex offenders: Child victims  *Controls:* M (11): Healthy controls | *Outcome measure:* Body temperature  *Assessment method:* Tympanic thermometer  *Function:* Etiologic  *Stimulus/task:* Metachlorophenylpiperazine (mCPP) administration | Paedophiles showed lower cortisol and prolactin levels and higher body temperature at baseline and higher cortisol and lower hyperthermic responses after mCPP administration than normal volunteers. This suggests several serotonergic disturbances in paedophiles |
| Maletzky (1993) | To investigate factors associated with success and failure in behavioral and cognitive treatment of child sexual offenders | *Design*: Cohort study  *Country:* USA  *Setting:* Inpatient clinic and outpatient clinic  *Follow-up:* 19 years | *N:* 4381  *Gender:* M  *Age category:* Adult  *Diagnosis:* Paedophilia  *Offender type:* Sex offenders: Child victims  *Controls:* NA | *Outcome measure:* Change in penis circumference  *Assessment method:* PPG  *Function:* Monitoring  *Stimulus/task:* (Deviant) sexual stimuli: Slides, videotaped scenes and fantasies read to the participant | Treatment success was often associated with a minimum number of victims, familiarity and cohabitation with victims, an absence of force or threats employed in the crimes, admission of some responsibility for the offending incidents, and a stable history of employment and relationships with others. Plethysmograph findings confirmed that low pre-treatment deviant arousal was correlated with improvement |
| Mannfolk et al. (2023) | To investigate neural and behavioral responses to sexually implicit stimuli during a pictorial-modified Stroop Task in individuals with paedophilic disorder | *Design*: Case-control study  *Country:* Sweden  *Setting:* Outpatient clinic  *Follow-up:* NA | *N:* 51  *Gender:* M  *Age category:* Adult  *Diagnosis:* Paedophilia  *Offender type:* Sex offenders: Child victims or not specified  *Controls:* M (55): Healthy controls | *Outcome measure:* Brain activity  *Assessment method:* fMRI  *Function:* Etiologic  *Stimulus/task:* Pictorial-modified Stroop task | Presentation of child images was associated with response interference in paedophilic disorder and increased engagement of brain regions involved in the processing of sexual stimuli, visual perception, self-referential thought, and executive function. Results suggest that processing of child images is associated with functional and behavioral alterations in PD |
| Marsch (2013) | To investigate the differences in neural response of incarcerated men with and without psychopathy when viewing images of other people being hurt or expressing pain | *Design*: Case-control study  *Country:* USA  *Setting:* Correctional facility  *Follow-up:* NA | *N:* 27  *Gender:* M  *Age category:* Adult  *Diagnosis:* Psychopathy  *Offender type:* NS  *Controls:* M (53): Offenders, intermediate or low psychopathy | *Outcome measure:* Brain activity  *Assessment method:* fMRI  *Function:* Etiologic  *Stimulus/task:* Pain interactions tasks using visual scenarios | When viewing empathy-eliciting scenarios, incarcerated men with high psychopathy had lower activation of the orbitofrontal cortex and ventromedial prefrontal cortex and higher activation of the anterior insular cortex compared with men with low psychopathy |
| Marschall-Levésque et al. (2013) | To investigate the differences in neural response of incarcerated men with and without psychopathy when viewing images of other people being hurt or expressing pain | *Design*: Case-control study  *Country:* Canada  *Setting:* Inpatient clinic  *Follow-up:* NA | *N:* 15  *Gender:* M  *Age category:* Adult  *Diagnosis:* Not explicitly stated  *Offender type:* Sex offenders: Child victims  *Controls:* M (15): Healthy controls | *Outcome measure:* Change in penis circumference  *Assessment method:* PPG  *Function:* Diagnostic  *Stimulus/task:* Audio and visual sexual stimuli using computer generated characters | Computer-generated characters allow accurate discrimination of sex offenders with child victims and can be added to already validated (audio) stimuli to increase the number of valid profiles |
| Martin and Johnson (2006) | To examine the effects of EEG neurofeedback training on sustained attention, response inhibition, executive functions, intellectual ability, and memory in incarcerated juveniles with ADHD or conduct disorder | *Design*: Quasi-experimental, within subjects and pre-post design, longitudinal study  *Country:* USA  *Setting:* Correctional facility  *Follow-up:* NA | *N:* 7  *Gender:* M  *Age category:* Juvenile  *Diagnosis:* Conduct disorder, ADHD  *Offender type:* NS  *Controls:* NA | *Outcome measure:* Brain activity  *Assessment method:* EEG  *Function:* Intervention  *Stimulus/task:* Visual (animations) and auditory (sounds) stimuli during EEG assessment for neurofeedback training | EEG biofeedback may be a useful adjunct in the treatment of juvenile offenders |
| Massau et al. (2017) | To assess differences in neural mechanisms of moral judgment associated with paedophilia and/or sexual offending against children using fMRI | *Design*: Case-control study  *Country:* Germany  *Setting:* Inpatient clinic and outpatient clinic  *Follow-up:* NA | *N:* 16  *Gender:* M  *Age category:* Adult  *Diagnosis:* Paedophilia  *Offender type:* Sex offenders: Child victims  *Controls:* M (19): Healthy controls, M (15): Non-offender paedophiles | *Outcome measure:* Brain activity  *Assessment method:* fMRI  *Function:* Etiologic  *Stimulus/task:* Moral judgment task | Paedophiles, regardless of whether they have committed a sexual offense against a child or not, judge and process sexual aggression differently compared to non-paedophilic, healthy male controls |
| Maurer et al. (2016) | To examine response-locked ERP components in a sample of incarcerated juvenile male offenders with elevated psychopathic traits who performed a response inhibition Go-NoGo task | *Design*: Cross-sectional study  *Country:* USA  *Setting:* Correctional facility  *Follow-up:* NA | *N:* 100  *Gender:* M  *Age category:* Juvenile  *Diagnosis:* Psychopathic traits  *Offender type:* NS  *Controls:* NA | *Outcome measure:* Brain activity  *Assessment method:* EEG  *Function:* Etiologic  *Stimulus/task:* Go-NoGo task | Study results suggest a negative relationship between adolescent psychopathy scores and Pe mean amplitude |
| Maurer et al. (2016) | To examine response-locked ERP components in a sample of incarcerated adult female offenders with elevated psychopathic traits who performed a response inhibition Go-NoGo task | *Design*: Cross-sectional study  *Country:* USA  *Setting:* Correctional facility  *Follow-up:* NA | *N:* 121  *Gender:* F  *Age category:* Adult  *Diagnosis:* Psychopathy  *Offender type:* NS  *Controls:* NA | *Outcome measure:* Brain activity  *Assessment method:* EEG  *Function:* Etiologic  *Stimulus/task:* Go-NoGo task | Female psychopaths exhibited specific deficiencies in the neural correlates of post-error processing (as indexed by reduced Pe amplitude) but not in error monitoring (as indexed by intact ERN/Ne amplitude) |
| Maurer et al. (2018) | To investigate whether scores on four different self-report measures of adolescent psychopathic traits were associated with reduced Pe amplitude in a sample of incarcerated adolescent offenders | *Design*: Cross-sectional study  *Country:* USA  *Setting:* Correctional facility  *Follow-up:* NA | *N:* 100  *Gender:* M  *Age category:* Juvenile  *Diagnosis:* Psychopathic traits  *Offender type:* NS  *Controls:* NA | *Outcome measure:* Brain activity  *Assessment method:* EEG  *Function:* Etiologic  *Stimulus/task:* Go-NoGo task | Adolescent self-report psychopathy scores were not associated with reduced Pe amplitude in multiple regression analyses |
| Maurer et al. (2019) | To investigate error-related processing using a response inhibition Go-NoGo fMRI experimental paradigm in a sample of incarcerated male adolescent offenders | *Design*: Cross-sectional study  *Country:* USA  *Setting:* Correctional facility  *Follow-up:* NA | *N:* 182  *Gender:* M  *Age category:* Juvenile  *Diagnosis:* Psychopathic traits  *Offender type:* NS  *Controls:* NA | *Outcome measure:* Brain activity  *Assessment method:* fMRI  *Function:* Etiologic  *Stimulus/task:* Go-NoGo task | Adolescent psychopathic traits negatively related to hemodynamic activity within the basal ganglia during error-related processing |
| Mayer et al. (2019) | To investigate behavioral and electrophysiological responses to fairness norm violations (using gamification) in antisocial violent offenders | *Design*: Case-control study  *Country:* Germany  *Setting:* Correctional facility  *Follow-up:* NA | *N:* 25  *Gender:* M  *Age category:* Adult  *Diagnosis:* Antisocial personality disorder  *Offender type:* Violent offenders  *Controls:* M (24): Healthy controls | *Outcome measure:* Brain activity  *Assessment method:* EEG  *Function:* Etiologic  *Stimulus/task:* Playing the Dictator Game and the Ultimatum Game | Results suggest intact fairness norm representations but altered reactions to their violation in antisocial personality disorder |
| McDonald et al. (2021) | To examine associations between distinct facets of externalizing symptoms and relevant behavioral phenotypes as well as electrophysiological and behavioral indices of inhibitory control and error processing | *Design*: Cross-sectional study  *Country:* USA  *Setting:* Correctional facility, inpatient clinic, outpatient clinic and other  *Follow-up:* NA | *N:* 89  *Gender:* M (57) + F (32)  *Age category:* Adult  *Diagnosis:* Psychopathy, antisocial personality disorder, substance use disorder  *Offender type:* NS  *Controls:* M (24): NA | *Outcome measure:* Brain activity  *Assessment method:* EEG  *Function:* Etiologic  *Stimulus/task:* Variant of Flanker and Go-NoGo tasks | Externalizing was marked by deficits in error processing, but not inhibitory control |
| McGrath et al. (2007) | To examine the effect of polygraphy (intervention) on treatment outcomes (reoffending) in sex offenders | *Design*: Experimental, non-RCT (matched control group), longitudinal study  *Country:* USA  *Setting:* Correctional facility and outpatient clinic  *Follow-up:* 5 years | *N:* 104  *Gender:* M  *Age category:* Adult  *Diagnosis:* Not explicitly stated  *Offender type:* Sex offenders: Child and adult victims  *Controls:* M (104): No polygraph exam | *Outcome measure:* Galvanic skin response, heart rate, blood pressure, breathing patterns  *Assessment method:* Polygraph  *Function:* Intervention  *Stimulus/task:* Interview during polygraphic assessment | After 5 years of follow-up, people in the polygraphy group appeared to have been charged less often for non-sexual violent offenses, otherwise there were no differences |
| McKerracher and Dacre (1966) | To examine conditioned galvanic skin responses in arsonist and non-arsonist forensic inpatients | *Design*: Case-control study  *Country:* UK  *Setting:* Inpatient clinic  *Follow-up:* NA | *N:* 20  *Gender:* M  *Age category:* Adult  *Diagnosis:* Not explicitly stated  *Offender type:* Arsonists  *Controls:* M (88): Inpatients who committed other offences than arson | *Outcome measure:* Galvanic skin response  *Assessment method:* Polygraph  *Function:* Etiologic  *Stimulus/task:* Audio probes (unconditioned stimulus) and a puff of air ejected upon the eye (unconditional stimulus) | There was a tendency for arsonists to display greater galvanic skin response reactivity, and to give more conditioned responses than the non-arsonist offenders |
| McPhail et al. (2018) | To examine the latent structure of paedophilic interest using data from phallometric tests for paedophilic interest across four samples of offenders | *Design*: Cross-sectional study  *Country:* Canada  *Setting:* Correctional facility, inpatient clinic, and outpatient clinic  *Follow-up:* NA | *N:* 2229  *Gender:* M  *Age category:* Adult  *Diagnosis:* Not explicitly stated  *Offender type:* Sex offenders: Child and adult victims  *Controls:* NA | *Outcome measure:* Change in penis circumference  *Assessment method:* PPG  *Function:* Diagnostic  *Stimulus/task:* Audio and/or visual sexual stimuli (different gender and age stimuli) | Class analyses revealed a trichotomous latent structure, indicating the presence of a complement taxon and two paedophilic taxa (non-exclusively paedophilic and exclusively paedophilic) |
| Meffert et al. (2013) | To test the hypothesis that the psychopathic group would show less vicarious activity than controls for at least one stimulus condition | *Design*: Case-control study  *Country:* The Netherlands  *Setting:* Inpatient clinic  *Follow-up:* NA | *N:* 20  *Gender:* M  *Age category:* Adult  *Diagnosis:* Psychopathy  *Offender type:* NS  *Controls:* M (26): Healthy controls | *Outcome measure:* Brain activity  *Assessment method:* fMRI  *Function:* Etiologic  *Stimulus/task:* Viewing video clips of emotional hand interactions and while experiencing similar interactions | Brain regions involved in experiencing interactions were not spontaneously activated as strongly in the patient group while viewing the video clips. However, this group difference was markedly reduced when we specifically instructed participants to feel with the actors in the videos. Psychopathy is not a simple incapacity for vicarious activations but rather reduced spontaneous vicarious activations co-existing with relatively normal deliberate counterparts |
| Meier et al. (2012) | To examine neurophysiological correlates of delinquent behaviour in adult subjects with ADHD | *Design*: Case-control study  *Country:* Switzerland  *Setting:* Correctional facility  *Follow-up:* NA | *N:* 13  *Gender:* M  *Age category:* Adult  *Diagnosis:* ADHD  *Offender type:* Mixed offender types  *Controls:* M (13): Non-offenders with ADHD, M (26): Healthy controls | *Outcome measure:* Brain activity  *Assessment method:* EEG  *Function:* Etiologic  *Stimulus/task:* Modified visual Go-NoGo continuous performance task (VCPT) and a newly developed version of the visual CPT that additionally requires emotional evaluation (ECPT) | ADHD symptomatology may be a risk factor for delinquency since some neural information processing deficits found in ADHD seemed to be even more pronounced in offenders with ADHD. Results suggest additional risk factors consisting of deviant higher-order visual processing, especially of facial affect, as well as abnormalities in monitoring and evaluative functions of response inhibition |
| Meier et al. (2014) | To investigate whether delinquent behavior in adults with ADHD symptomatology is related to excessive beta (brain) activity | *Design*: Case-control study  *Country:* Switzerland  *Setting:* Correctional facility  *Follow-up:* NA | *N:* 13  *Gender:* M  *Age category:* Adult  *Diagnosis:* ADHD  *Offender type:* Mixed offender types  *Controls:* M (13): Non-offenders with ADHD, M (26): Healthy controls | *Outcome measure:* Brain activity  *Assessment method:* EEG  *Function:* Etiologic  *Stimulus/task:* NA (resting-state EEG) | Delinquents with ADHD symptomatology showed more beta power at frontal, central, and parietal brain regions than non-delinquents with ADHD symptoms. Excessive beta power may thus represent a risk factor for delinquent behavior in adults with ADHD symptomatology |
| Mier et al. (2014) | To investigate the neurobiological correlates of social-cognitive related alterations in psychopathy | *Design*: Case-control study  *Country:* Germany  *Setting:* Inpatient clinic  *Follow-up:* NA | *N:* 11  *Gender:* M  *Age category:* Adult  *Diagnosis:* Psychopathy  *Offender type:* Mixed offender types  *Controls:* M (18): Healthy controls | *Outcome measure:* Brain activity  *Assessment method:* fMRI  *Function:* Etiologic  *Stimulus/task:* Affective ToM, emotion recognition, and neutral face processing tasks | Psychopaths had reduced brain activation related to face processing. Related to affective ToM, psychopaths had hypoactivation in several brain areas associated with embodied simulation of emotions and intentions and they lacked connectivity between superior temporal sulcus and the amygdala during affective ToM. This might provide the neural substrate of reduced feeling with others during social cognition |
| Miner et al. (1995) | To compare sexual preference for child and aggressive stimuli in rapists and child molesters using auditory and visual stimuli | *Design*: Case-control study  *Country:* USA  *Setting:* Inpatient clinic  *Follow-up:* NA | *N:* 38  *Gender:* M  *Age category:* Adult  *Diagnosis:* Not explicitly stated  *Offender type:* Sex offenders: Adult victims  *Controls:* M (78): Child molesters with female victim, M (38): Child molesters with male victim | *Outcome measure:* Change in penis circumference  *Assessment method:* PPG  *Function:* Etiologic  *Stimulus/task:* Audio and visual (deviant) sexual stimuli | Child molesters with male victims showed a more offense related arousal profile than both other groups, and rapists responded significantly more to rape and non-sexual assault than the child molester groups |
| Montry et al. (2021) | To investigate whether psychopathic traits in offenders are associated with electrophysiological anomalies during a phonological/phonetic decision task that places differential demands on left hemisphere language processing systems | *Design*: Cross-sectional  *Country:* USA  *Setting:* Correctional facility  *Follow-up:* NA | *N:* 43  *Gender:* M  *Age category:* Adult  *Diagnosis:* Psychopathy/psychopathic traits  *Offender type:* NS  *Controls:* NA | *Outcome measure:* Brain activity  *Assessment method:* EEG  *Function:* Etiologic  *Stimulus/task:* Non-target stimuli during a phological/phonetic decision task | Results provided physiological evidence that psychopathic traits are related to reduced language lateralization at the phonological level of word processing |
| Motzkin et al. (2011) | To assess structural and functional connectivity of ventromedial prefrontal cortex (vmPFC) in psychopathic and non-psychopathic criminals using diffusion tensor imaging and fMRI respectively | *Design*: Case-control study  *Country:* USA  *Setting:* Correctional facility  *Follow-up:* NA | *N:* 20  *Gender:* M  *Age category:* Adult  *Diagnosis:* Psychopathy  *Offender type:* NS  *Controls:* M (20): Offenders, non-psychopathic | *Outcome measure:* Brain activity  *Assessment method:* fMRI  *Function:* Etiologic  *Stimulus/task:* NA (resting-state fMRI) | Psychopathy is associated with reduced structural integrity in the right uncinate fasciculus, the primary white matter connection between vmPFC and anterior temporal lobe, and also with reduced connectivity between vmPFC and amygdala as well as between vmPFC and medial parietal cortex. Data implicate diminished vmPFC connectivity as a characteristic neurobiological feature of psychopathy |
| Moulden et al. (2009) | To explore the role of paedophilia in the prediction of recidivism | *Design*: Cohort study  *Country:* Canada  *Setting:* Outpatient clinic  *Follow-up:* 20 years | *N:* 206  *Gender:* M  *Age category:* Adult  *Diagnosis:* Paedophilia  *Offender type:* Sex offenders: Child victims  *Controls:* NA | *Outcome measure:* Change in penis circumference  *Assessment method:* PPG  *Function:* Diagnostic  *Stimulus/task:* NA | Only phallometric assessment was associated with sexual recidivism (small effect size). Overall recidivism rates were 22.8%, 33.9%, and 45.6% for sexual, violent, and any reoffence, respectively. No differences were found between paedophiles and non-paedophiles with respect to recidivism rates, regardless of how paedophilia was defined. The utility of the diagnosis of paedophilia for the purpose of predicting future reoffending is questioned |
| Moulier et al. (2012) | To investigate whether in a paedophilic man attracted to boys brain responses to pictures representing boys would be changed by leuprorelin, a GnRH agonist | *Design*: Quasi-experimental, longitudinal study  *Country:* France  *Setting:* Outpatient clinic  *Follow-up:* 5 months | *N:* 1  *Gender:* M  *Age category:* Adult  *Diagnosis:* Paedophilia  *Offender type:* Sex offenders: Child victims  *Controls:* M (1): Healthy control | *Outcome measure:* Change in penis circumference, brain activity  *Assessment method:* PPG, fMRI  *Function:* Monitoring  *Stimulus/task:* (Non-) erotic/sexual pictures | Leuprorelin decreased activity in regions known to mediate the perceptual, motivational, and affective responses to visual sexual stimuli in a paedophilic sex offender |
| Müller et al. (2007) | To assess brain structure and function in psychopathic offenders using voxel-based morphometry (VBM) and fMRI respectively | *Design*: Case-control study  *Country:* Germany  *Setting:* Inpatient clinic  *Follow-up:* NA | *N:* 6  *Gender:* M  *Age category:* Adult  *Diagnosis:* Psychopathy  *Offender type:* NS  *Controls:* M (6): Offenders, non-psychopathic | *Outcome measure:* Brain activity  *Assessment method:* fMRI  *Function:* Etiologic  *Stimulus/task:* Viewing affective pictures from the IAPS | In criminal psychopaths highly significant gray matter loss in the right superior temporal gyrus was found, accompanied by emotion-related hypofunction |
| Müller et al. (2008) | To compare brain activity of criminal psychopaths and healthy controls when addressing emotion-cognition interaction | *Design*: Case-control study  *Country:* Germany  *Setting:* Inpatient clinic  *Follow-up:* NA | *N:* 10  *Gender:* M  *Age category:* Adult  *Diagnosis:* Psychopathy  *Offender type:* NS  *Controls:* M (12): Healthy controls | *Outcome measure:* Brain activity  *Assessment method:* fMRI  *Function:* Etiologic  *Stimulus/task:* Viewing and rating affective and neutral pictures from the IAPS and performing a cognitive task | Compared to healthy controls criminal psychopaths had impaired prefrontal and temporal brain function during emotion and cognition interaction |
| Müller et al. (2003) | To investigate the influence of affective contents on brain activation in criminal psychopaths | *Design*: Case-control study  *Country:* Germany  *Setting:* Inpatient clinic  *Follow-up:* NA | *N:* 6  *Gender:* M  *Age category:* Adult  *Diagnosis:* Psychopathy  *Offender type:* NS  *Controls:* M (6): Healthy controls | *Outcome measure:* Brain activity  *Assessment method:* fMRI  *Function:* Etiologic  *Stimulus/task:* Viewing pictures with positive and negative affective content from the IAPS | Psychopathy is neurobiologically reflected by dysregulation and disturbed functional connectivity of emotion-related brain regions |
| Müller et al. (2014) | To investigate whether patterns of penile tumescence (as a proxy for sexual interest) measured by penile plethysmography testing can change in men with paedophilic sexual interest | *Design*: Case series, retrospective study  *Country:* Germany  *Setting:* Inpatient clinic  *Follow-up:* 6 months | *N:* 43  *Gender:* M  *Age category:* Adult  *Diagnosis:* Paedophilia  *Offender type:* Sex offenders: Child victims  *Controls:* NA | *Outcome measure:* Change in penis circumference  *Assessment method:* PPG  *Function:* Monitoring  *Stimulus/task:* Paedophilic and non-paedophilic (sexual) stimuli | Sexual arousal as assessed with penile plethysmography can change over time in paedophiles |
| Muñoz et al. (2008) | To examine the interaction between verbal ability and the dimensions comprising psychopathic traits in predicting various indices of serious delinquency and in predicting psychophysiological measures (indexing emotional reactivity) | *Design*: Cross-sectional study  *Country:* USA  *Setting:* Correctional facility  *Follow-up:* NA | *N:* 100  *Gender:* M  *Age category:* Juvenile  *Diagnosis:* Juvenile psychopathic traits  *Offender type:* Mixed offender types  *Controls:* NA | *Outcome measure:* Skin conductance  *Assessment method:* Two electrodes placed on the middle two distal phalanges of the non-dominant hand  *Function:* Etiologic  *Stimulus/task:* Playing a competitive computer game with provocation levels | Callous-unemotional traits moderate the positive association between verbal abilities and violent delinquency; also high CU traits are associated with low skin conductance reactivity during a provocation task |
| Muñoz et al. (2008) | To investigate differences in the behavioral and psychophysiological responses to provocation and in the level of callous-unemotional traits in boys exhibiting different patterns of aggression | *Design*: Cross-sectional study  *Country:* USA  *Setting:* Correctional facility  *Follow-up:* NA | *N:* 85  *Gender:* M  *Age category:* Juvenile  *Diagnosis:* Juvenile psychopathic traits  *Offender type:* Mixed offender types  *Controls:* NA | *Outcome measure:* Skin conductance  *Assessment method:* Two electrodes placed on the middle two distal phalanges of the non-dominant hand  *Function:* Etiologic  *Stimulus/task:* Playing a competitive computer game with provocation levels | Adolescents who show reactive aggression show high levels of retaliatory responses to relatively lower levels of provocation. Adolescents with both reactive and proactive aggression are distinct in showing high rates of aggressive responses, even in the absence of provocation, and do not show the same increase in aggressive responses to relatively low levels of provocation. These adolescents who are also high on callous-unemotional traits appear to show low levels of reactivity on physiological measures, despite their behavioral responses to provocation |
| Munro et al. (2007) | To determine whether violent offenders, assumedly low in socialization, would show impaired error monitoring and produce a diminished error-related negativity, both suggestive of diminished anterior cingulate cortex function | *Design*: Case-control study  *Country:* Canada  *Setting:* Inpatient clinic  *Follow-up:* NA | *N:* 15  *Gender:* M  *Age category:* Adult  *Diagnosis:* Psychopathy  *Offender type:* Violent offenders  *Controls:* M (15): Healthy controls | *Outcome measure:* Brain activity  *Assessment method:* EEG  *Function:* Etiologic  *Stimulus/task:* Standard letter flanker task and face flanker task that required discrimination between angry and fearful expressions | Psychopathy is associated with an atypical response when error monitoring requires the discrimination of affectively based information |
| Munro et al. (2007) | To investigate response inhibition in psychopathic violent offenders | *Design*: Case-control study  *Country:* Canada  *Setting:* Inpatient clinic  *Follow-up:* NA | *N:* 15  *Gender:* M  *Age category:* Adult  *Diagnosis:* Psychopathy  *Offender type:* Violent offenders  *Controls:* M (15): Healthy controls | *Outcome measure:* Brain activity  *Assessment method:* EEG  *Function:* Etiologic  *Stimulus/task:* Go-NoGo task | Neural processes involved in response inhibition are not abnormal in psychopaths when both stimuli and context are affectively neutral |
| Newman et al. (2010) | To examine fear-potentiated startle in a sample of incarcerated psychopathic and non-psychopathic offenders under experimental conditions that focused attention directly on fear-relevant information or established an alternative attentional focus under low or high cognitive load | *Design*: Cross-sectional study  *Country:* USA  *Setting:* Correctional facility  *Follow-up:* NA | *N:* 125  *Gender:* M  *Age category:* Adult  *Diagnosis:* Psychopathy/psychopathic traits  *Offender type:* NS  *Controls:* NA | *Outcome measure:* Fear potentiated startle  *Assessment method:* EMG  *Function:* Etiologic  *Stimulus/task:* Fear conditioning task (letter cues) with electric shocks | Psychopaths’ diminished reactivity to fear stimuli, and emotion-related cues more generally reflect idiosyncrasies in attention that limit their processing of peripheral information |
| Nijman et al. (2023) | To investigate whether music in a preferred genre and ordered in a specific sequence to maximize the beneficial arousal reducing properties of music (X-System) has an effect on physiological and psychological variables in a sample of staff members and inpatients in forensic psychiatry | *Design*: Randomized cross-over trial (AB, BA), longitudinal study  *Country:* The Netherlands  *Setting:* Inpatient clinic  *Follow-up:* 48 hours | *N:* 18  *Gender:* M (15) + F (3)  *Age category:* Adult  *Diagnosis:* Mixed diagnoses  *Offender type:* NS  *Controls:* M (6) - F (14): Staff members | *Outcome measure:* Skin conductance, heart rate, temperature, movement  *Assessment method:* Empatica 4 wristband  *Function:* Etiologic  *Stimulus/task:* Listening to music | The selection of a preferred music genre led to a reduction in physiological arousal. Although there was a trend that this reduction was greater when the participants had listened to the X-System playlist this difference was not statistically significant |
| Nummenmaa et al. (2021) | To compare structural and functional brain characteristics of violent psychopathic offenders, matched healthy controls, and normally functioning individuals with high psychopathic traits from a community sample | *Design*: Case-control study  *Country:* Finland  *Setting:* Correctional facility  *Follow-up:* NA | *N:* 19  *Gender:* M  *Age category:* Adult  *Diagnosis:* Psychopathy  *Offender type:* Violent offenders  *Controls:* M (19): Healthy controls, M (49) - F (51): Healthy controls | *Outcome measure:* Brain activity  *Assessment method:* EEG  *Function:* Etiologic  *Stimulus/task:* Viewing movie clips with varying violent content | Normally functioning individuals with high psychopathic traits have structural and functional brain characteristics that are similar to violent offenders with high psychopathic traits. These characteristics include both fronto-limbic cortical atrophy and enhanced brain activity in affective circuits while seeing violence |
| Ó Ciardha (2010) | To compare results of a pictorial modified Stroop task and penile plethysmography in child molesters (paedophiles) and rapists | *Design*: Case-control study  *Country:* USA  *Setting:* Inpatient clinic  *Follow-up:* NA | *N:* 20  *Gender:* M  *Age category:* Adult  *Diagnosis:* Paedophilia  *Offender type:* Sex offenders: Child victims  *Controls:* M (20): Sex offenders with adult victim | *Outcome measure:* Change in penis circumference  *Assessment method:* PPG  *Function:* Diagnostic  *Stimulus/task:* Audio and visual (deviant) sexual stimuli | Both PPG and the pictorial modified Stroop task were able to discriminate between offenders based on their gender of victim but could not differentiate between child molesters and rapists |
| Ogloff and Wong (1990) | To investigate electrodermal and cardiovascular substrates of coping mechanisms in the presence of impeding aversive stimulation in psychopathic offenders | *Design*: Case-control study  *Country:* Canada  *Setting:* Inpatient clinic  *Follow-up:* NA | *N:* 32 (participants + controls)  *Gender:* M  *Age category:* Adult  *Diagnosis:* Psychopathy  *Offender type:* NS  *Controls:* M (NS): Inpatients, low psychopathy | *Outcome measure:* Skin conductance, heart rate  *Assessment method:* Beckman Dynograph and Cardiotachometer  *Function:* Etiologic  *Stimulus/task:* Countdown task and audio stimulus (tone) | Psychopaths show lower levels of arousal to impeding aversive stimuli and thus they may employ an effective coping response in anticipation of such stimuli |
| Okasha et al. (1975) | To investigate EEG abnormalities in Egyptian murderers | *Design*: Case-control study  *Country:* Egypt  *Setting:* Correctional facility and inpatient clinic  *Follow-up:* NA | *N:* 90  *Gender:* M (NS) +F (NS)  *Age category:* Adult  *Diagnosis:* Mixed diagnoses  *Offender type:* Violent offenders  *Controls:* M - F (30): Healthy controls | *Outcome measure:* Brain activity  *Assessment method:* EEG  *Function:* Etiologic  *Stimulus/task:* NA (resting-state EEG) | The incidence of EEG abnormality in the prison and inpatient groups was substantial, with a very high incidence among prisoners whose crime was apparently motiveless, and a higher incidence in psychotics than psychopaths |
| Ostrosky-Solís et al. (2008) | Extensive neuropsychological, electrophysiological, and neuropsychiatric testing of a female serial killer | *Design*: Case study  *Country:* Mexico  *Setting:* Correctional facility  *Follow-up:* NA | *N:* 1  *Gender:* F  *Age category:* Adult  *Diagnosis:* Psychopathy  *Offender type:* Violent offender  *Controls:* NA | *Outcome measure:* Brain activity, ERP  *Assessment method:* EEG  *Function:* Etiologic  *Stimulus/task:* Resting state EEG and ERPs while viewing pictures of emotionally charged unpleasant scenes with and without moral content as well as emotionally pleasant and neutral pictures | In the female serial killer, dysfunctions of the amygdala and frontal cortex were found that were linked to psychopathic impairment in emotional processing and elevated levels of reactive violent behavior |
| Pardini and Phillips (2010) | To examine neural responses to several emotional cues in chronically violent men and explore the association between neural responses to social emotions and psychopathic features | *Design*: Case-control study  *Country:* USA  *Setting:* Community  *Follow-up:* NA | *N:* 22  *Gender:* M  *Age category:* Adult  *Diagnosis:* Psychopathy  *Offender type:* Violent offenders  *Controls:* M (20): Healthy controls | *Outcome measure:* Brain activity  *Assessment method:* fMRI  *Function:* Etiologic  *Stimulus/task:* Visual stimuli: Happy, sad, angry, fearful and neutral faces | Chronically violent men exhibit a reduced neural response to facial cues regardless of emotional content. It appears that they may view emotionally ambiguous facial cues as potentially threatening and implicitly reinterpret subtle cues of fear in others so they no longer elicit a negative response |
| Pastor et al. (2003) | To examine startle probe modulation during affective picture viewing in a Spanish prison population | *Design*: Case-control study  *Country:* Spain  *Setting:* Correctional facility  *Follow-up:* NA | *N:* 18  *Gender:* M  *Age category:* Adult  *Diagnosis:* Psychopathy  *Offender type:* Mixed offender types  *Controls:* M (20): Offenders, mediocre psychopathy score, M (10): Offenders, non-psychopathic | *Outcome measure:* Skin conductance, eyeblinks, heart rate response  *Assessment method:* Skin conductance coupler, EMG, ECG  *Function:* Etiologic  *Stimulus/task:* Affective picture viewing | Diminished defense activation characterizes psychopaths despite cultural differences |
| Patrick et al. (1993) | To examine emotional, including physiological, responses to pleasant, neutral, and unpleasant visual stimuli in a sample of convicted sex offenders | *Design*: Case-control study  *Country:* USA  *Setting:* Inpatient clinic  *Follow-up:* NA | *N:* 18  *Gender:* M  *Age category:* Adult  *Diagnosis:* Psychopathy  *Offender type:* Sex offenders: Victim not specified  *Controls:* M (18): Inpatients, mediocre psychopathy score, M (18): Inpatients, non-psychopathic | *Outcome measure:* Skin conductance, blink response, heart rate response, facial electromyographic activity  *Assessment method:* Skin conductance coupler, EMG, ECG  *Function:* Etiologic  *Stimulus/task:* Affective picture viewing | Study results suggest an abnormality in the processing of emotional stimuli by psychopaths that manifests itself independently of affective report |
| Patrick et al. (1994) | To investigate physiological response mobilization accompanying imagery of emotional situations in psychopathic sex offenders | *Design*: Case-control study  *Country:* USA  *Setting:* Inpatient clinic  *Follow-up:* NA | *N:* 17  *Gender:* M  *Age category:* Adult  *Diagnosis:* Psychopathy  *Offender type:* Sex offenders: Victim not specified  *Controls:* M (18): Inpatients, low psychopathy Factor 1 and 2, M (18): Inpatients, low psychopathy Factor 1 and high psychopathy Factor 2 | *Outcome measure:* Skin conductance, heart rate, electromyographic activity  *Assessment method:* Beckman Type RM Dynograph  *Function:* Etiologic  *Stimulus/task:* Subjects imagined fearful and neutral scenes in a cued sentence-processing task | Low psychopathy subjects showed larger physiological reactions during fearful imagery than high psychopathy subjects indicating that in the latter, semantic and emotional processes are dissociated |
| Pezzoli et al. (2021) | To investigate whether increased prefrontal brain activity induced by acute anodal versus sham transcranial direct current stimulation could reduce the automatic attention bias for child versus adult images in sex offenders against children with paedophilic disorder | *Design*: Non-RCT (matched control group), longitudinal study  *Country:* USA  *Setting:* Outpatient clinic  *Follow-up:* 25 minutes | *N:* 16  *Gender:* M  *Age category:* Adult  *Diagnosis:* Paedophilia  *Offender type:* Sex offenders: Child victims  *Controls:* M (16): Healthy controls | *Outcome measure:* Eye movement  *Assessment method:* Eye tracker  *Function:* Intervention  *Stimulus/task:* Performing a task requiring controlled attention to computer‐generated images of clothed and nude children and adults | The study results suggest enhanced cognitive control in response to salient stimuli during active tDCS in paedophilic child sex offenders. However, no attentional bias and no tDCS effects on attentional responses to child and adult images emerged following tDCS |
| Pfabigan et al. (2015) | To investigate affective and cognitive empathic processes in incarcerated violent offenders with lower and higher psychopathic traits and healthy controls | *Design*: Case-control study  *Country:* Austria  *Setting:* Correctional facility  *Follow-up:* NA | *N:* 14  *Gender:* M  *Age category:* Adult  *Diagnosis:* Psychopathy  *Offender type:* Violent offenders  *Controls:* M (15): Offenders, low psychopathy, M (14): Healthy controls | *Outcome measure:* Skin conductance  *Assessment method:* Bioamplifier with skin conductance sensor  *Function:* Etiologic  *Stimulus/task:* Viewing painful expressions of others displayed on video clips | Violent offenders displayed reduced autonomic arousal in response to distress cues of others, irrespective of psychopathy. However, only higher psychopathic-trait offenders were able to provide self-report in a way that let them appear to be as empathic as controls; enabling them to know, yet not to feel, what others feel |
| Pham et al. (2000) | To compare psychopathic and non-psychopathic detained offenders in terms of their physiological and subjective responses to video clips depicting different emotions | *Design*: Case-control study  *Country:* Belgium  *Setting:* Correctional facility  *Follow-up:* NA | *N:* 14  *Gender:* M  *Age category:* Adult  *Diagnosis:* Psychopathy  *Offender type:* NS  *Controls:* M (16): Offenders, non-psychopathic | *Outcome measure:* Electrodermal activity, heart rate, systolic and diastolic blood pressure, mean blood pressure, skin temperature, muscle tension  *Assessment method:* Finapress 2300, Vitaport  *Function:* Videoclips depicting different emotions: Joy, fear, anger, sadness, and disgust others displayed on video clips | Besides a lower blood pressure before and during emotional stimulation, no specific psychophysiological deficits were found in psychopathic offenders |
| Philippi et al. (2015) | To examine whether psychopathy is associated with alterations in functional connectivity in three large-scale cortical networks | *Design*: Case-control study  *Country:* USA  *Setting:* Correctional facility  *Follow-up:* NA | *N:* 46  *Gender:* M  *Age category:* Adult  *Diagnosis:* Psychopathy  *Offender type:* NS  *Controls:* M (49): Offenders, non-psychopathic | *Outcome measure:* Brain activity  *Assessment method:* fMRI  *Function:* Etiologic  *Stimulus/task:* NA (resting-state fMRI) | Connectivity between cortical association hubs, such as the dorsal anterior cingulate cortex, may be a neurobiological marker of psychopathy |
| Plaud and Gaither (1997) | To investigate whether the clinical effects of covert sensitization, a behavior therapy technique for sexual deviations, may (at least partly) directly involve habituation processes | *Design*: Case, longitudinal study  *Country:* USA  *Setting:* Outpatient clinic  *Follow-up:* 90 days | *N:* 1  *Gender:* M  *Age category:* Adult  *Diagnosis:* Not explicitly stated  *Offender type:* Sex offender: Child victims  *Controls:* NA | *Outcome measure:* Change in penis circumference  *Assessment method:* PPG  *Function:* Monitoring  *Stimulus/task:* Sexually explicit audiotapes | Habituation processes could be involved in the beneficial clinical effects usually noted in covert sensitization procedures |
| Poeppl et al. (2011) | To investigate the underlying neural processing of sexual interest among paedophiles | *Design*: Case-control study  *Country:* USA  *Setting:* Inpatient clinic  *Follow-up:* NA | *N:* 9  *Gender:* M  *Age category:* Adult  *Diagnosis:* Paedophilia  *Offender type:* Sex offenders: Child victims  *Controls:* M (11): Non-sex offenders | *Outcome measure:* Brain activity  *Assessment method:* fMRI  *Function:* Etiologic  *Stimulus/task:* Viewing pictures of nude (prepubescents, pubescents, and adults) and neutral content, as well as performing a concomitant choice reaction time task | The brain response of paedophiles to visual sexual stimulation by images of nude prepubescents is comparable with previously described neural patterns of sexual processing in non-paedophilic human males evoked by visual stimuli depicting nude adults. Nevertheless, group differences found in the cingulate gyrus and the insular region suggest an important role of these brain areas in paedophilic sexual interest |
| Ponseti et al. (2017) | To investigate if paedophilia is linked to an over-active nurturing system by exposing paedophiles and healthy controls to pictures of infant and adult animals during functional magnetic resonance imaging of the brain | *Design*: Case-control study  *Country:* Germany  *Setting:* Outpatient clinic  *Follow-up:* NA | *N:* 60  *Gender:* M  *Age category:* Adult  *Diagnosis:* Paedophilia  *Offender type:* Sex offenders: Child victims  *Controls:* M (55): Healthy controls | *Outcome measure:* Brain activity  *Assessment method:* fMRI  *Function:* Etiologic  *Stimulus/task:* Pictures of infant and adult animals | Results suggest an over-responsive nurturing system in paedophilia in addition to or instead of an aberrant sexual system |
| Ponseti et al. (2012) | To investigate whether hemodynamic brain response to sexual stimuli can accurately identify paedophiles | *Design*: Case-control study  *Country:* Germany  *Setting:* Outpatient clinic  *Follow-up:* NA | *N:* 24  *Gender:* M  *Age category:* Adult  *Diagnosis:* Paedophilia  *Offender type:* Sex offenders: Child victims  *Controls:* M (32): Healthy controls | *Outcome measure:* Brain activity  *Assessment method:* fMRI  *Function:* Diagnostic  *Stimulus/task:* Sex images of nude children and adults | Functional brain response patterns to sexual stimuli contain sufficient information to identify paedophiles with high accuracy |
| Ponseti et al. (2016) | To investigate whether paedophilia can be inferred based on the hemodynamic brain responses to adult and child faces | *Design*: Case-control study  *Country:* Germany  *Setting:* Outpatient clinic  *Follow-up:* NA | *N:* 24  *Gender:* M  *Age category:* Adult  *Diagnosis:* Paedophilia  *Offender type:* Sex offenders: Child victims  *Controls:* M (32): Healthy controls | *Outcome measure:* Brain activity  *Assessment method:* fMRI  *Function:* Diagnostic  *Stimulus/task:* Images of child and adult, male, and female faces | Study results indicate that the functional response to facial stimuli can be reliably used for fMRI-based classification of paedophilia, bypassing the problem of showing child sexual stimuli to paedophiles |
| Pontius (1999) | To suggest an explanatory mechanism for three puzzling cases of fire setting by previously nondestructive boys | *Design*: Case-control study  *Country:* USA  *Setting:* Inpatient clinic  *Follow-up:* NA | *N:* 3  *Gender:* M  *Age category:* Juvenile  *Diagnosis:* Other  *Offender type:* Other  *Controls:* M (15) - F (5): Offenders, mixed diagnoses | *Outcome measure:* Brain activity  *Assessment method:* EEG  *Function:* Diagnostic  *Stimulus/task:* NA | The three boys' consistent symptomatology was very similar to that reported for 17 men with bizarre homicidal acts implicating a kindled partial seizure called 'Limbic Psychotic Trigger Reaction' |
| Pontius (2001) | To illustrate 'diagnostic overshadowing' by two cases of unplanned, motiveless bank robbery, initially merely attributed to 'antisocial or 'schizoid/avoidant' (loner) personality disorder, respectively | *Design*: Case study  *Country:* USA  *Setting:* Inpatient clinic  *Follow-up:* NA | *N:* 2  *Gender:* M  *Age category:* Adults  *Diagnosis:* Other  *Offender type:* Other  *Controls:* NA | *Outcome measure:* Brain activity  *Assessment method:* EEG  *Function:* Diagnostic  *Stimulus/task:* NA | In case of the two bank robbers, their personality disorders initially tended to overshadow the unobservable counterparts in the form of partial seizures: Temporal Lobe Epilepsy and a proposed Limbic Psychotic Trigger Reaction, respectively |
| Popma et al. (2006) | To investigate the relationship between disruptive behavior in male adolescents and their HPA and autonomic reactivity to a standard public speaking task | *Design*: Case-control study  *Country:* The Netherlands  *Setting:* Correctional facility  *Follow-up:* NA | *N:* 71  *Gender:* M  *Age category:* Juvenile  *Diagnosis:* Disruptive behavior disorder  *Offender type:* NS  *Controls:* M (30): Healthy controls | *Outcome measure:* Skin conductance, heart rate  *Assessment method:* VU-AMS  *Function:* Etiologic  *Stimulus/task:* Public speaking task | Low cortisol and HR responsivity to stress may be a neurobiological marker for delinquent boys with DBD, but not for those without DBD |
| Porter and Feldman (2011) | To report on a case of factitious paedophilia | *Design*: Case study  *Country:* USA  *Setting:* Inpatient clinic  *Follow-up:* 21 years | *N:* 14  *Gender:* M  *Age category:* Adult  *Diagnosis:* Other  *Offender type:* Sex offenders: Child victims  *Controls:* NA | *Outcome measure:* Change in penis circumference  *Assessment method:* PPG  *Function:* Diagnostic  *Stimulus/task:* Audio and visual (deviant) sexual stimuli | This case enlarges the literature on factitious psychological disorders and shows that some individuals may falsify paraphilic behaviors, although clearly minimization of these behaviors remains more common |
| Prado et al. (2021) | To report on a case of paedophilic disorder in the setting of frontotemporal dementia | *Design*: Case, longitudinal study  *Country:* Brazil  *Setting:* Outpatient clinic  *Follow-up:* 4 years | *N:* 1  *Gender:* M  *Age category:* Adult  *Diagnosis:* Paedophilia  *Offender type:* Sex offender: Child victims  *Controls:* NA | *Outcome measure:* Brain activity  *Assessment method:* SPECT  *Function:* Diagnostic  *Stimulus/task:* NA | In this case, preexisting developmental paedophilia was “unmasked” by underlying progressive frontotemporal degeneration |
| Prehn et al. (2013) | To examine how emotion governs decision making within two subgroups of antisocial criminal offenders with either emotional hypo- or hyper-reactivity compared with healthy, non-criminal controls | *Design*: Case-control study  *Country:* Germany  *Setting:* Correctional facility and inpatient clinic  *Follow-up:* NA | *N:* 11  *Gender:* M  *Age category:* Adult  *Diagnosis:* Psychopathy/psychopatic traits, antisocial personality disorder  *Offender type:* Violent offenders  *Controls:* M (12): Offenders, psychopathy Factor 2 and low psychopathy Factor 1, M (13): Healthy controls | *Outcome measure:* Brain activity  *Assessment method:* fMRI  *Function:* Etiologic  *Stimulus/task:* Financial decision-making task | The data indicate that emotionally hypo-reactive offenders (with psychopathic traits) constitute a special subgroup within antisocial offenders characterized in particular by a limited capacity to emotionally represent uncertainty and to anticipate punishment |
| Prehn et al. (2013) | To investigate the influence of concurrently presented emotional stimuli on cognitive task processing in violent criminal offenders primarily characterized by affective instability | *Design*: Case-control study  *Country:* Germany  *Setting:* Correctional facility and inpatient clinic  *Follow-up:* NA | *N:* 15  *Gender:* M  *Age category:* Adult  *Diagnosis:* Antisocial personality disorder  *Offender type:* Violent offenders  *Controls:* M (17): Healthy controls | *Outcome measure:* Brain activity  *Assessment method:* fMRI  *Function:* Etiologic  *Stimulus/task:* Working memory task (run 1) and working memory task during which emotional, neutral, low, or high salient social scenes in the background are shown (run 2) | Study results illustrate an interaction of emotion and cognition in affective instable individuals with enhanced reactivity to emotionally salient stimuli which might be an important factor regarding the understanding of their aggressive and violent behaviour |
| Proulx et al. (1993) | To verify the efficacy of a semantic tracking task in reducing the magnitude of voluntary control homosexual paedophiles, who are not familiar with PPG, exert on their penile responses | *Design*: RCT study  *Country:* Canada  *Setting:* Inpatient clinic  *Follow-up:* NA | *N:* 12  *Gender:* M  *Age category:* Adult  *Diagnosis:* Paedophilia  *Offender type:* Sex offender: Child victims  *Controls:* M (12): Child sex offenders | *Outcome measure:* Change in penis circumference  *Assessment method:* PPG  *Function:* Diagnostic  *Stimulus/task:* Watching non-deviant sexual stimuli (videotape) and listening to deviant (paedophilic) sexual stimuli (audtiotape) while carrying out a semantic tracking task | The use of a semantic tracking task increased the internal validity of phallometry in homosexual paedophiles, especially among those with prior experience in phallometry |
| Proulx et al. (1993) | To verify the efficacy of a semantic tracking task in reducing the magnitude of voluntary control homosexual paedophiles, who are familiar with PPG, exert on their penile responses | *Design*: Randomized cross-over trial (AB, BA) study  *Country:* Canada  *Setting:* Inpatient clinic  *Follow-up:* NA | *N:* 12  *Gender:* M  *Age category:* Adult  *Diagnosis:* Paedophilia  *Offender type:* Sex offenders: Child victims  *Controls:* M (12): Child sex offenders | *Outcome measure:* Change in penis circumference  *Assessment method:* PPG  *Function:* Diagnostic  *Stimulus/task:* Watching non-deviant sexual stimuli (videotape) and listening to deviant (paedophilic) sexual stimuli (audtiotape) while carrying out a semantic tracking task | The use of a semantic tracking task increased the internal validity of phallometry in homosexual paedophiles, especially among those with prior experience in phallometry |
| Pujara et al. (2014) | To examine the neural correlates of reward and loss sensitivity in a group of criminal psychopaths | *Design*: Case-control study  *Country:* USA  *Setting:* Correctional facility  *Follow-up:* NA | *N:* 18  *Gender:* M  *Age category:* Adult  *Diagnosis:* Psychopathy  *Offender type:* NS  *Controls:* M (23): Offenders, non-psychopathic | *Outcome measure:* Brain activity  *Assessment method:* fMRI  *Function:* Etiologic  *Stimulus/task:* Performing a task involving the gain or loss of money | Although psychopaths and non-psychopaths did not significantly differ with respect to overall levels of ventral striatum response to reward versus loss, significantly different correlations between ventral striatum responses and psychopathy severity were observed within each group |
| Pujol et al. (2012) | To investigate whether alterations in the brain network subserving moral judgment in criminal psychopaths are not limited to the inadequate network use during moral judgment, but that a primary network breakdown exists with dysfunctional alterations outside moral dilemma situations | *Design*: Case-control study  *Country:* Spain  *Setting:* NS  *Follow-up:* NA | *N:* 22  *Gender:* M  *Age category:* Adult  *Diagnosis:* Psychopathy  *Offender type:* Violent offenders  *Controls:* M (22): Healthy controls | *Outcome measure:* Brain activity  *Assessment method:* fMRI  *Function:* Etiologic  *Stimulus/task:* fMRI during a moral dilemma task, attention-demanding (Stroop) task, and resting-state | The network subserving moral judgment is underactive in psychopathic individuals during moral dilemma situations, but results also provided evidence of a baseline network alteration outside moral contexts with a functional disconnection between emotional and cognitive elements that jointly construct moral judgment |
| Quinsey et al. (1976) | To investigate changes of sexual preference in child molesters assessed with physiological and verbal measures during aversion therapy | *Design*: Cohort study  *Country:* Canada  *Setting:* Inpatient clinic  *Follow-up:* Not explicitly stated | *N:* 10  *Gender:* M  *Age category:* Adult  *Diagnosis:* Mixed diagnoses  *Offender type:* Sex offender: Child victims  *Controls:* NA | *Outcome measure:* Change in penis circumference, skin conductance  *Assessment method:* PPG, skin conductance measurement device  *Function:* Monitoring  *Stimulus/task:* Visual (deviant) sexual stimuli | Larger skin conductance responses were made to child slides than to adult slides in all sessions. Penile circumference responses indicated a small positive shift in sexual preference in the assessment tests when averaged over subjects. Pre- and post-treatment semantic differential and slide ranking sexual preference measures also showed increases in sexual preference for adults relative to sexual preference for children |
| Quinsey et al. (1975) | To investigate penile circumference, skin conductance, and ranking responses of child molesters and 'normals' to sexual and non-sexual visual stimuli | *Design*: Case-control study  *Country:* Canada  *Setting:* Inpatient clinic  *Follow-up:* NA | *N:* 20  *Gender:* M  *Age category:* Adult  *Diagnosis:* Mixed diagnoses  *Offender type:* Sex offenders: Child victims  *Controls:* M (11): Patients, non-sex offender, M (10): Healthy controls | *Outcome measure:* Change in penis circumference, skin conductance  *Assessment method:* PPG, Beckman Biopotential Skin Electrodes  *Function:* Diagnostic  *Stimulus/task:* Visual sexual and non-sexual stimuli (slides) | Penile plethysmography, but not skin conductance response nor the ranking test, could differentiate child molesters from the control groups |
| Raine et al. (2000) | To examine prefrontal gray matter volume and autonomic activity in antisocial personality disorder | *Design*: Case-control study  *Country:* USA  *Setting:* Community  *Follow-up:* NA | *N:* 21  *Gender:* M  *Age category:* Adult  *Diagnosis:* Psychopathy, antisocial personality disorder  *Offender type:* Violent offenders  *Controls:* M (34): Healthy controls, M (26): Individuals with substance abuse disorder | *Outcome measure:* Skin conductance, heart rate  *Assessment method:* Polygraph  *Function:* Etiologic  *Stimulus/task:* Social stressor: Participants gave a videotaped speech of their faults | The antisocial personality disorder group showed reduction in prefrontal gray matter volume and reduced autonomic autonomic activity during the stressor |
| Raine (1988) | To investigate whether psychopaths have a heightened ability to attend to task relevant events or that they are unable to sustain attention to such events | *Design*: Case-control study  *Country:* UK  *Setting:* Correctional facility  *Follow-up:* NA | *N:* 14  *Gender:* M  *Age category:* Adult  *Diagnosis:* Psychopathy  *Offender type:* NS  *Controls:* M (14): Offenders, non-psychopathic | *Outcome measure:* Brain activity  *Assessment method:* EEG  *Function:* Etiologic  *Stimulus/task:* Continuous performance task (targeting digits) | Study results indicate proficient information-processing on parietal-related tasks which may underlie clinical reports of enhanced short-term attentional ability in psychopaths |
| Raine et al. (1999) | To investigate whether a subgroup of criminals with schizotypal personality would show skin conductance orienting deficits and increased alcoholism | *Design*: Cohort study  *Country:* Denmark  *Setting:* Community  *Follow-up:* 15 years | *N:* 134  *Gender:* M  *Age category:* Adult  *Diagnosis:* Schizotypical personality disorder, substance use disorder  *Offender type:* NS  *Controls:* NA | *Outcome measure:* Skin conductance  *Assessment method:* Beckman Dynograph  *Function:* Etiologic  *Stimulus/task:* Audio stimuli (tones) | It is argued that schizotypal criminals are a relatively distinct group and that prefrontal dysfunction may underlie both orienting deficits and alcoholism in this group |
| Raine (1987) | To investigate associations between psychopathy and schizotypy measures and electrodermal and cognitive variables in a sample of prisoners | *Design*: Cross-sectional study  *Country:* UK  *Setting:* Correctional facility  *Follow-up:* NA | *N:* 36  *Gender:* M  *Age category:* Adult  *Diagnosis:* Psychopathy, schizotypical personality disorder  *Offender type:* Mixed offender types  *Controls:* NA | *Outcome measure:* Skin conductance  *Assessment method:* Beckman Skin conductance coupler  *Function:* Etiologic  *Stimulus/task:* Audio stimuli (tones) | Ratings of psychopathy are unrelated to skin conductance orienting response frequency, whereas measures of schizotypy (especially the Anhedonia-Psychoticism component) are |
| Raskin and Hare (1978) | To investigate the effectiveness of detection of deception with polygraph examination in a mock crime in psychopathic and non-psychopathic prisoners | *Design*: Case-control study  *Country:* USA  *Setting:* Correctional facility  *Follow-up:* NA | *N:* 24  *Gender:* M  *Age category:* Adult  *Diagnosis:* Psychopathy  *Offender type:* NS  *Controls:* M (24): Offenders, non-psychopathic | *Outcome measure:* Skin conductance and potential, heart rate and vasomotor activity, thoracic and abdominal respiration  *Assessment method:* Polygraph (Beckman type R Dynograph with accessories)  *Function:* Diagnostic  *Stimulus/task:* Interview during polygraphic assessment | Psychopaths were as easily detected as non-psychopaths, and psychopaths showed evidence of stronger electrodermal responses and hearth rate decelerations |
| Renaud et al. (2010) | To compare the respective sexual arousal responses and the perceptual motor dynamics of sexual aggressors against children and non-deviant subjects | *Design*: Case-control study  *Country:* Canada  *Setting:* Inpatient clinic  *Follow-up:* NA | *N:* 13  *Gender:* M  *Age category:* Adult  *Diagnosis:* Paedophilia  *Offender type:* Sex offenders: Child victims  *Controls:* M (29): Healthy controls | *Outcome measure:* Change in penis circumference, gaze behaviour  *Assessment method:* PPG, motion tracker and oculomotor tracking system  *Function:* Diagnostic  *Stimulus/task:* Visual neutral and sexual stimuli using virtual characters | Perceptual-motor fractal dynamics parallels sexual arousal and differs from paedophiles to non-deviant subjects when critical sexual information is processed |
| Reyes et al. (2014) | To demonstrate the usefulness of performing neurophysiological and neuropsychological assessments in patients, in order to demonstrate the significant role of the emotions in the execution of certain behaviours | *Design*: Case study  *Country:* Cuba  *Setting:* Inpatient clinic  *Follow-up:* NA | *N:* 1  *Gender:* M  *Age category:* Adult  *Diagnosis:* Other  *Offender type:* Violent offender  *Controls:* NA | *Outcome measure:* Brain activity  *Assessment method:* EEG  *Function:* Diagnostic  *Stimulus/task:* NA (wake EEG) | EEG abnormalities were observed among other things. Neurophysiological assessment is seen as an added value in the forensic psychiatric assessment of criminal suspects because it can increase the objectivity of diagnostics |
| Reyes and Amador (2009) | To investigate qualitative and quantitative EEG abnormalities in violent offenders with and without antisocial personality disorder | *Design*: Case-control study  *Country:* Cuba  *Setting:* Inpatient clinic  *Follow-up:* NA | *N:* 18  *Gender:* M  *Age category:* Adult  *Diagnosis:* Antisocial personality disorder  *Offender type:* Violent offenders  *Controls:* M (10): Offenders, no ASPD | *Outcome measure:* Brain activity  *Assessment method:* EEG  *Function:* Etiologic  *Stimulus/task:* NA (resting-state EEG) | High incidences of EEG abnormalities were found in both groups of violent offenders. These abnormalities were more severe in offenders with antisocial personality disorder |
| Reyes et al. (2017) | To adapt a commonly used paired-item preference assessment format to evaluate age and gender preferences for sex offenders with intellectual disability | *Design*: Cross-sectional study  *Country:* USA  *Setting:* Inpatient clinic  *Follow-up:* NA | *N:* 5  *Gender:* M  *Age category:* Adult  *Diagnosis:* Other: Intellectual disability  *Offender type:* Sex offenders: Child victims  *Controls:* NA | *Outcome measure:* Change in penis circumference  *Assessment method:* PPG  *Function:* Etiologic  *Stimulus/task:* Deviant (males and females under the age of 18) and non-deviant (males and females over the age of 18) video clips | Preference assessment methodology may be used as an assessment component for intellectually disabled sex offenders to identify grade or age and gender preferences |
| Reyes et al. (2006) | To investigate deviant sexual arousal in adult male child sex offenders with developmental disabilities | *Design*: Cross-sectional study  *Country:* USA  *Setting:* Inpatient clinic  *Follow-up:* NA | *N:* 10  *Gender:* M  *Age category:* Adult  *Diagnosis:* Other: Intellectual disability  *Offender type:* Sex offenders: Child victims  *Controls:* NA | *Outcome measure:* Change in penis circumference  *Assessment method:* PPG  *Function:* Etiologic  *Stimulus/task:* Neutral and (deviant) sexual videoclips | Three general patterns of arousal outcomes were obtained: differentiated deviant arousal, undifferentiated deviant arousal, and no deviant arousal. It is concluded that the methodological approach taken in this study highlights a potential contribution of single-subject methodology for the assessment of sex offenders with developmental disabilities |
| Reyes et al. (2011) | To replicate and extend an existing method for the assessment of sexual arousal in child sex offenders via penile plethysmograph during exposure to deviant, non- deviant, and neutral stimuli | *Design*: Multiple case studies  *Country:* USA  *Setting:* Inpatient clinic  *Follow-up:* NA | *N:* 3  *Gender:* M  *Age category:* Adult  *Diagnosis:* Other: Intellectual disability  *Offender type:* Sex offenders: Child victims  *Controls:* NA | *Outcome measure:* Change in penis circumference  *Assessment method:* PPG  *Function:* Etiologic  *Stimulus/task:* Neutral and (deviant) sexual videoclips | Three general patterns of arousal outcomes were obtained: differentiated deviant arousal, undifferentiated deviant arousal, and no deviant arousal. None of the arousal outcomes suggested evidence of habituation across repeated exposures to the stimuli, and therefore provide additional support for the utility of repeated measurement assessments for this population |
| Rice and Harris (2002) | To examine the characteristics and recidivism rates of child molesters who had offended against their genetic daughters, stepdaughters, or unrelated girls and to investigate the predictive value of two actuarial risk instruments | *Design*: Case-control, longitudinal study  *Country:* Canada  *Setting:* Inpatient clinic and outpatient clinic  *Follow-up:* Mean follow-up time: 53.6 months | *N:* 82  *Gender:* M  *Age category:* Adult  *Diagnosis:* Not explicitly stated  *Offender type:* Sex offenders: Child victims  *Controls:* M (102): Child sex offenders with extrafamilial victim | *Outcome measure:* Change in penis circumference  *Assessment method:* PPG  *Function:* Etiologic  *Stimulus/task:* Audio and visual sexual stimuli | Men who offended against their own daughters had less deviant sexual age preferences and were less likely to commit new violent and sexual offenses. However, they exhibited an average absolute phallometric preference for prepubertal children and had a violent recidivism rate of 22% in a follow-up of less than 5 years |
| Rice et al. (2012) | To examine phallometric responses of adolescents who had sexually offended and comparing their profiles and preference indices with those of matched adult sex offenders and 'normals' | *Design*: Case-control, longitudinal study  *Country:* Canada  *Setting:* Inpatient clinic and outpatient clinic  *Follow-up:* 56.9 months | *N:* 61  *Gender:* M  *Age category:* Juvenile  *Diagnosis:* Not explicitly stated  *Offender type:* Sex offenders: Child victims  *Controls:* M (69): Adult sex offenders with child victim, M (NS): Non-offenders or non-sex offenders | *Outcome measure:* Change in penis circumference  *Assessment method:* PPG  *Function:* Etiologic  *Stimulus/task:* Several sets of audio and/or visual sexual stimuli | Phallometry has valid clinical and research uses with adolescent males who commit serious sex offenses |
| Ristow et al. (2019) | To investigate brain activation during anticipation of sexually relevant cues in paedophilic sex offenders | *Design*: Case-control study  *Country:* Germany  *Setting:* Inpatient clinic  *Follow-up:* NA | *N:* 13  *Gender:* M  *Age category:* Adults  *Diagnosis:* Paedophilia  *Offender type:* Sex offenders: Child victims  *Controls:* M (13): Healthy controls | *Outcome measure:* Brain activity  *Assessment method:* fMRI  *Function:* Etiologic  *Stimulus/task:* Visual stimuli: Erotic, positive-emotional, and neutral photographs depicting males and females from the IAPS | Paedophilic sex offenders have decreased brain activation to adult cues |
| Ristow et al. (2019) | To investigate brain activation during anticipation of sexually relevant cues in paedophilic sex offenders | *Design*: Case-control study  *Country:* Germany  *Setting:* Inpatient clinic  *Follow-up:* NA | *N:* 13  *Gender:* M  *Age category:* Adults  *Diagnosis:* Paedophilia  *Offender type:* Sex offenders: Child victims  *Controls:* M (13): Healthy controls | *Outcome measure:* Brain activity  *Assessment method:* fMRI  *Function:* Etiologic  *Stimulus/task:* Visual stimuli: Erotic, positive-emotional, and neutral photographs depicting both males and females from the IAPS and photographs depicting whole-body frontal views of either a naked adult or a naked child in randomized order | Paedophilic sex offenders have decreased brain activation to adult cues and preference specificity in neural response during expectancy of erotic stimuli |
| Rodman et al. (2016) | To map the unique variance associated with externalizing and psychopathy to well-characterized brain circuitry for interference suppression and response inhibition | *Design*: Cross-sectional study  *Country:* USA  *Setting:* Correctional facility  *Follow-up:* NA | *N:* 46  *Gender:* M  *Age category:* Adult  *Diagnosis:* Psychopathy/psychopatic traits  *Offender type:* NS  *Controls:* NA | *Outcome measure:* Brain activity  *Assessment method:* fMRI  *Function:* Etiologic  *Stimulus/task:* Eriksen flanker task that incorporated a Go-NoGo manipulation | Results provide neurobiological evidence supporting the fractionation of antisocial behavior and identify dissociable mechanisms through which different facets predispose dysfunction and impairment |
| Rosburg et al. (2018) | To investigate whether event related potentials can help to clarify whether child sexual offenders show an altered processing of stop signals and commission errors | *Design*: Case-control study  *Country:* Switzerland  *Setting:* NS  *Follow-up:* NA | *N:* 21  *Gender:* M  *Age category:* Adults  *Diagnosis:* Paedophilia  *Offender type:* Sex offenders: Child victims  *Controls:* M (19), Non-contact child sex offenders, M (21): Healthy controls | *Outcome measure:* ERP  *Assessment method:* EEG  *Function:* Etiologic  *Stimulus/task:* Go-NoGo task | Response inhibition, processing of stop signals, and error detection are not necessarily impaired in child sexual offenders. However, they appear to dedicate less cognitive resources to the evaluation of committed errors |
| Rosemberger et al. (2019) | To investigate whether psychopathic offenders would violate fairness norms during a repeated trust game because of increased profit-maximizing concerns | *Design*: Case-control study  *Country:* Austria  *Setting:* Correctional facility  *Follow-up:* NA | *N:* 25  *Gender:* M  *Age category:* Adult  *Diagnosis:* Psychopathy/psychopatic traits  *Offender type:* NS  *Controls:* M (23): Healthy controls | *Outcome measure:* Skin conductance  *Assessment method:* Bioamplifier with skin conductance sensor  *Function:* Etiologic  *Stimulus/task:* Playing a trust game | Fairness norm violations were positively associated with Factor 2 scores (the lifestyle/anti-social subscale) of the PCL-R, but this was not accompanied by clear profit-maximizing behaviour. In addition, anticipatory arousal to self-advantageous decisions was higher in all offenders, independent of their degree of psychopathy, compared with non-offenders |
| Rothemund et al. (2012) | To examine peripheral and central correlates of fear conditioning in high psychopathic offenders using unconditioned and conditioned stimuli (i.e., painful shock and neutral face images) | *Design*: Case-control study  *Country:* Germany  *Setting:* Correctional facility  *Follow-up:* NA | *N:* 11  *Gender:* M  *Age category:* Adult  *Diagnosis:* Psychopathy  *Offender type:* NS  *Controls:* M (11): Healthy controls | *Outcome measure:* ERP, skin conductance, startle response potentiation, heart rate, corrugator activity  *Assessment method:* EEG, bioamplifier, EMG, ECG  *Function:* Etiologic  *Stimulus/task:* A painful electric stimulus served as unconditioned stimulus and neutral faces as conditioned stimuli | Results indicate a deficient capacity to form associations between neutral and aversive events in psychopathy that appears unrelated to cognitive deficits and is consistent with hypothesized frontolimbic deficits in the disorder |
| Sadeh and Verona (2012) | To investigate whether attentional and emotional processes and their interactive effects are important for understanding distinct psychopathic deficits | *Design*: Cross-sectional study  *Country:* USA  *Setting:* Correctional facility  *Follow-up:* NA | *N:* 63  *Gender:* M (52) + F (11)  *Age category:* Adult  *Diagnosis:* Psychopathy  *Offender type:* NS  *Controls:* NA | *Outcome measure:* ERP, fear potentiated startle  *Assessment method:* EEG, EMG  *Function:* Etiologic  *Stimulus/task:* Visual stimuli: neutral and unpleasant IAPS stimuli that were matched on visual complexity | Psychopathy is not solely characterized by an emotional or attentional deficit, but by interactive cognition-emotion deficits that manifest differentially across the psychopathy dimensions |
| Saleh (2005) | To present the effects of leuprolide acetate (leuprolide) in combination with psychotherapy on sexual functioning in a young male sex offender | *Design*: Case study  *Country:* USA  *Setting:* Inpatient clinic  *Follow-up:* 5 months | *N:* 1  *Gender:* M  *Age category:* Adult  *Diagnosis:* Paraphilia  *Offender type:* Sex offender: Other  *Controls:* NA | *Outcome measure:* Change in penis circumference  *Assessment method:* PPG  *Function:* Monitoring  *Stimulus/task:* Audio trials narrating various types of sexual activities | A positive treatment effect is reported |
| Salley et al. (1980) | To clarify the role of REM sleep in psychopathy | *Design*: Case-control study  *Country:* USA  *Setting:* Correctional facility  *Follow-up:* NA | *N:* 17  *Gender:* M  *Age category:* Adult  *Diagnosis:* Psychopathy  *Offender type:* NS  *Controls:* M (6): Offenders, non-psychopathic | *Outcome measure:* Sleep stages (brain activity)  *Assessment method:* EEG  *Function:* Etiologic  *Stimulus/task:* NA | No significant differences were found in the sleep patterns of the subgroups; the psychopaths with abnormal EEG's tended to have the highest REM time and REM% of the inmates, contrary to Hare's theory |
| Sartorius et al. (2008) | To investigate the amygdala activation profile in paedophile sex-offenders | *Design*: Case-control study  *Country:* Germany  *Setting:* Inpatient clinic  *Follow-up:* NA | *N:* 10  *Gender:* M  *Age category:* Adult  *Diagnosis:* Paedophilia  *Offender type:* Sex offender: Child victims  *Controls:* M (10): Healthy controls | *Outcome measure:* Brain activity  *Assessment method:* fMRI  *Function:* Etiologic  *Stimulus/task:* Visual stimuli: Images of men, women, boys or girls randomly embedded in neutral target/non-target geometrical symbols | Reduced emotional arousal for children relative to adults is reversed in paedophilia, suggesting a neural substrate associated with deviant sexual preference in this condition |
| Sayed et al. (1969) | To investigate legally insane murderers using psychiatric assessment and EEG | *Design*: Case-control study  *Country:* Scotland  *Setting:* Inpatient clinic  *Follow-up:* NA | *N:* 32  *Gender:* M  *Age category:* Adult  *Diagnosis:* Psychopathy, schizophrenia, other  *Offender type:* Violent offenders  *Controls:* M (32): Staff members | *Outcome measure:* Brain activity  *Assessment method:* EEG  *Function:* Etiologic  *Stimulus/task:* EEG assessment during rest state, hyperventilation, eye opening and closing, and intermittent photic stimulation by a stroboscope with eyes closed | The forensic patients had an incidence of EEG abnormality about four times that of the control group; approximately half the psychopaths had abnormal EEG's but the psychotic group had a much higher incidence |
| Schalling et al. (1973) | To examine spontaneous fluctuations and levels in skin conductance and finger pulse volume recordings in criminal subjects during three experimental periods: before, during, and after a series of auditory stimuli, and related to psychopathy | *Design*: Case-control study  *Country:* Sweden  *Setting:* Inpatient clinic  *Follow-up:* NA | *N:* 12  *Gender:* M  *Age category:* Adult  *Diagnosis:* Psychopathy  *Offender type:* Violent offenders  *Controls:* M (11): Offenders, below median psychopathy scores | *Outcome measure:* Skin conductance, finger pulse volume, finger temperature  *Assessment method:* Constant current ultra linear active resistance bridge, piezo-electric pressure transducer, thermistor  *Function:* Etiologic  *Stimulus/task:* Auditory stimuli | The more psychopathic group had a lower number of skin conductance fluctuations in stimulation and post-stimulation periods, and had lower levels, decreasing over periods, indicating lower cortical arousal. No differences were obtained in the pulse volume measurements |
| Scheeff et al. (2021) | To investigate early and late processing of emotional stimuli in a sample of incarcerated offenders with psychopathic traits while manipulating the attentional focus | *Design*: Case-control study  *Country:* Germany  *Setting:* Correctional facility  *Follow-up:* NA | *N:* 27  *Gender:* M  *Age category:* Adult  *Diagnosis:* Psychopathy/psychopathic traits  *Offender type:* Violent offenders  *Controls:* M (27): Healthy controls | *Outcome measure:* Brain activity  *Assessment method:* EEG  *Function:* Etiologic  *Stimulus/task:* Perceptual judgment task, and rating neutral and emotional stimuli regarding valence and arousal task | Results are indicative of a modulatory impact of attention on affective information processing in male violent offenders with psychopathic traits |
| Schenk et al. (2014) | To examine the differences in juvenile sex offenders who admitted bestiality based upon a self-report measure, compared to information elicited by polygraphs | *Design*: Cross-sectional study  *Country:* USA  *Setting:* Inpatient clinic  *Follow-up:* NA | *N:* 32  *Gender:* M  *Age category:* Juvenile  *Diagnosis:* Not explicitly stated  *Offender type:* Sex offenders: Victim not specified  *Controls:* NA | *Outcome measure:* Physiological indicators NOS  *Assessment method:* Polygraph  *Function:* Etiologic  *Stimulus/task:* Interview during polygraphic assessment | Results highlight the underreporting of bestiality on self-report measures and support the potential utility of using polygraph examinations to verify information |
| Schienle et al. (2017) | To investigate the neuronal basis of personal space intrusion in high-risk violent offenders | *Design*: Case-control study  *Country:* Austria  *Setting:* Correctional facility  *Follow-up:* NA | *N:* 17  *Gender:* M  *Age category:* Adult  *Diagnosis:* Psychopatic traits, antisocial personality disorder  *Offender type:* Violent offenders  *Controls:* M (18): Healthy controls | *Outcome measure:* Brain activity  *Assessment method:* fMRI  *Function:* Etiologic  *Stimulus/task:* Viewing of static and animated photos of neutral facial expressions by men and women | Insular sensitivity to reductions of personal distance within the study sample was increased, which possibly indexes their hostile attribution bias |
| Schiffer et al. (2009) | To analyze the brain activation pattern in response to sexual stimuli in an incarcerated paedophilic child sex offender treated with a long-acting LH-RH agonist | *Design*: Case study  *Country:* Germany  *Setting:* Inpatient clinic  *Follow-up:* 9 months | *N:* 1  *Gender:* M  *Age category:* Adult  *Diagnosis:* Paedophilia  *Offender type:* Sex offender: Child victims  *Controls:* NA | *Outcome measure:* Brain activity  *Assessment method:* fMRI  *Function:* Monitoring  *Stimulus/task:* Sexually arousing visual stimuli alternating with neutral visual stimuli | Treatment of paedophiles with an LH-RH agonist and the consequent strong decrease in testosterone levels may suppress the neuronal processing of sexual stimuli primarily in subcortical brain areas which are known to be significantly modulated by sex steroids, while other brain areas, in contrast, remain almost unaffected |
| Schiffer et al. (2008) | To analyze whether brain activation patterns of homosexual paedophiles differ from those of a non-paedophilic homosexual control group during visual sexual stimulation | *Design*: Case-control study  *Country:* Germany  *Setting:* Inpatient clinic  *Follow-up:* NA | *N:* 11  *Gender:* M  *Age category:* Adult  *Diagnosis:* Paedophilia  *Offender type:* Sex offenders: Child victims  *Controls:* M (12): Healthy controls | *Outcome measure:* Brain activity  *Assessment method:* fMRI  *Function:* Etiologic  *Stimulus/task:* Visual stimuli: Sexually stimulating photographs and emotionally neutral photographs | Central processing of visual sexual stimuli in homosexual paedophiles seems to be comparable to that in non-paedophile control subjects. However, compared with homosexual control subjects, activation patterns in paedophiles refer more strongly to subcortical regions, which have been discussed in the context of processing reward signals and also play an important role in addictive and stimulus-controlled behaviour |
| Schiffer et al. (2008) | To identify paedophilia specific functional networks implicated in sexual arousal | *Design*: Case-control study  *Country:* Germany  *Setting:* Inpatient clinic  *Follow-up:* NA | *N:* 8  *Gender:* M  *Age category:* Adult  *Diagnosis:* Paedophilia  *Offender type:* Sex offenders: Child victims  *Controls:* M (12): Healthy controls | *Outcome measure:* Brain activity  *Assessment method:* fMRI  *Function:* Etiologic  *Stimulus/task:* Visual stimuli: Sexually stimulating photographs and emotionally neutral photographs | Central processing of sexual stimuli in heterosexual paedophiles may be altered by a disturbance in the prefrontal networks, which may be associated with stimulus-controlled behaviors, such as sexually compulsive behaviors. This may suggest a dysfunction (in the functional and effective connectivity) at the cognitive stage of sexual arousal processing |
| Schiffer et al. (2014) | To determine whether offenders with ASPD, relative to non-offenders, display dysfunction in the neural mechanisms underlying cognitive control and to assess the extent to which these dysfunctions are associated with psychopathic traits and trait impulsivity | *Design*: Case-control study  *Country:* Germany  *Setting:* Correctional facility and inpatient clinic  *Follow-up:* NA | *N:* 21  *Gender:* M  *Age category:* Adult  *Diagnosis:* Antisocial personality disorder  *Offender type:* Violent offenders  *Controls:* M (23): Non-offenders | *Outcome measure:* Brain activity  *Assessment method:* fMRI  *Function:* Etiologic  *Stimulus/task:* Non-verbal Stroop task | Offenders with ASPD display alterations in the neural mechanisms underlying cognitive control and those alterations relate, at least in part, to personality characteristics |
| Schiffer et al. (2017) | To determine whether schizophrenic violent offenders with and without conduct disorder and antisocial personality disorder differ in Theory of Mind and neural activations subsuming this task | *Design*: Case-control study  *Country:* Germany  *Setting:* Correctional facility and inpatient clinic  *Follow-up:* NA | *N:* 13  *Gender:* M  *Age category:* Adult  *Diagnosis:* Conduct disorder, antisocial personality disorder, schizophrenia  *Offender type:* Violent offenders  *Controls:* M (16): Offenders, schizophrenia and no CD/ASPD, M (18): Offenders, CD/ASPD and no severe mental illness, M (18): Non-offenders, schizophrenia, M (18): Healthy controls | *Outcome measure:* Brain activity  *Assessment method:* fMRI  *Function:* Etiologic  *Stimulus/task:* Adapted version of the Reading-the-Mind-in-the-Eyes Task (RMET) | Individuals with schizophrenia who commit violent crimes and who have a life-long history of antisocial behavior resemble non-mentally ill offenders with similar childhood onset of antisocial behavior as to relatively intact mentalizing performance and elevated cognition-related but reduced affect-related brain activation patterns as compared to men without CD/ASPD |
| Schneider et al. (2000) | To investigate cerebral regional activation involved in the processing of negative affect in individuals with antisocial personality disorder and healthy controls | *Design*: Case-control study  *Country:* Germany  *Setting:* Inpatient clinic  *Follow-up:* NA | *N:* 12  *Gender:* M  *Age category:* Adult  *Diagnosis:* Psychopathy, antisocial personality disorder  *Offender type:* NS  *Controls:* M (12): Healthy controls | *Outcome measure:* Brain activity  *Assessment method:* fMRI  *Function:* Etiologic  *Stimulus/task:* Odors as unconditioned stimuli and faces as conditioned stimuli | Although patients with antisocial personality disorder showed no general impairment in behavioral conditioning, they demonstrated divergent neuronal network activity in cortical (dorsolateral prefrontal) and subcortical (amygdala) areas relative to normal subjects |
| Schober et al. (2005) | To compare cognitive-behavioral psychotherapy with cognitive–behavioral psychotherapy augmented by leuprolide acetate for suppression of paedophilic behavior | *Design*: Quasi-experimental, one-group pretest-posttest and masked design (with intervention and placebo condition) study  *Country:* USA  *Setting:* Outpatient clinic  *Follow-up:* 24 months | *N:* 5  *Gender:* M  *Age category:* Adult  *Diagnosis:* Paedophilia  *Offender type:* Sex offenders: Child victims  *Controls:* NA | *Outcome measure:* Change in penis circumference, galvanic skin, cardiovascular and respiratory response  *Assessment method:* PPG, Polygraph  *Function:* Monitoring  *Stimulus/task:* Monarch adult projective audio visual set version 5: Audio and visual depictions of males and females comprising preschool and grammar school children, adolescents, and adults | Cognitive–behavioral psychotherapy augmented with leuprolide acetate significantly reduced paedophilic fantasies, urges, and masturbation; however, paedophilic interest did not change during 1 year of therapy. Deceptive responses by polygraph suggested that self-report was unreliable |
| Schug et al. (2011) | To investigate whether murderers with schizophrenia are characterized by more-pronounced resting EEG slowing in frontal and temporal regions relative to both non-violent schizophrenic patients and murderers without mental illness and to determine if any group differences in resting EEG represent characteristics of a distinct homicidal schizophrenic subgroup rather than the influences of general mental illness alone | *Design*: Case-control study  *Country:* China  *Setting:* Inpatient clinic  *Follow-up:* NA | *N:* 32  *Gender:* M (NS) + F (NS)  *Age category:* Adult  *Diagnosis:* Schizophrenia  *Offender type:* Violent offenders  *Controls:* M – F (31): Homicidal individuals, no mental disorder, M – F (14): Homicidal individuals, non-psychotic mental disorder, M – F (33): Patients, non-violent and schizophrenic, M (47): Healthy controls | *Outcome measure:* Brain activity  *Assessment method:* EEG  *Function:* Etiologic  *Stimulus/task:* NA | While limited EEG evidence is found for a biologically distinct subtype of violent schizophrenia, results do suggest biological factors which distinguish violent individuals with schizophrenia from their counterparts who are not violent |
| Schuler et al. (2022) | To explore the neural correlates of cognitive empathy in subjects with paedophilia with and without child sexual offending | *Design*: Case-control study  *Country:* Germany  *Setting:* Outpatient clinic  *Follow-up:* NA | *N:* 15  *Gender:* M  *Age category:* Adult  *Diagnosis:* Paedophilia  *Offender type:* Sex offenders: Child victims  *Controls:* M (15): Paedophiles without child sex offending, M (24): Healthy controls | *Outcome measure:* Brain activity  *Assessment method:* fMRI  *Function:* Etiologic  *Stimulus/task:* Cognitive empathy task using cartoon stories | Study findings provide evidence for altered neural processing of cognitive empathy in individuals with paedophilia and child sexual offending |
| Schultz et al. (2016) | To determine whether the relationship between anxiety and fear acquisition differs in (primary and secondary) psychopathic and non-psychopatic prisoners | *Design*: Case-control study  *Country:* USA  *Setting:* Correctional facility  *Follow-up:* NA | *N:* 19  *Gender:* M  *Age category:* Adult  *Diagnosis:* Psychopathy  *Offender type:* NS  *Controls:* M (31): Offenders, non-psychopathic | *Outcome measure:* Galvanic skin response  *Assessment method:* Biopac systems skin conductance module  *Function:* Etiologic  *Stimulus/task:* Pavlovian fear conditioning: Two neutral grey-scale male faces were used as conditioned stimuli, one face being paired with an unconditioned stimulus consisting of an electric shock | Study results contradict the low-fear model of psychopathy and suggest that the low fear observed for psychopaths in previous studies may be specific to secondary psychopaths |
| Seidel et al. (2013) | To investigate whether violent offenders show a general empathy deficit or specific deficits regarding the separate subcomponents emotion recognition, perspective taking, and affective responsiveness | *Design*: Case-control study  *Country:* Austria  *Setting:* Correctional facility  *Follow-up:* NA | *N:* 30  *Gender:* M  *Age category:* Adult  *Diagnosis:* Mixed diagnoses  *Offender type:* Violent offenders  *Controls:* M (30): Healthy controls | *Outcome measure:* Skin conductance  *Assessment method:* Bioamplifier with skin conductance sensor  *Function:* Etiologic  *Stimulus/task:* Performing emotion recognition, perspective taking, and affective responsiveness tasks | Mainly emotion recognition is deficient in violent offenders whereas the other components of empathy are rather unaffected |
| Serafim et al. (2009) | To compare the emotional response and level of anxiety of psychopathic murderers, non-psychopathic murderers, and non-psychopathic non-criminals | *Design*: Case-control study  *Country:* Brazil  *Setting:* Correctional facility  *Follow-up:* NA | *N:* 38  *Gender:* M  *Age category:* Adult  *Diagnosis:* Psychopathy  *Offender type:* Violent offenders  *Controls:* M (37): Offenders, non-psychopathic, M (35): Healthy controls | *Outcome measure:* Heart rate  *Assessment method:* Dixtal Pulse Oximeter  *Function:* Etiologic  *Stimulus/task:* Viewing standardized pictures depicting pleasant, unpleasant and neutral content from the International Affective Picture System (IAPS) | Psychopathic murderers do not present variation in emotional response to different visual stimuli. Although the non-psychopathic murderers had committed the same type of crime as the psychopathic murderers, the former tended to respond with a higher level of anxiety and heart rate variation |
| Sergiou et al. (2022) | To examine high-definition transcranial direct current stimulation as an intervention to increase empathic abilities and reduce violent behavior in forensic substance-dependent offenders and examined the effects on the P3 and the late positive potential of the ERPs in reaction to situations that depict victims of aggression | *Design*: RCT study  *Country:* The Netherlands  *Setting:* Inpatient clinic  *Follow-up:* 1 week | *N:* 25  *Gender:* M  *Age category:* Adult  *Diagnosis:* Substance use disorder  *Offender type:* Violent offenders  *Controls:* M (37): Offenders, non-psychopathic, M (25): Inpatients, substance use disorder | *Outcome measure:* Brain activity  *Assessment method:* EEG  *Function:* Monitoring  *Stimulus/task:* Passive viewing empathy task: 95 pictures displaying scenes with either an aggressive (sexual, verbal or physical) interaction, or neutral interaction, or neutral object | Positive effects of HD-tDCS reducing aggression and modulate electrophysiological responses in forensic patients were found |
| Sergiou et al. (2023) | To investigate the effect of high-definition transcranial direct current stimulation (HD-tDCS) on frontal theta, alpha, and beta frequency power, asymmetrical frontal activity, and frontal synchronicity in violent offenders | *Design*: RCT study  *Country:* The Netherlands  *Setting:* Inpatient clinic  *Follow-up:* 1 week | *N:* 25  *Gender:* M  *Age category:* Adult  *Diagnosis:* Substance use disorder  *Offender type:* Violent offenders  *Controls:* M (37): Offenders, non-psychopathic, M (25): Inpatients, substance use disorder | *Outcome measure:* Brain activity  *Assessment method:* EEG  *Function:* Monitoring  *Stimulus/task:* NA (resting-state EEG task with eyes open and closed) | No evidence for a modulation effect of HD-tDCS on spectral power in the theta, alpha and beta frequency bands was found. However, left-frontal asymmetrical activity after HD-tDCS in the beta frequency band was increased, but only in a subsample of the forensic patients. Increased connectivity in frontal regions in the alpha and beta frequency bands as a result of HD-tDCS modulation was found, indicating enhanced synchronicity between frontal regions |
| Serin et al. (1994) | To investigate the relationship between psychopathy and deviant sexual arousal in a sample of sexual offenders with approximately equal numbers of rapists and child molesters | *Design*: Cross-sectional study  *Country:* Canada  *Setting:* Correctional facility  *Follow-up:* NA | *N:* 65  *Gender:* NS  *Age category:* Adult  *Diagnosis:* Psychopathy  *Offender type:* Sex offenders: Child and adult victims  *Controls:* NA | *Outcome measure:* Change in penis circumference  *Assessment method:* PPG  *Function:* Etiologic  *Stimulus/task:* Sexual stimuli | Psychopathy and phallometric indexes of deviant sexual arousal were significantly correlated. This relationship was most apparent for extrafamilial child molesters, somewhat less for rapists, and not at all for incest offenders. Rapists had higher psychopathy ratings than child molesters, approaching statistical significance |
| Serin et al. (2001) | A sample of incarcerated sexual was followed up post-release for 7 years to determine rates of recidivism, discriminant and predictive ability of psychopathy and sexual deviance, and degree of incremental predictive utility of grouping offenders based on extreme combinations of psychopathy and sexual deviance | *Design*: Cohort study  *Country:* Canada  *Setting:* Correctional facility  *Follow-up:* 7 years | *N:* 68  *Gender:* NS  *Age category:* Adult  *Diagnosis:* Psychopathy  *Offender type:* Sex offenders: Child and adult victims  *Controls:* NA | *Outcome measure:* Change in penis circumference  *Assessment method:* PPG  *Function:* Etiologic  *Stimulus/task:* NA | General recidivism and sex offender typologies are differentiated using information on psychopathy. Rapists and child molesters were differentiated based on measures of deviant sexual arousal. Those who displayed more psychopathic characteristics and deviant sexual arousal recidivated sooner and at significantly higher rates |
| Seto et al. (2000) | To examine the responses of adolescent sex offenders against children on a phallometric test of paedophilic interests | *Design*: Case-control study  *Country:* Canada  *Setting:* Outpatient clinic  *Follow-up:* NA | *N:* 115  *Gender:* NS  *Age category:* Adult and juvenile  *Diagnosis:* Paedophilia  *Offender type:* Sex offenders: Child and adult victims  *Controls:* NS (23): Rapists, NS (15): Non-offenders | *Outcome measure:* Change in penis circumference  *Assessment method:* PPG  *Function:* Diagnostic  *Stimulus/task:* Sexual stimuli | Phallometric testing can identify paedophilic interests among these adolescent sex offenders |
| Seto et al. (2004) | To replicate and extend earlier studies on the Screening Scale for Paedophilic Interests (SSPI) | *Design*: Cohort study  *Country:* Canada  *Setting:* Correctional facility  *Follow-up:* Mean duration: 60 months (study 1) and 64 months (study 2) | *N:* 113  *Gender:* M  *Age category:* Adult  *Diagnosis:* Not explicitly stated  *Offender type:* Sex offenders: Child victims  *Controls:* NA | *Outcome measure:* Change in penis circumference  *Assessment method:* PPG  *Function:* Diagnostic  *Stimulus/task:* Visual stimuli: Slides depicting prepubescent, pubescent, and adult male or female targets, as well as neutral landscape scenes | The SSPI was significantly, moderately, and positively correlated with a phallometric index of sexual arousal to prepubescent children in both samples. The SSPI showed evidence of predictive validity because it was significantly and positively correlated with violent recidivism in both samples and with sexual recidivism in the second sample |
| Shane and Groat (2018) | To investigate the extent to which offenders with varying PCL-R (psychopathy) scores could up- (or down-) regulate their neural response to negatively valent stimuli | *Design*: Case-control study  *Country:* USA  *Setting:* Correctional facility, inpatient clinic and outpatient clinic  *Follow-up:* NA | *N:* 15  *Gender:* M (14) + F (1)  *Age category:* Adult  *Diagnosis:* Psychopathy  *Offender type:* NS  *Controls:* M (22) - F (7): Offenders, medium psychopathy, M (20) - F (3): Offenders,low psychopathy | *Outcome measure:* Brain activity  *Assessment method:* fMRI  *Function:* Etiologic  *Stimulus/task:* Watching negatively- and neutrally-valent images naturally (passive-processing) or trying to increase or decrease emotional response to the images (instructed-processing) | Psychopathic individuals may be capable of manifesting emotional reactivity to negatively valent stimuli, at least under certain conditions |
| Siep et al. (2019) | To investigate brain connectivity using the amygdala as a region of interest before and after an emotion task in reactive aggressive violent offenders versus non-offender controls | *Design*: Case-control study  *Country:* The Netherlands  *Setting:* Inpatient clinic  *Follow-up:* NA | *N:* 19  *Gender:* M  *Age category:* Adult  *Diagnosis:* Mixed diagnoses  *Offender type:* Violent offenders  *Controls:* M (18): Non-offenders | *Outcome measure:* Brain activity  *Assessment method:* fMRI  *Function:* Etiologic  *Stimulus/task:* Resting state fMRI before and after emotion (anger and happiness) provocation or engagement task | Reactive aggression might stem from a focus on emotion processing, as indicated by an increase in limbic functional connectivity. The combination of a focus on emotion along with a lack of medial prefrontal cortex regulation has the potential to grow out of control e.g. in reactive aggression |
| Silverman (1943) | To investigate organic and psychogenic etiologic factors of psychopathy in a sample of criminal offenders | *Design*: Cross-sectional study  *Country:* USA  *Setting:* Inpatient clinic  *Follow-up:* NA | *N:* 75  *Gender:* M  *Age category:* Adult  *Diagnosis:* Psychopathy  *Offender type:* Mixed offender types  *Controls:* NA | *Outcome measure:* Brain activity  *Assessment method:* EEG  *Function:* Etiologic  *Stimulus/task:* NA | In 80% of the study sample EEG assessment showed (borderline) abnormalities and also 80% had psychologically unhealthy factors in childhood. Results suggest that psychopathic personality is a mental illness resulting from inborn or early acquired cerebral dysfunction and disturbed parent-child relationships |
| Simard et al. (2021) | To compare rest-related power spectral characteristics between two groups of offenders (with and without a cocaine-dependence diagnosis) and a non-offender control group | *Design*: Case-control study  *Country:* Canada  *Setting:* Correctional facility, inpatient clinic and outpatient clinic  *Follow-up:* NA | *N:* 37  *Gender:* M  *Age category:* Adult  *Diagnosis:* Substance use disorder  *Offender type:* NS  *Controls:* M (47); Offenders, no cocaïne use disorder, M (18): Non-offenders | *Outcome measure:* Brain activity  *Assessment method:* fMRI  *Function:* Etiologic  *Stimulus/task:* NA | Study results indicated broadly disrupted neural oscillations within both offender groups, compared to a group of non-offender controls |
| Sitaram et al. (2014) | To explore whether criminal psychopaths can learn volitional regulation of the left anterior insula with real-time fMRI neurofeedback | *Design*: One-group pretest-posttest design study  *Country:* Germany  *Setting:* Correctional facility  *Follow-up:* NA | *N:* 4  *Gender:* M  *Age category:* Adult  *Diagnosis:* Psychopathy  *Offender type:* Sex offenders: Victim not specified  *Controls:* NA | *Outcome measure:* Brain activity  *Assessment method:* fMRI  *Function:* Intervention  *Stimulus/task:* Employing negative emotional imageries taken from previous episodes the lives of the participants in conjunction with contingent feedback | Results showed modest feasibility that criminal psychopaths can learn self-regulation of a circumscribed brain area (anterior insula) with fMRI based Brain-Computer Interface, leading to modifications in the functional emotional network |
| Small (1966) | To relate the psychiatric, EEG, and other clinical attributes of 100 felons to various aspects of criminal behaviour | *Design*: Cross-sectional study  *Country:* USA  *Setting:* Inpatient clinic  *Follow-up:* NA | *N:* 100  *Gender:* M (94) + F (6)  *Age category:* Adult  *Diagnosis:* Mixed diagnoses  *Offender type:* Mixed offender types  *Controls:* NA | *Outcome measure:* Brain activity  *Assessment method:* EGG  *Function:* Etiologic  *Stimulus/task:* EEG assessment during waking, hyperventilation, photic stimulation, and sleep | A high incidence of mental illness, EEG abnormalities, and indications of central nervous system impairment was observed. No significant correlations were demonstrated between the psychiatric diagnoses, EEG findings, or results of other individual examinations and specific aspects of criminal behavior |
| Smith (1999) | To investigate neurophysiological processes associated with response inhibition in psychopathic and non-psychopathic inmates, stable, medicated schizophrenic outpatients, and healthy controls | *Design*: Case-control study  *Country:* Canada  *Setting:* Correctional facility and outpatient clinic  *Follow-up:* NA | *N:* 8  *Gender:* M  *Age category:* Adult  *Diagnosis:* Psychopathy  *Offender type:* NS  *Controls:* M (8): Offenders, non-psychopathic, M (9) - F (1): Outpatients, schizophrenic, M (8) - F (1): Healthy controls | *Outcome measure:* Brain activity  *Assessment method:* fMRI  *Function:* Etiologic  *Stimulus/task:* Go/No-Go task | Psychopathy is inversely correlated with dorsolateral prefrontal cortex activity |
| Smith and Sams (2005) | To investigate whether a quantitative EEG-based and an analog-based remedial neurofeedback training impact neurological and behavioral measures in adolescent offenders | *Design*: One-group pretest-posttest design study  *Country:* USA  *Setting:* Correctional facility  *Follow-up:* 6 months | *N:* 18  *Gender:* M (16) + F (2)  *Age category:* Juvenile  *Diagnosis:* Mixed diagnoses  *Offender type:* Mixed offender types  *Controls:* NA | *Outcome measure:* Brain activity  *Assessment method:* EGG  *Function:* Intervention  *Stimulus/task:* NA | EEG abnormalities and deficits in neuropsychological testing were found among offenders. Neurotherapy as an adjunctive treatment appears to hold promise for improvement in cognitive performance as well as recidivism |
| Smith et al. (1973) | To investigate EEG findings and any attendant abnormal personality correlates among baby batterers | *Design*: Cross-sectional study  *Country:* UK  *Setting:* NS  *Follow-up:* NA | *N:* 35  *Gender:* M (NS) + F (NS)  *Age category:* Adult  *Diagnosis:* Psychopathy, personality disorder  *Offender type:* Violent offenders  *Controls:* NA | *Outcome measure:* Brain activity  *Assessment method:* EGG  *Function:* Etiologic  *Stimulus/task:* NA | The presence of a definitely abnormal EEG in almost one quarter of the cases points to what may well be a separate subgroup to which special attention should be paid. This is further borne out by a demonstrable relation between personality diagnosis and abnormal EEG’s |
| Soderstrom et al. (2002) | To compare the regional cerebral blood flow (rCBF) in a group of violent offenders to scores on the Psychopathy Checklist-Revised (PCL-R) | *Design*: Cross-sectional study  *Country:* Sweden  *Setting:* Inpatient clinic  *Follow-up:* NA | *N:* 32  *Gender:* M (29) + F (3)  *Age category:* Adult and juvenile  *Diagnosis:* Mixed diagnoses  *Offender type:* Violent offenders  *Controls:* NA | *Outcome measure:* Brain activity  *Assessment method:* SPECT  *Function:* Etiologic  *Stimulus/task:* NA | Significant negative correlations were found between interpersonal features of psychopathy and frontal and temporal cerebral perfusion, which indicates that aberrant frontotemporal activity may be a factor in violent behaviour |
| Soderstrom et al. (2000) | To replicate previously reported findings of abnormal frontal and/or temporal cerebral blood flow in violent offenders and to control for the influence of major mental disorder, substance abuse, and current medication | *Design*: Cross-sectional study  *Country:* Sweden  *Setting:* Inpatient clinic  *Follow-up:* NA | *N:* 21  *Gender:* M (20) + F (1)  *Age category:* Adult and juvenile  *Diagnosis:* Mixed diagnoses  *Offender type:* Violent offenders  *Controls:* NA | *Outcome measure:* Brain activity  *Assessment method:* SPECT  *Function:* Etiologic  *Stimulus/task:* NA | In 16/21 subjects SPECT scans showed some hypoperfusion in the temporal and/or frontal lobes; no association was found with major mental disorder, substance abuse and medication use |
| Sommer et al. (2010) | To explore whether deficits in responding adequately to other people's emotion in psychopaths are associated with deficits in the ability to infer others' emotional states | *Design*: Case-control study  *Country:* Germany  *Setting:* Inpatient clinic  *Follow-up:* NA | *N:* 18  *Gender:* M  *Age category:* Adult  *Diagnosis:* Psychopathy  *Offender type:* Mixed offender types  *Controls:* M (14): Inpatients, non-psychopathic | *Outcome measure:* Brain activity  *Assessment method:* fMRI  *Function:* Etiologic  *Stimulus/task:* Emotional state assessment task using cartoons | Results emphasize that although psychopathic patients show no deficits in reasoning about other people's emotions if an explicit evaluation is demanded, they use divergent neural processing strategies that are related to more rational, outcome-oriented processes |
| Stafford-Clark and Taylor (1949) | To conduct a clinical and electro-encephalographic study of sixty-four prisoners charged with murder | *Design*: Cross-sectional study  *Country:* UK  *Setting:* Correctional facility  *Follow-up:* NA | *N:* 64  *Gender:* M (58) + F (6)  *Age category:* Adult and juvenile  *Diagnosis:* Mixed diagnoses  *Offender type:* Violent offenders  *Controls:* NA | *Outcome measure:* Brain activity  *Assessment method:* EGG  *Function:* Etiologic  *Stimulus/task:* NA | A significant correlation was found between apparently motiveless crime and EEG abnormality. EEG research can be a valuable addition to clinical research in criminal proceedings because the latter does not always provide a definitive answer to matters such as (in)sanity and responsibility for the crime committed |
| Stanford et al. (2007) | To test the hypothesis that when compared to non-violent controls the perpetrators of spousal/partner abuse would show problems in executive functioning and significantly lower P3 amplitude | *Design*: Case-control study  *Country:* USA  *Setting:* Correctional facility  *Follow-up:* NA | *N:* 18  *Gender:* M  *Age category:* Adult  *Diagnosis:* Mixed diagnoses  *Offender type:* Violent offenders  *Controls:* M (18): Healthy controls | *Outcome measure:* Brain activity  *Assessment method:* EGG  *Function:* Etiologic  *Stimulus/task:* Trail making test, Wisconsin card sorting test | Perpetrators of spousal/partner abuse show significant deficits in executive functioning, specifically impulse control, and lower P3 amplitude compared to non-violent controls. Aggressive behavior, at least within the context of spousal/partner abuse, may be partially explained by deficits in cognitive processing |
| Steele et al. (2016) | To investigate error-related processing (ERP) in adult male offenders with elevated psychopathic traits | *Design*: Cross-sectional study  *Country:* USA  *Setting:* Correctional facility  *Follow-up:* NA | *N:* 93  *Gender:* M  *Age category:* Adult  *Diagnosis:* Psychopathy/psychopathic traits  *Offender type:* NS  *Controls:* NA | *Outcome measure:* ERP  *Assessment method:* EGG  *Function:* Etiologic  *Stimulus/task:* Go-NoGo task | PCL-R total score, Factor 1 (interpersonal-affective traits), and Facet 3 (lifestyle traits) scores were positively related to post-error processes (i.e., increased Pe amplitude) but unrelated to error-monitoring processes (i.e., ERN/Ne). These results support the attentional bottleneck theory and further describe deficiencies related to elevated psychopathic traits that could be beneficial for new treatment strategies for psychopathy |
| Steele et al. (2015) | To evaluate prospective neuro-prediction of re-arrest by measuring post-error processing in both ERPs and functional magnetic resonance imaging (fMRI) | *Design*: Cohort study  *Country:* USA  *Setting:* Correctional facility  *Follow-up:* Mean follow-up time: 23.69 months | *N:* 45  *Gender:* M  *Age category:* Adult  *Diagnosis:* Substance use disorder  *Offender type:* NS  *Controls:* NA | *Outcome measure:* ERP and hemodynamic activity  *Assessment method:* EEG, fMRI  *Function:* Prognostic  *Stimulus/task:* Go-NoGo task | Both ERP and fMRI measures of error-processing prospectively predict rearrest |
| Steele et al. (2018) | To investigate whether functional network connectivity measures are predictive of substance abuse treatment completion using machine learning pattern classification of functional magnetic resonance imaging data | *Design*: Cohort study  *Country:* USA  *Setting:* Correctional facility  *Follow-up:* 12 weeks | *N:* 139  *Gender:* M (50) + F (89)  *Age category:* Adult  *Diagnosis:* Substance use disorder  *Offender type:* NS  *Controls:* NA | *Outcome measure:* Brain activity  *Assessment method:* fMRI  *Function:* Predictive  *Stimulus/task:* Experimental Go-NoGo task | Aberrant neural network connections predicted substance abuse treatment outcomes, which could illuminate new targets for developing interventions designed to reduce or eliminate substance use while facilitating long-term outcomes |
| Stinson and Becker (2008) | To examine and compare several assessment methods for sexual deviant interests and arousal among sex offenders, that rely on objective or physiological data, self-reported information from the offender, and historical behavior | *Design*: Cross-sectional study  *Country:* USA  *Setting:* Inpatient clinic  *Follow-up:* NA | *N:* 60  *Gender:* M  *Age category:* Adult  *Diagnosis:* Paraphilia  *Offender type:* Sex offenders: Child and adult victims  *Controls:* NA | *Outcome measure:* Change in penis circumference  *Assessment method:* PPG  *Function:* Diagnostic  *Stimulus/task:* Audio descriptions of both normative and non-normative sexual stimuli | Despite the general acceptance of objective or physiological measures of sexual interest and arousal in the literature as among the most accurate and reliable means of assessing these constructs, in this analysis they were only moderately if at all related to known sexual behaviors |
| Suchy et al. (2014) | To examine whether differences in performance speed and accuracy between paedophilic child molesters and non-paedophilic child molesters or other criminal and non-criminal controls reflect a slow/deliberate response style among paedophilics or a fundamental neuropathological weakness in processing speed | *Design*: Case-control study  *Country:* USA  *Setting:* Inpatient clinic  *Follow-up:* NA | *N:* 20  *Gender:* M  *Age category:* Adult  *Diagnosis:* Paedophilia  *Offender type:* Sex offenders: Child victims  *Controls:* M (20): Non-paedophilic child sex offenders, M (20): Non-sex offenders | *Outcome measure:* Change in penis circumference  *Assessment method:* PPG  *Function:* Diagnostic  *Stimulus/task:* Sexual stimuli (not stated if visual and/or acoustic) involving prepubescent children and adults | Paedophiles' slower performance is due to a fundamental neurocognitive weakness, rather than a slow/deliberate response style |
| Sun et al. (2022) | To compare neural responses to emotional communicative signals in incarcerated psychopathic offenders, individuals with autism spectrum disorder, and healthy controls | *Design*: Case-control study  *Country:* Finland  *Setting:* Correctional facility  *Follow-up:* NA | *N:* 19  *Gender:* M  *Age category:* Adult  *Diagnosis:* Psychopathy, antisocial personality disorder  *Offender type:* Violent offenders  *Controls:* M (20): High functioning ASD, M (19): Healthy controls | *Outcome measure:* Brain activity  *Assessment method:* fMRI  *Function:* Etiologic  *Stimulus/task:* Viewing dynamic happy, angry, and disgusted faces or listening to laughter and crying sounds | 'Mirroring' of vocal and facial emotional expressions was altered in both criminal offenders and participants with autism spectrum disorder and the somatosensory and motor responses to emotional signals were more reduced in the criminal offenders than in the ASD group |
| Sun et al. (2022) | To investigate functional connectivity alterations of the default mode network in male juvenile violent offenders | *Design*: Case-control study  *Country:* China  *Setting:* Correctional facility  *Follow-up:* NA | *N:* 31  *Gender:* M  *Age category:* Juvenile  *Diagnosis:* Mixed diagnoses  *Offender type:* Violent offenders  *Controls:* M (28): Healthy controls | *Outcome measure:* Brain activity  *Assessment method:* fMRI  *Function:* Etiologic  *Stimulus/task:* NA (resting-state fMRI) | The male juvenile violent offenders were associated with abnormal default mode network functional connectivity, which might be a neuroimaging basis for their tendency to violence |
| Sutton et al. (2002) | To examine emotional reactions in women with psychopathy in a mixed-picture paradigm using psychophysiological measures | *Design*: Case-control study  *Country:* USA  *Setting:* Correctional facility  *Follow-up:* NA | *N:* 24  *Gender:* F  *Age category:* Adult  *Diagnosis:* Psychopathy  *Offender type:* NS  *Controls:* F (94): Offenders, non-psychopathic, F (54): Offenders, psychopathic traits | *Outcome measure:* Skin conductance, facial electromyographic activity, hearth rate  *Assessment method:* Bioamplifier with skin conductance, electrocardiographic and EMG applications  *Function:* Etiologic  *Stimulus/task:* Visual stimuli (unpleasant, neutral, and pleasant pictures) and acoustic startle probes | As in men, one key component of psychopathy in women is abnormal emotion processing, especially in response to unpleasant or threatening stimuli |
| Syngelaki et al. (2013) | To examine emotion processing in male juvenile offenders and controls by measuring startle reflex responses to aversive sounds during the passive viewing of affective and neutral images | *Design*: Case-control study  *Country:* UK  *Setting:* Correctional facility  *Follow-up:* NA | *N:* 42  *Gender:* M  *Age category:* Juvenile  *Diagnosis:* Psychopathic traits, conduct disorder  *Offender type:* NS  *Controls:* M (52): Healthy controls | *Outcome measure:* Facial electromyographic activity  *Assessment method:* EMG  *Function:* Etiologic  *Stimulus/task:* Aversive sounds during the passive viewing of affective and neutral images | Antisocial children have a general defensive motivational system dysfunction and present with impairments in neural systems that subserve emotion processing and those with more severe conduct problems have reduced startle responses compared to those who are less severely affected |
| Szczypiński et al. (2022) | To examine differences in brain function and behavior between male patients with paedophilic disorder who commit child sexual abuse (CSO+) and who do not (CSO-), employing an affective Go-NoGo task during fMRI assessment | *Design*: Case-control study  *Country:* Poland  *Setting:* Inpatient clinic or outpatient clinic (not clear)  *Follow-up:* NA | *N:* 11  *Gender:* M  *Age category:* Adult  *Diagnosis:* Paedophilia  *Offender type:* Sex offenders: Child victims  *Controls:* M (14): Patients, no child sexual abuse, M (17): Healthy controls | *Outcome measure:* Brain activity  *Assessment method:* fMRI  *Function:* Etiologic  *Stimulus/task:* Affective Go-NoGo task | Impaired cognitive control in men with paedophilic disorder could be related to offender status rather than the sexual preference disorder |
| Tang et al. (2013) | To find regional coherence abnormalities in resting-state functional MRI of ASPD | *Design*: Case-control study  *Country:* China  *Setting:* Correctional facility  *Follow-up:* NA | *N:* 32  *Gender:* M  *Age category:* Adult  *Diagnosis:* Personality disorder  *Offender type:* NS  *Controls:* M (34): Offenders, no ASPD) | *Outcome measure:* Brain activity  *Assessment method:* fMRI  *Function:* Etiologic  *Stimulus/task:* NA | Compared with controls ASPD individuals show lower ReHo in the right cerebellum posterior lobe (Crus1) and the right middle frontal gyrus, as well as higher ReHo in the right middle occipital gyrus (BA 19), left inferior temporal gyrus (BA 37), and right inferior occipital gyrus (cuneus, BA 18) |
| Tang et al. (2013) | To design an exploratory data-driven classifier based on machine learning to investigate changes in functional connectivity in the brains of patients with antisocial personality disorder using resting-state fMRI data of ASPD patients and controls | *Design*: Case-control study  *Country:* China  *Setting:* Correctional facility  *Follow-up:* NA | *N:* 32  *Gender:* M  *Age category:* Adult  *Diagnosis:* Antisocial personality disorder  *Offender type:* Other  *Controls:* M (35): Offenders, no ASPD) | *Outcome measure:* Brain activity  *Assessment method:* fMRI  *Function:* Diagnostic and etiologic  *Stimulus/task:* NA (resting-state fMRI) | The classifier achieved satisfactory performance (86.57% accuracy, 77.14% sensitivity and 96.88% specificity) and could extract information regarding functional connectivity that could be used to discriminate ASPD individuals from normal controls |
| Tang et al. (2016) | To examine topological organization in resting-state fMRI data obtained from antisocial personality disorder patients and non-ASPD controls | *Design*: Case-control study  *Country:* China  *Setting:* Correctional facility  *Follow-up:* NA | *N:* 32  *Gender:* M  *Age category:* Adult  *Diagnosis:* Antisocial personality disorder  *Offender type:* Other  *Controls:* M (32): Offenders, no ASPD) | *Outcome measure:* Brain activity  *Assessment method:* fMRI  *Function:* Etiologic  *Stimulus/task:* NA (resting-state fMRI) | An aberrant topological organization of the functional brain network in individuals with ASPD is revealed and provides novel insights into the neuropathological mechanisms of ASPD |
| Ter Hamsel et al. (2021) | To explore whether a new technology-based biocueing intervention, the Sense-IT app, can be a valuable addition to aggression regulation treatment programs in forensic outpatient care | *Design*: One-group pretest-posttest design study  *Country:* The Netherlands  *Setting:* Outpatient clinic  *Follow-up:* 2 weeks | *N:* 10  *Gender:* M (9) + F (1)  *Age category:* Adult  *Diagnosis:* Not explicitly stated  *Offender type:* Mixed offender types  *Controls:* NA | *Outcome measure:* Hearth rate  *Assessment method:* Biosensor (smartwatches with photoplethysmography sensor)  *Function:* Intervention  *Stimulus/task:* NA | The evaluation study demonstrated moderate acceptability and adequate usability for the new version of the Sense-IT app. Exploratory analysis revealed a significant decrease in trait aggression postintervention, but no significant changes were found in other anger-related clinical outcomes |
| Ter Hamsel et al. (2023) | To assess the effects of the Sense-IT biocueing app as an addition to aggression regulation therapy (ART) on interoceptive awareness, emotion regulation, and aggressive behavior among forensic outpatients | *Design*: One-group pretest-posttest design, and multiple single case experimental design study  *Country:* The Netherlands  *Setting:* Outpatient clinic  *Follow-up:* 2 months | *N:* 25  *Gender:* M (23) + F (2)  *Age category:* Adult  *Diagnosis:* Mixed diagnoses  *Offender type:* NS  *Controls:* NA | *Outcome measure:* Hearth rate  *Assessment method:* Biosensor (smartwatches with photoplethysmography sensor)  *Function:* Intervention  *Stimulus/task:* NA | Biocueing seems a helpful addition to increasing interoceptive awareness among forensic outpatients. However, not all patients benefit from the current intervention and, more specifically, from its behavioral support component aimed at enhancing emotion regulation |
| Thijssen and Kiehl (2017) | To examine the association between psychopathic traits and functional connectivity in incarcerated male adolescents | *Design*: Cross-sectional study  *Country:* USA  *Setting:* Correctional facility  *Follow-up:* NA | *N:* 177  *Gender:* M  *Age category:* Juvenile  *Diagnosis:* Psychopathy/psychopathic traits, substance use disorder  *Offender type:* NS  *Controls:* NA | *Outcome measure:* Brain activity  *Assessment method:* fMRI  *Function:* Etiologic  *Stimulus/task:* NA (resting-state fMRI) | Comparable to adult psychopathy, adolescent psychopathic traits were associated with networks implicated in self-referential thought, moral behavior, cognition, and saliency detection, functions that have previously been reported to be disrupted in adult psychopaths |
| Thijssen et al. (2017) | To examine the association between cannabis and alcohol use duration and resting-state functional connectivity in a large sample of male juvenile delinquents | *Design*: Cross-sectional study  *Country:* USA  *Setting:* Correctional facility  *Follow-up:* NA | *N:* 201  *Gender:* M  *Age category:* Juvenile  *Diagnosis:* Mixed diagnoses  *Offender type:* NS  *Controls:* NA | *Outcome measure:* Brain activity  *Assessment method:* fMRI  *Function:* Etiologic  *Stimulus/task:* NA (resting-state fMRI) | Adolescent cannabis and alcohol use are associated with widespread differences in resting-state time course power spectra, which may persist even after abstinence |
| Tikasz et al. (2016) | To identify the brain regions with greater neurofunctional alterations, as detected by fMRI during an emotion processing task, of men with schizophrenia who had engaged in violent behavior compared with those who had not | *Design*: Case-control study  *Country:* Canada  *Setting:* Outpatient clinic  *Follow-up:* NA | *N:* 20  *Gender:* M  *Age category:* Adult  *Diagnosis:* Schizophrenia  *Offender type:* Violent offenders  *Controls:* M (19): Outpatients, non-violent, M (21): Healthy controls | *Outcome measure:* Brain activity  *Assessment method:* fMRI  *Function:* Etiologic  *Stimulus/task:* Viewing positive, negative and neutral images | Violent men with schizophrenia displayed specific increases in anterior cingulate cortex (ACC) activity in response to negative images. Given the role of the ACC in information integration, results indicate a specific dysfunction in the processing of negative emotions that may trigger violent behavior in men with schizophrenia |
| Tillem et al. (2019) | To examine the optimality of neural network organization in psychopathy with a minimum spanning tree graph analysis to resting-state fMRI data in male inmates | *Design*: Cross-sectional study  *Country:* USA  *Setting:* Correctional facility  *Follow-up:* NA | *N:* 847  *Gender:* M  *Age category:* Adult  *Diagnosis:* Psychopathy/psychopathic traits  *Offender type:* NS  *Controls:* NA | *Outcome measure:* Brain activity  *Assessment method:* fMRI  *Function:* Etiologic  *Stimulus/task:* NA (resting-state fMRI) | Psychopathy is related to alterations in the overall organization of neural networks. Specifically, individuals higher in psychopathy show more efficiently organized dorsal attention networks and a reduction in the centrality of subcortical structures in global information flow |
| Tillem et al. (2016) | To examine time-frequency EEG phase coherence in response to a picture-viewing paradigm that manipulated picture familiarity to assess neural changes in processing based on perceptual demands in psychopathic offenders | *Design*: Cross-sectional study  *Country:* USA  *Setting:* Correctional facility  *Follow-up:* NA | *N:* 99  *Gender:* M  *Age category:* Adult  *Diagnosis:* Psychopathy/psychopathic traits  *Offender type:* NS  *Controls:* NA | *Outcome measure:* Brain activity  *Assessment method:* EEG  *Function:* Etiologic  *Stimulus/task:* Picture-viewing paradigm that manipulated picture familiarity | Psychopathy-related differences in the theta response, an index of readiness to perceive and integrate sensory information, were found. These data provide further evidence that psychopathic offenders have disrupted integration of sensory information |
| Tonnaer et al. (2017) | To investigate differences in brain responses during anger provocation or anger engagement, as well as anger regulation or distraction from anger, in male violent offenders and non-offender controls | *Design*: Case-control study  *Country:* The Netherlands  *Setting:* Inpatient clinic  *Follow-up:* NA | *N:* 18  *Gender:* M  *Age category:* Adult  *Diagnosis:* Mixed diagnoses  *Offender type:* Violent offenders  *Controls:* M (16): Non-offenders | *Outcome measure:* Brain activity  *Assessment method:* fMRI  *Function:* Etiologic  *Stimulus/task:* Provocation and regulation task using audiotaped angry, happy, and neutral scenarios | Results suggest increased initiation to regulation as indicated by increased vlPFC activity during anger engagement and less during anger distraction in violent offenders. Additionally, when explicitly instructed to regulate using distraction, results hint at general emotion regulation impairments in violent offenders |
| Vaidyanathan et al. (2011) | To examine affective priming deficits in relation to factors of psychopathy and symptoms of antisocial personality disorder using startle reflex methods in male prisoners | *Design*: Cross-sectional study  *Country:* USA  *Setting:* Correctional facility  *Follow-up:* NA | *N:* 108  *Gender:* M  *Age category:* Adult  *Diagnosis:* Psychopathy, antisocial personality disorder  *Offender type:* NS  *Controls:* NA | *Outcome measure:* Startle blink response  *Assessment method:* EMG  *Function:* Etiologic  *Stimulus/task:* Visual stimuli (pleasant, neutral, aversive) and noise probes | Deficits in defensive reactivity are linked specifically to the affective-interpersonal features of psychopathy and not to the antisocial deviance features represented most strongly in antisocial personality disorder |
| van den Bos et al. (2014) | To investigate the behavioral and neural processes underlying social interactions of juvenile delinquents and a matched control group | *Design*: Case-control study  *Country:* Germany  *Setting:* Correctional facility and inpatient clinic  *Follow-up:* NA | *N:* 17  *Gender:* M  *Age category:* Adult and juvenile  *Diagnosis:* Not explicitly stated  *Offender type:* NS  *Controls:* M (17): Healthy controls | *Outcome measure:* Brain activity  *Assessment method:* fMRI  *Function:* Etiologic  *Stimulus/task:* Playing the mini-Ultimatum Game | Juvenile delinquents with severe antisocial behavior process norm violations adequately but may have difficulties with attending spontaneously to relevant features of the social context during interactions |
| Veit et al. (2002) | To investigate the hypothesis that underactivity of the frontolimbic circuitry underlies psychopathic behavior and its overactivity characterizes social phobics | *Design*: Case-control study  *Country:* Germany  *Setting:* NS  *Follow-up:* NA | *N:* 4  *Gender:* M  *Age category:* Adult  *Diagnosis:* Psychopathy, antisocial personality disorder  *Offender type:* NS  *Controls:* M (4): Social phobics, M (7): Healthy controls | *Outcome measure:* Brain activity  *Assessment method:* fMRI  *Function:* Etiologic  *Stimulus/task:* Differential aversive delay conditioning with neutral faces as conditioned and painful pressure as unconditioned stimuli | A hypoactive frontolimbic circuit may represent the neural correlate of psychopathic behavior, whereas an overactive frontolimbic system may underly social fear |
| Veit et al. (2013) | To investigate to which extent the different facets of the psychopathy construct contribute to the fear conditioning deficits observed in psychopaths | *Design*: Cross-sectional study  *Country:* Germany  *Setting:* Inpatient clinic  *Follow-up:* NA | *N:* 14  *Gender:* M  *Age category:* Adult  *Diagnosis:* Psychopathy, antisocial personality disorder  *Offender type:* Violent offenders  *Controls:* NA | *Outcome measure:* ERP, skin conductance  *Assessment method:* Theraprax Neurofeedback system with EEG, EOG (electrooculography) and SCR applications  *Function:* Etiologic  *Stimulus/task:* Classical delayed fear conditioning paradigm: Two neutral grey-scale male faces were used as conditioned stimuli, one face being paired with an unconditioned stimulus consisting of an electric shock | Study findings hint at segregated emotional and cognitive processing during implicit fear learning in psychopathic subtypes |
| Veit et al. (2010) | To investigate which cerebral areas associated with aggression and aggression control are active in psychiatric inmates with psychopathy during retaliation and opponent observing | *Design*: Cross-sectional study  *Country:* Germany  *Setting:* Inpatient clinic  *Follow-up:* NA | *N:* 10  *Gender:* M  *Age category:* Adult  *Diagnosis:* Psychopathy  *Offender type:* Mixed offender types  *Controls:* NA | *Outcome measure:* Brain activity  *Assessment method:* fMRI  *Function:* Etiologic  *Stimulus/task:* Mechanical aversive (pain) stimuli applied using a plastic cylinder (diameter of 7 mm) moved by air pressure modulated by a pneumatic device (Dokoh-Pneu, Erlangen; velocities: 2 m/s to 20m/s) | Reactive aggression is more related to antisocial behavior and anger management than with emotional and interpersonal characteristics of psychopathy and suggest that two separate brain activation patterns seem to account for these two behavioral dispositions |
| Venables and Patrick (2014) | To investigate differential relations of separable diagnostic facets of PCL-R psychopathy with P3 amplitude in an offender sample | *Design*: Cross-sectional study  *Country:* USA  *Setting:* Inpatient clinic  *Follow-up:* NA | *N:* 154  *Gender:* M  *Age category:* Adult  *Diagnosis:* Psychopathy  *Offender type:* NS  *Controls:* NA | *Outcome measure:* Brain activity  *Assessment method:* EEG  *Function:* Etiologic  *Stimulus/task:* Visual oddball task that included incidental novel as well as task-relevant target stimuli | Impulsive-antisocial features of psychopathy selectively exhibit an inverse relationship with P3 amplitude |
| Venables et al. (2015) | To examine the relationship between psychopathy factors and electrocortical response to emotional and neutral pictures in male offenders | *Design*: Cross-sectional study  *Country:* USA  *Setting:* Inpatient clinic  *Follow-up:* NA | *N:* 139  *Gender:* M  *Age category:* Adult  *Diagnosis:* Psychopatic traits, substance use disorder  *Offender type:* Violent offenders  *Controls:* NA | *Outcome measure:* Brain activity  *Assessment method:* EEG  *Function:* Etiologic  *Stimulus/task:* Visual stimuli (pictures consisting pleasant, neutral, and unpleasant scenes) and noise probes | Impulsive-antisocial features of the PCL-R were associated with reduced amplitude of earlier P3 brain response to pictures regardless of valence, whereas the affective-interpersonal dimension was associated with reductions in late positive potential response to aversive pictures. This supports the Two-Process theory |
| Verona and Bresin (2015) | To investigate whether aggression proneness, as defined by trait aggression and anger, was linked to the interplay in functioning of the Negative Valence and Cognitive Systems, manifested in disruptions in emotional and inhibitory control processing | *Design*: Cross-sectional study  *Country:* USA  *Setting:* Correctional facility, inpatient clinic and outpatient clinic  *Follow-up:* NA | *N:* 67  *Gender:* M (47) + F (20)  *Age category:* Adult  *Diagnosis:* Psychopathy, psychopatic traits  *Offender type:* Violent offenders  *Controls:* NA | *Outcome measure:* ERP  *Assessment method:* EEG  *Function:* Etiologic  *Stimulus/task:* Emotional linguistic Go-NoGo task | Results provide evidence that tendencies toward angry and aggressive behavior relate to reduced inhibitory control processing (no-go P3) specifically during relevant threat-word blocks, suggesting deterioration of cognitive control by acute or sustained threat sensitivity |
| Verona et al. (2013) | To investigate the association between psychopathy and an affect-startle paradigm in a sample of incarcerated women | *Design*: Case-control study  *Country:* USA  *Setting:* Correctional facility  *Follow-up:* NA | *N:* 24  *Gender:* F  *Age category:* Adult  *Diagnosis:* Psychopathy  *Offender type:* NS  *Controls:* F (24): Offenders, low psychopathy | *Outcome measure:* Facial (eyeblink) electromyographic activity  *Assessment method:* EMG  *Function:* Etiologic  *Stimulus/task:* Visual stimuli (pleasant, neutral, and unpleasant pictures) and blink-eliciting noise probes | Women scoring high on psychopathy exhibited deficits in startle reactivity to unpleasant pictures, especially with regard to victim-distress scenes, highlighting a specific insensitivity to the vicarious distress of others |
| Verona et al. (2012) | To compare cognitive-emotional processing in psychopathy and antisocial personality disorder | *Design*: Case-control study  *Country:* USA  *Setting:* Correctional facility  *Follow-up:* NA | *N:* 30  *Gender:* M  *Age category:* Adult  *Diagnosis:* Psychopathy, antisocial personality disorder  *Offender type:* NS  *Controls:* M (15): Offenders, non-psychopathic and no ASPD | *Outcome measure:* ERP  *Assessment method:* EEG  *Function:* Etiologic  *Stimulus/task:* Emotional-linguistic Go and NoGo trials | Results revealed disruptions in interactions between negative emotional processing and the inhibitory control processes that govern appropriate behavior in psychopathy and antisocial personality disorder |
| Verona et al. (2004) | To investigate physiological reactions to emotional sounds in prisoners selected according to scores on the 2 factors of Hare’s PCL-R | *Design*: Case-control study  *Country:* USA  *Setting:* Correctional facility  *Follow-up:* NA | *N:* 18  *Gender:* M  *Age category:* Adult  *Diagnosis:* Psychopathy  *Offender type:* NS  *Controls:* M (15): Offenders, only high Factor 1 score, M (17): Offenders, only high Factor 2 score, M (18): Offenders, non-psychopathic | *Outcome measure:* Skin conductance, hearth rate, corrugator (frown) and zygomatic (smile) electromyographic measures  *Assessment method:* (Coulbourn) bioamplifiers with skin conductance, heart rate and EMG applications  *Function:* Etiologic  *Stimulus/task:* Nine sound clips (emotional sounds) of 6-s duration chosen from the IADS | Results indicate abnormal reactivity to both positive and negative emotional stimuli in psychopathic individuals, and suggest differing roles for the 2 facets of psychopathy in affective processing deviations |
| Verschuere et al. (2005) | To examine the moderating role of psychopathic features on responding to concealed information in a prison sample | *Design*: Case-control study  *Country:* Belgium  *Setting:* Correctional facility  *Follow-up:* NA | *N:* 40  *Gender:* M  *Age category:* Adult  *Diagnosis:* Psychopathy/psychopathic traits  *Offender type:* NS  *Controls:* F (27): Healthy controls | *Outcome measure:* Skin conductance, heart rate, respiration  *Assessment method:* Coulbourn bioamplifier with skin conductance, heart rate, and respiration applications  *Function:* Etiologic  *Stimulus/task: Concealed information test* | Orienting accounts for the concealed information effect in both undergraduates and prisoners, and personality variables, such as psychopathic traits, may moderate responding to concealed information |
| Verschuere et al. (2007) | To investigate the association between antisociality and orienting to concealed information and its impact on the detection efficiency of the Concealed Information Test | *Design*: Case-control study  *Country:* Belgium  *Setting:* Correctional facility  *Follow-up:* NA | *N:* 48  *Gender:* M  *Age category:* Adult  *Diagnosis:* Psychopathy/psychopathic traits  *Offender type:* Mixed offender types  *Controls:* M (31): Healthy controls | *Outcome measure:* Skin conductance, heart rate, respiration  *Assessment method:* Lablinc V Coulbourn  *Function:* Etiologic  *Stimulus/task: Concealed information test* | The autonomic under arousal associated with antisociality does not deteriorate the detection efficiency of the Concealed Information Test (CIT); good accuracy of the CIT can be obtained in criminal populations |
| Vila-Ballo et al. (2014) | To investigate performance monitoring using event-related brain potentials in juvenile non-psychopathic violent offenders compared with a matched control group | *Design*: Case-control study  *Country:* Spain  *Setting:* Correctional facility  *Follow-up:* NA | *N:* 17  *Gender:* M  *Age category:* Adult  *Diagnosis:* Conduct disorder  *Offender type:* Violent offenders  *Controls:* M (17): Healthy controls | *Outcome measure:* Brain activity  *Assessment method:* EEG  *Function:* Etiologic  *Stimulus/task:* Modified variant of the Eriksen flanker task | Different aspects of executive function were affected in the studied offenders, including error processing (reduced ERN) and response inhibition (reduced N2and P3). However, error awareness and compensatory post-error adjustment processes (error correction) were unaffected |
| Vincent et al. (2018) | To investigate the differences in the neural responses associated with drug craving among high-risk young offenders with histories of abuse of stimulants and other drugs as a function of psychopathic traits | *Design*: Case-control study  *Country:* USA  *Setting:* Correctional facility  *Follow-up:* NA | *N:* 40  *Gender:* M  *Age category:* Juvenile  *Diagnosis:* Psychopathic traits, substance use disorder  *Offender type:* NS  *Controls:* M (10): Offenders, no stimulant use disorder | *Outcome measure:* Brain activity  *Assessment method:* fMRI  *Function:* Etiologic  *Stimulus/task:* Drug-cue exposure task | Psychopathy scores, predominantly callous-unemotional traits, are negatively associated with hemodynamic response related to drug craving in the amygdala and anterior cingulate gyrus in youth with a history of stimulant abuse |
| Völlm et al. (2007) | To investigate the effects of positive (monetary reward) and negative (monetary loss) outcomes on BOLD responses in two target selection tasks | *Design*: Case-control study  *Country:* UK  *Setting:* Inpatient clinic  *Follow-up:* NA | *N:* 8  *Gender:* M  *Age category:* Adult  *Diagnosis:* Personality disorder  *Offender type:* NS  *Controls:* M (14): Healthy controls | *Outcome measure:* Brain activity  *Assessment method:* fMRI  *Function:* Etiologic  *Stimulus/task:* Reward and loss task, Go-NoGo task | Results suggest dysfunctional responses to rewarding and aversive stimuli in cluster B personality disorder but do not support the notion of hypersensitivity to reward and hyposensitivity to loss |
| Völlm et al. (2010) | To identify brain areas associated with behavioural inhibition and reward in healthy and ASPD individuals and differences in activations between these two groups | *Design*: RCT, cross-sectional study  *Country:* UK, Australia  *Setting:* Correctional facility and inpatient clinic  *Follow-up:* NA | *N:* 25  *Gender:* M  *Age category:* Adult  *Diagnosis:* Personality disorder  *Offender type:* NS  *Controls:* M (25): Healthy controls | *Outcome measure:* Brain activity  *Assessment method:* fMRI  *Function:* Monitoring  *Stimulus/task:* Go-NoGo task, reward/no-reward task, a facial recognition task and an empathy task | ASPD involves altered serotonin modulation of reward, but not motor inhibition pathways and altered DLPFC, ACC and OFC function. Altered serotonergic modulation of reward pathways seen in the ASPD group raises the possibility that targeting serotonin systems may be therapeutic |
| Volman et al. (2016) | To test whether exerting control over emotionally relevant actions is reflected by reduced functionality of the anterior prefrontal cortex (aPFC), amygdala circuit in psychopathic individuals, suggesting less prefrontal regulation of emotional actions | *Design*: Case-control study  *Country:* The Netherlands  *Setting:* Inpatient clinic  *Follow-up:* NA | *N:* 17  *Gender:* M  *Age category:* Adult  *Diagnosis:* Psychopathy  *Offender type:* Violent offenders  *Controls:* M (21): Healthy controls | *Outcome measure:* Brain activity  *Assessment method:* fMRI  *Function:* Etiologic  *Stimulus/task:* fMRI-adapted approach-avoidance task requiring rule-driven control over rapid emotional responses | Reduced prefrontal coordination underlies reduced behavioral control in psychopathic offenders during emotionally provoking situations. The modulatory role of endogenous testosterone on the aPFC–amygdala circuit suggests a neurobiological substrate of individual differences that is relevant for the advancement of treatment and the reduction of recidivism |
| von Borries (2010) | To examine the relationship between error monitoring and reinforcement learning in individuals diagnosed with psychopathy by investigating the rERN (response error related negativity) and fERN (feedback error related negativity) and the relationship between the two while learning progresses | *Design*: Case-control study  *Country:* The Netherlands  *Setting:* Inpatient clinic  *Follow-up:* NA | *N:* 13  *Gender:* M  *Age category:* Adult  *Diagnosis:* Psychopathy  *Offender type:* Violent offenders  *Controls:* M (18): Healthy controls | *Outcome measure:* Brain activity  *Assessment method:* EEG  *Function:* Etiologic  *Stimulus/task:* Probabilistic learning task | Disturbed error-monitoring processes play a central role in the often reported learning deficits in individuals with psychopathy |
| Wahlund et al. (2010) | To investigate subjective ratings and skin conductance responses in mentally disordered offenders with various diagnoses but without psychopathy, specifically recruited from the forensic psychiatric system | *Design*: Case-control study  *Country:* Sweden  *Setting:* Outpatient clinic  *Follow-up:* NA | *N:* 41  *Gender:* M  *Age category:* Adult  *Diagnosis:* Personality disorder  *Offender type:* NS  *Controls:* M (20): Healthy controls | *Outcome measure:* Skin conductance  *Assessment method:* Psylab SC5, Psylab Stand Alone Monitor, SAM instruments  *Function:* Etiologic  *Stimulus/task:* Neutral and negative pictures from the International Affective Picture System (IAPS) | Offenders showed significantly lower SCRs and subjective ratings than the control group. There was no significant difference between antisocial and non-antisocial offenders, indicating that antisocial behavior might not be a differential factor. Thus, attenuated emotional responses may be a characteristic shared by mentally disordered offenders overall |
| Walter et al. (2007) | To investigate the neural correlates of deficits of sexual and emotional arousal in paedophiles | *Design*: Case-control study  *Country:* Germany  *Setting:* Inpatient clinic  *Follow-up:* NA | *N:* 13  *Gender:* M  *Age category:* Adult  *Diagnosis:* Paedophilia  *Offender type:* Sex offenders: Child victims  *Controls:* M (14): Healthy controls | *Outcome measure:* Brain activity  *Assessment method:* fMRI  *Function:* Etiologic  *Stimulus/task:* Visual stimulation with emotional and erotic pictures | Abnormal activation in subcortical and cortical regions was observed in paedophilia during visual-erotic stimulation with adults. Because these regions are implicated in the vegetative-autonomic and emotional components of sexual arousal, results indicate possible neural correlates of lack of sexual interest toward adults in paedophilic patients |
| Weidacker et al. (2022) | To examine event-related fMRI data of three groups of men performing on a trial-by-trial version of a color-word Stroop task: paedophiles with a history of child sex offenses (CSO), paedophiles without a history of CSO, and non-paedophilic, non-offending healthy controls | *Design*: Case-control study  *Country:* Germany  *Setting:* Inpatient clinic or outpatient clinic (not clear)  *Follow-up:* NA | *N:* 11  *Gender:* M  *Age category:* Adult  *Diagnosis:* Paedophilia  *Offender type:* Sex offenders: Child victims  *Controls:* M (8): Paedophiles without child sexual offending, M (10): Healthy controls | *Outcome measure:* Brain activity  *Assessment method:* fMRI  *Function:* Etiologic  *Stimulus/task:* Stroop task | Results highlight inhibition deficits in offending as compared to non-offending paedophiles or healthy men and suggest that functional alterations in attention reallocation and impulse suppression/control may moderate the risk for committing child sex offences in men suffering from paedophilia |
| Wilson et al. (2011) | To investigate the level of agreement, specifically with regard to the identification of paedophilia, between four methods or indices typically used in sexual offender assessments (actuarial assessment of risk (i.e., the RRASOR), phallometric assessment, expert diagnosis, and DSM-IV-TR-based diagnoses) and the relationship between each of these four predictor variables and sexual offence recidivism | *Design*: Cross-sectional and cohort study  *Country:* Canada  *Setting:* Inpatient clinic  *Follow-up:* Mean duration: 8.8 years | *N:* 130  *Gender:* M  *Age category:* Adult  *Diagnosis:* Paedophilia  *Offender type:* Sex offenders: Child victims  *Controls:* NA | *Outcome measure:* Change in penis circumference  *Assessment method:* PPG  *Function:* Diagnostic and prognostic  *Stimulus/task:* Slides of single, nude adults, pubescents, and prepubescents of each gender, as well as neutral (scenery) slides | Inconsistency exists in diagnosing paedophilia, leading to diminished accuracy in risk assessment |
| Wong et al. (1997) | To examine whether different violent offending behaviours are associated with different clinical and neuroimaging profiles | *Design*: Case-control study  *Country:* UK  *Setting:* Inpatient clinic  *Follow-up:* NA | *N:* 20  *Gender:* M  *Age category:* Adult  *Diagnosis:* Schizophrenia, schizoaffective disorder  *Offender type:* Violent offenders  *Controls:* M (19): Non-repetitive violent offenders | *Outcome measure:* Brain activity  *Assessment method:* EEG  *Function:* Etiologic  *Stimulus/task:* NA | Different structural and metabolic changes in the brain were associated with different violent offending behaviours |
| Wong et al. (1994) | To investigate possible associations of focal electrical or structural abnormalities in forensic inpatients and violence arising in the context of their offending behavior | *Design*: Case-control study  *Country:* UK  *Setting:* Inpatient clinic  *Follow-up:* NA | *N:* 110  *Gender:* M  *Age category:* Adult  *Diagnosis:* Mixed diagnoses  *Offender type:* Violent offenders  *Controls:* M (202): Inpatients, moderate violence, M (60): Inpatients, low violence | *Outcome measure:* Brain activity  *Assessment method:* EEG  *Function:* Etiologic  *Stimulus/task:* NA | High violence rating scores are associated with temporal lobe abnormalities on CT and abnormal temporal electrical discharges on EEG |
| Wong et al. (1994) | To investigate the relationship between epilepsy and violence in mentally abnormal offenders | *Design*: Cross-sectional study  *Country:* UK  *Setting:* Inpatient clinic  *Follow-up:* NA | *N:* 21  *Gender:* M (16) + F (5)  *Age category:* Adult  *Diagnosis:* Personality disorder, schizophrenia  *Offender type:* Mixed offender types  *Controls:* NA | *Outcome measure:* Brain activity  *Assessment method:* EEG  *Function:* Etiologic  *Stimulus/task:* NA | There was no obvious association between criminal behaviour and seizures in the studied patients with epilepsy |
| Wormith (1986) | To investigate the sexual assessment technology penile plethysmography in terms of measurement, reliability, validity, self-reported arousal, faking, and instructional procedures in three groups of incarcerated offenders: rapists, paedophiles, and non-sex offenders | *Design*: Case-control study  *Country:* Canada  *Setting:* Correctional facility  *Follow-up:* NA | *N:* 12  *Gender:* M  *Age category:* Adult  *Diagnosis:* Paedophilia  *Offender type:* Sex offenders: Child victims  *Controls:* M (12): Sex offenders with adult victim, M (12): Non-sex offenders | *Outcome measure:* Change in penis circumference  *Assessment method:* PPG  *Function:* Diagnostic  *Stimulus/task:* Visual sexual stimuli | In practical terms of diagnosis, misclassifications were frequent (about 30%) |
| Wormith et al. (1988) | To investigate the relationship between intelligence, alcohol, suppression instruction, and deviant sexual arousal in a sample of sex offenders | *Design*: Case-control study  *Country:* Canada  *Setting:* Inpatient clinic  *Follow-up:* NA | *N:* 23  *Gender:* M  *Age category:* Adult  *Diagnosis:* Paedophilia, paraphilia  *Offender type:* Sex offenders: Child and adult victims  *Controls:* M (NS): Sex offenders, non-rapists | *Outcome measure:* Change in penis circumference  *Assessment method:* PPG  *Function:* Etiologic  *Stimulus/task:* Narrative (audio) sexual stimuli | While under the influence of alcohol, non-rapists showed a decrease, and rapists an increase in sexual response. The latter may be more tolerant to alcohol, may be less able to control their sexual arousal when intoxicated, or may simply become sexually disinhibited when under the influence of alcohol |
| Yoder et al. (2015) | To examine the influence of task demands on moral evaluation in psychopathy in incarcerated males | *Design*: Cross-sectional study  *Country:* USA  *Setting:* Correctional facility  *Follow-up:* NA | *N:* 88  *Gender:* M  *Age category:* Adult  *Diagnosis:* Psychopathy/psychopathic traits  *Offender type:* NS  *Controls:* NA | *Outcome measure:* Brain activity  *Assessment method:* fMRI  *Function:* Etiologic  *Stimulus/task:* Visual moral evaluation task | Hemodynamic activity and neural coupling within the salience network are disrupted in psychopathy, and the effects of psychopathy on moral evaluation are influenced by attentional demands |
| Yoder et al. (2021) | To examine socio-emotional processing in response to third-party morally laden interactions in female offenders | *Design*: Cross-sectional study  *Country:* USA  *Setting:* Correctional facility  *Follow-up:* NA | *N:* 107  *Gender:* F  *Age category:* Adult  *Diagnosis:* Psychopathy/psychopathic traits  *Offender type:* NS  *Controls:* NA | *Outcome measure:* Brain activity  *Assessment method:* fMRI  *Function:* Etiologic  *Stimulus/task:* Visual moral decision making task | In females, psychopathy is associated with normal behavioral accuracy and confidence but alterations in neural network activity during moral decision-making |
| Yoder et al. (2022) | To examine neural responses and functional connectivity associated with empathy and affective perspective-taking in female inmates with various levels of psychopathic traits | *Design*: Cross-sectional study  *Country:* USA  *Setting:* Correctional facility  *Follow-up:* NA | *N:* 109  *Gender:* F  *Age category:* Adult  *Diagnosis:* Psychopathy/psychopathic traits  *Offender type:* NS  *Controls:* NA | *Outcome measure:* Brain activity  *Assessment method:* fMRI  *Function:* Etiologic  *Stimulus/task:* Viewing hands and feet in painful or non-painful situations while adopting a first person or third person perspective | Psychopathic traits in incarcerated females are associated with atypical functional connectivity within the salience network during pain-empathy processing and within the social cognition network during affective perspective-taking |
| Zhang et al. (2017) | To analyze the changes of the distribution of powers within different brain regions based on rsfMRI measurements and to examine whether the new paradigm can identify the difference of the powers of brain regions between young subjects with CD and young healthy controls | *Design*: Case-control study  *Country:* China  *Setting:* Correctional facility  *Follow-up:* NA | *N:* 18  *Gender:* M  *Age category:* Juvenile  *Diagnosis:* Conduct disorder  *Offender type:* NS  *Controls:* M (18): Healthy controls | *Outcome measure:* Brain activity  *Assessment method:* fMRI  *Function:* Etiologic  *Stimulus/task:* NA | The results of the analysis of the changes in power exhibited that there were significant power differences in some pairs of brain regions between the CD and TD groups, indicating a change in the power distribution. Results also suggest that the total power consumption of brain networks in CD patients is less than that observed in the TD group |
| Zhou et al. (2016) | To investigate whether participants with CD have altered hemodynamic activity under resting-state conditions | *Design*: Case-control study  *Country:* China  *Setting:* Correctional facility  *Follow-up:* NA | *N:* 18  *Gender:* M  *Age category:* Juvenile  *Diagnosis:* Conduct disorder  *Offender type:* NS  *Controls:* M (18): Healthy controls | *Outcome measure:* Brain activity  *Assessment method:* fMRI  *Function:* Etiologic  *Stimulus/task:* NA | Youth with CD displayed widespread functional abnormalities in emotion-related and visual cortical regions in the resting state. These results suggest that deficits in the intrinsic activity of resting state networks may contribute to the etiology of CD |
| Zhou et al. (2015) | To investigate DMN connectivity in male adolescents with pure CD compared to typically developing controls | *Design*: Case-control study  *Country:* China  *Setting:* Correctional facility  *Follow-up:* NA | *N:* 18  *Gender:* M  *Age category:* Juvenile  *Diagnosis:* Conduct disorder  *Offender type:* NS  *Controls:* M (18): Healthy controls | *Outcome measure:* Brain activity  *Assessment method:* fMRI  *Function:* Etiologic  *Stimulus/task:* NA | Results suggest that deficits in DMN functional connectivity may serve as a biomarker of CD |
| Zijlmans et al. (2018) | To test in multi-problem young adults the hypothesis that psychopathic traits are related to amygdala and vmPFC activity during moral evaluation and to explore the relation between psychopathic traits and other regions consistently implicated in moral evaluation | *Design*: Case-control study  *Country:* The Netherlands  *Setting:* Correctional facility  *Follow-up:* NA | *N:* 100  *Gender:* M  *Age category:* Adult  *Diagnosis:* Psychopathy  *Offender type:* Violent offenders  *Controls:* M (22): Healthy controls | *Outcome measure:* Brain activity  *Assessment method:* fMRI  *Function:* Etiologic  *Stimulus/task:* Moral evaluation task | Results are consistent with altered vmPFC function during moral evaluation in psychopathy, but we did not find evidence for amygdala involvement. Results indicate the affective callous-unemotional trait of psychopathy may be related to widespread altered activation patterns during moral evaluation in multi-problem young adults |
| Zijlmans et al. (2019) | To investigate the relationship between psychopathic traits and both early and late error-related brain activity in an at-risk sample of male young adults | *Design*: Case-control study  *Country:* The Netherlands  *Setting:* Outpatient clinic  *Follow-up:* NA | *N:* 115  *Gender:* M  *Age category:* Adult  *Diagnosis:* Psychopathy/psychopathic traits  *Offender type:* NS  *Controls:* M (26): Healthy controls | *Outcome measure:* Brain activity  *Assessment method:* fMRI  *Function:* Etiologic  *Stimulus/task:* Eriksen–Flanker task | Results indicate dysfunctional error-processing in multi-problem young adults compared to controls. However, within the multi-problem sample no evidence was found for a relationship between psychopathic traits and dysfunctional error-processing |
| Zijlmans et al. (2021) | To examine whether selected measures of autonomic functioning, functional neuroimaging and electroencephalography predict overall and serious recidivism in a sample of delinquent young adults | *Design*: Cohort study  *Country:* The Netherlands  *Setting:* Outpatient clinic  *Follow-up:* 14 months | *N:* 127  *Gender:* M  *Age category:* Adult  *Diagnosis:* Not explicitly stated  *Offender type:* NS  *Controls:* NA | *Outcome measure:* Brain activity, heart rate (variability)  *Assessment method:* EEG, fMRI, ECG  *Function:* Etiologic  *Stimulus/task:* fMRI: Go-NoGo task; EEG: Eriksen–Flanker task | Demographic and behavioural characteristics longitudinally predicted recidivism in delinquent male young adults, and neurobiological measures (autonomic functioning and EEG) improved the models |

NA = Not Applicable; NS = Not Specified; PPG = Penile plethysmography
