# Supplementary material for: Using physiological biomarkers in forensic psychiatry: a scoping review
Source: Front Psychiatry. 2025 Apr 29;16:1580615. doi: 10.3389/fpsyt.2025.1580615 (PMC12069285; doi:10.3389/fpsyt.2025.1580615)
Supplement: Supplementary file 2 [file DataSheet1.docx]

Supplementary Material

Contents

[1 Appendix I: Full search terms MEDLINE (PubMed) 2](#_Toc190946119)

[2 Appendix II: Search strategy Google Scholar (Publish or Perish) 4](#_Toc190946120)

[3 Appendix III: Data extraction instrument 5](#_Toc190946121)

# Appendix I: Full search terms MEDLINE (PubMed)

Search August 24, 2023

| Search | Query | Records retrieved |
| --- | --- | --- |
| S1 | ("marker"[All Fields] OR "markers"[All Fields] OR "biomarker*"[All Fields] OR ("eye tracking technology"[MeSH Terms] OR ("eye tracking"[All Fields] AND "technology"[All Fields]) OR "eye tracking technology"[All Fields] OR ("eye"[All Fields] AND "tracking"[All Fields]) OR "eye tracking"[All Fields]) OR ("magnetic resonance imaging"[MeSH Terms] OR ("magnetic"[All Fields] AND "resonance"[All Fields] AND "imaging"[All Fields]) OR "magnetic resonance imaging"[All Fields] OR "fmri"[All Fields]) OR (("brain"[MeSH Terms] OR "brain"[All Fields] OR "brains"[All Fields] OR "brain s"[All Fields]) AND ("activable"[All Fields] OR "activate"[All Fields] OR "activated"[All Fields] OR "activates"[All Fields] OR "activating"[All Fields] OR "activation"[All Fields] OR "activations"[All Fields] OR "activator"[All Fields] OR "activator s"[All Fields] OR "activators"[All Fields] OR "active"[All Fields] OR "actived"[All Fields] OR "actively"[All Fields] OR "actives"[All Fields] OR "activities"[All Fields] OR "activity s"[All Fields] OR "activitys"[All Fields] OR "motor activity"[MeSH Terms] OR ("motor"[All Fields] AND "activity"[All Fields]) OR "motor activity"[All Fields] OR "activity"[All Fields])) OR ("neuroimaging"[MeSH Terms] OR "neuroimaging"[All Fields] OR ("brain"[All Fields] AND "imaging"[All Fields]) OR "brain imaging"[All Fields]) OR "neuroimag*"[All Fields] OR (("skin"[MeSH Terms] OR "skin"[All Fields]) AND ("behavior"[MeSH Terms] OR "behavior"[All Fields] OR "conduct"[All Fields] OR "conducting"[All Fields] OR "conducts"[All Fields] OR "conductance"[All Fields] OR "conductances"[All Fields] OR "conducted"[All Fields] OR "conductibility"[All Fields] OR "conduction"[All Fields] OR "conductions"[All Fields] OR "conductive"[All Fields] OR "conductively"[All Fields] OR "conductivities"[All Fields] OR "electric conductivity"[MeSH Terms] OR ("electric"[All Fields] AND "conductivity"[All Fields]) OR "electric conductivity"[All Fields] OR "conductivity"[All Fields])) OR (("heartrate"[All Fields] OR "heartrates"[All Fields]) AND ("variabilities"[All Fields] OR "variability"[All Fields] OR "variable"[All Fields] OR "variable s"[All Fields] OR "variables"[All Fields] OR "variably"[All Fields])) OR ("wearability"[All Fields] OR "wearable"[All Fields] OR "wearables"[All Fields]) OR "temperat*"[All Fields] OR ("electroencephalography"[MeSH Terms] OR "electroencephalography"[All Fields] OR "eeg"[All Fields]) OR "plethysmogr*"[All Fields] OR "polygraph*"[All Fields]) | 4.190.229 |
| S2 | ("forens*"[All Fields] OR "crim*"[All Fields] OR "offen*"[All Fields] OR "prison*"[All Fields] OR "secur*"[All Fields] OR "pedoph*"[All Fields] OR "paraphil*"[All Fields] OR ("antisocial personality disorder"[MeSH Terms] OR ("antisocial"[All Fields] AND "personality"[All Fields] AND "disorder"[All Fields]) OR "antisocial personality disorder"[All Fields] OR "psychopathy"[All Fields] OR "psychopathies"[All Fields]) | 429.185 |
| S3 | ("psychiatr*"[All Fields] OR (("mental"[All Fields] OR "mentalities"[All Fields] OR "mentality"[All Fields] OR "mentalization"[MeSH Terms] OR "mentalization"[All Fields] OR "mentalizing"[All Fields] OR "mentalize"[All Fields] OR "mentalized"[All Fields] OR "mentally"[All Fields]) AND "illn*"[All Fields]) OR (("mental"[All Fields] OR "mentalities"[All Fields] OR "mentality"[All Fields] OR "mentalization"[MeSH Terms] OR "mentalization"[All Fields] OR "mentalizing"[All Fields] OR "mentalize"[All Fields] OR "mentalized"[All Fields] OR "mentally"[All Fields]) AND "disord*"[All Fields]) OR "psychopath*"[All Fields] OR "inpati*"[All Fields] OR "pedophil*"[All Fields] OR "paraphil*"[All Fields]) | 1.353.219 |
| S4 | S1 AND S2 AND S3 | 4.621 |
| S5 | Limiters: none | 4.621 |

# Appendix II: Search strategy Google Scholar (Publish or Perish)

| Source | Query |
| --- | --- |
| Google Scholar (Publish or Perish) | Marker\|Biomarkerǀ“Eye tracking”\|FMRI\|“Brain activation”\|”Brain imaging”\|Neuroimaging\|”Skin conductance”\|”Heart Rate variability”\|Wearable\|Temperature\|EEG\|Plethysmography\|Polygraph Forensic\|Criminal\|Offender\|Prisoner  Psychiatry\|”Mental illness” |

# Appendix III: Data extraction instrument

| 1. Study details and characteristics | |
| --- | --- |
| Author(s) | Indicate all authors as follows: “last name, first abbreviated name” |
| Title | Full title and subtitle |
| Year of publication | Indicate year of publication |
| Study reference | Bibliographic reference of the source of evidence |
| Country of origin | Indicate country/countries where the study was conducted |
| Study design | Type of study (e.g., randomized controlled trial, case-control study) |
|  | If applicable: duration of follow-up (in hours – years) |
| Objective(s) | Brief description of study objective |
| Context | Indicate study context (e.g., correctional facility, inpatient clinic) |
| Participants | N (number of participants) |
|  | Gender |
|  | Age category: adult, juvenile |
|  | Psychopathology (diagnosis, e.g. antisocial personality disorder, schizophrenia) |
|  | Offender type (e.g., violent offender, sex offender) |
| Controls (if applicable) | N (number of controls |
|  | Gender |
|  | Age category |
|  | Population: healthy controls/non-offenders, offenders, psychiatric (in)patients |
| 1. Evidence / result(s) extracted from study | |
| Biomarker | Outcome measure (e.g., brain activity, skin conductance level, penile circumference) |
|  | Assessment method (e.g., fMRI, EEG, penile plethysmography) |
|  | Function (etiologic, diagnostic, monitoring, intervention, prognostic, predictive) |
| Stimulus/task | If applicable: stimulus/task used as provocation during biomarker assessment (e.g., emotional processing task, sexual stimuli) |
| Main conclusion | Main conclusion of study (without commenting on the quality of the study and/or validity of the conclusion) |
